# Supplementary material for: Unstable TTTTA/TTTCA expansions in MARCH6 are associated with Familial Adult Myoclonic Epilepsy type 3
Source: Nat Commun. 2019 Oct 29;10:4919. doi: 10.1038/s41467-019-12763-9 (PMC6820781; doi:10.1038/s41467-019-12763-9)
Supplement: Supplementary file 1 — Supplementary Information [file 41467_2019_12763_MOESM1_ESM.pdf]

Supplementary Information

Unstable TTTTA/TTTCA expansions in *MARCH6* are associated with Familial Adult Myoclonic Epilepsy type 3

Rahel T. Florian, Florian Kraft, Elsa Leitão, Sabine Kaya, Stephan Klebe, Eloi Magnin, Anne-Fleur van Rootselaar, Julien Buratti, Theresa Kühnel, Christopher Schröder, Sebastian Giesselmann, Nikolai Tschernoster, Janine Altmueller, Anaide Lamiral, Boris Keren, Caroline Nava, Delphine Bouteiller, Sylvie Forlani, Ludmila Jornea, Regina Kubica, Tao Ye, Damien Plassard, Bernard Jost, Vincent Meyer, Jean-François Deleuze, Yannick Delpu, Mario D. M. Avarello, Lisanne S. Vijfhuizen, Gabrielle Rudolf, Edouard Hirsch, Thessa Kroes, Philipp S. Reif, Felix Rosenow, Christos Ganos, Marie Vidailhet, Lionel Thivard, Alexandre Mathieu, Thomas Bourgeron, Ingo Kurth, Haloom Rafehi, Laura Steenpass, Bernhard Horsthemke, FAME consortium, Eric LeGuern, Karl Martin Klein, Pierre Labauge, Mark F. Bennett, Melanie Bahlo, Jozef Gecz, Mark A. Corbett, Marina A.J. Tijssen, Arn M.J.M. van den Maagdenberg, Christel Depienne

Supplementary Fig. 1 Confirmation of TTTTA and TTTCA expansions in Families 1 (FAME3) and 5 (using exSTRa, STRetch and TRhist.

Supplementary Fig. 2 Pedigrees of FAME Families 5-13**,** tested negative for TTTTA/TTTCA expansion in *MARCH6*.

Supplementary Fig. 3 RP-PCR results corresponding to expansion carriers of Families 1-4.

Supplementary Fig. 4 Analysis of the microsatellite region, where the *MARCH6* expansion occurs, in healthy control individuals and during evolution.

Supplementary Fig. 5 Haplotyping of chr5 repeat expansion.

Supplementary Fig. 6 Visualization of extracted nanopore reads covering the expansion.

Supplementary Fig. 7 Distribution and interpretation of signals detected in blood samples by molecular combing.

Supplementary Fig. 8 Distribution of expansion lengths assessed from molecular combing data.

Supplementary Fig. 9 Somatic mosaicism detected by RP-PCR assays.

Supplementary Fig. 10 Micro-rearrangements observed in individuals with the largest expansions

Supplementary Fig. 11 Archimedes spiral drawings of patients 2-V-8, 2-IV-16 and 2-IV-9 compared to an unaffected individual.

Supplementary Fig. 12 Analysis of *MARCH6* expression in lymphoblastic cells and fibroblasts.

Supplementary Table 1 Expansion length characteristics calculated from molecular combing data.

Supplementary Table 2 Genotype-phenotype correlations.

Supplementary Fig. 1 Confirmation of TTTTA and TTTCA expansions in Families 1 (FAME3) and 5 (using exSTRa, STRetch and TRhist.

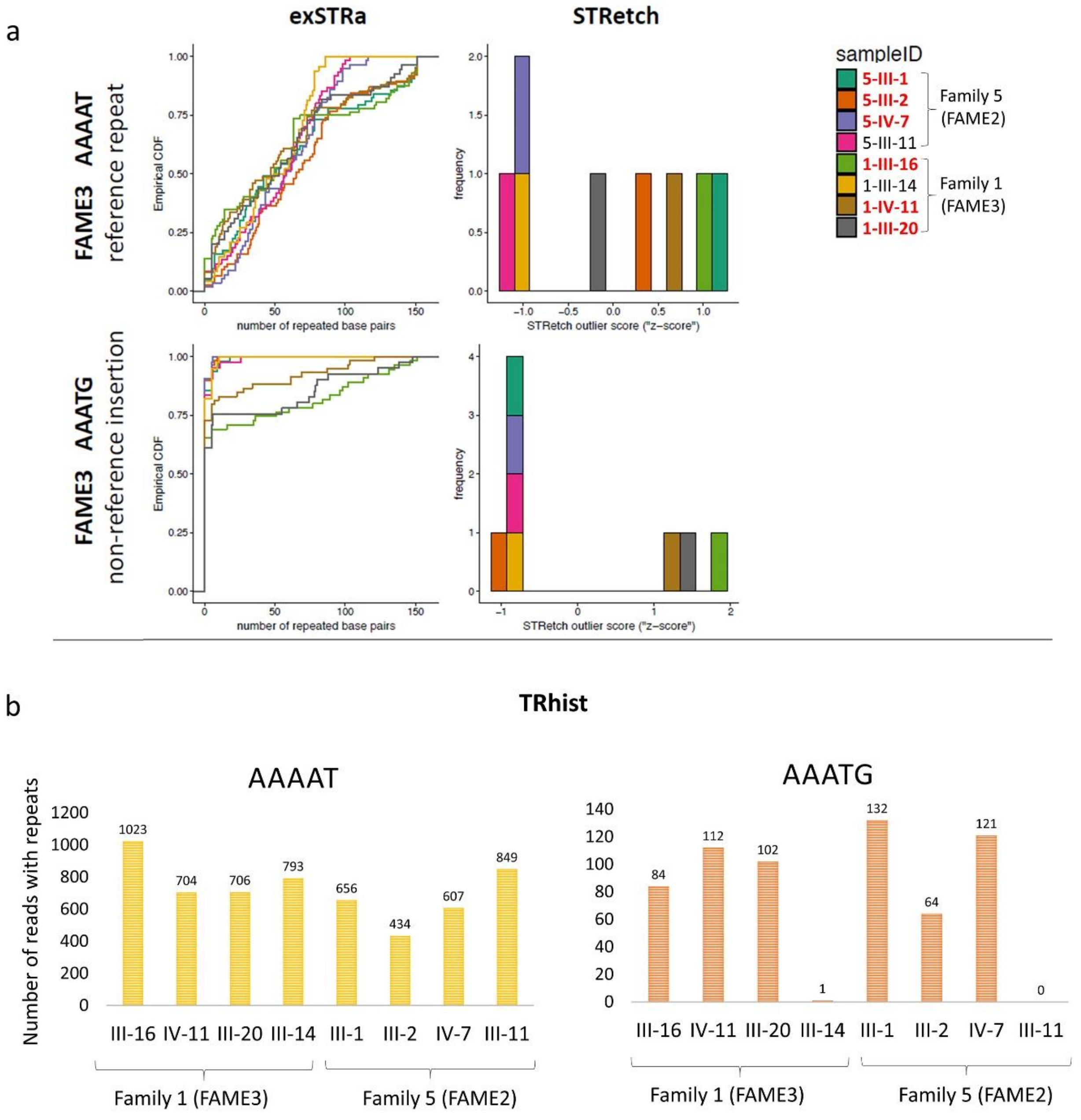

a) Detection of outliers (individuals with likely expansions) of AAAAT (TTTTA, above) and AAATG (TTTCA, below) repeats at the FAME3 locus in three affected individuals and one healthy spouse sampled from Families 5 (FAME2) and 1 (FAME3) (total of 8 individuals tested) using exSTRa and STRetch. Individuals 1-III-16, 1-III-20 and 1-III-14 are detected as outliers (likely to have repeat expansions), whilst other individuals are not (including the FAME2 family). Affected individuals are indicated in red. **b**) Number of reads with TTTTA or TTTCA repeats detected by TRhist. The graphs show the number of reads for which AAAAT (TTTTA, left) or AAATG (TTTCA, right) instances represent between 75 and 151 nucleotides. As previously observed using ExpansionHunter, only the number of AAATG repeats is discriminative between control and FAME individuals. However, since TRhist analyzes the genome-wide distribution of repeats, an increased number of TTTCA repeats is detected for both FAME2 (Family 5, chr 2) and FAME3 (Family 2, chr 5).

Supplementary Fig. 2 Pedigrees of FAME Families 5-13**,** tested negative for TTTTA/TTTCA expansion in *MARCH6*.

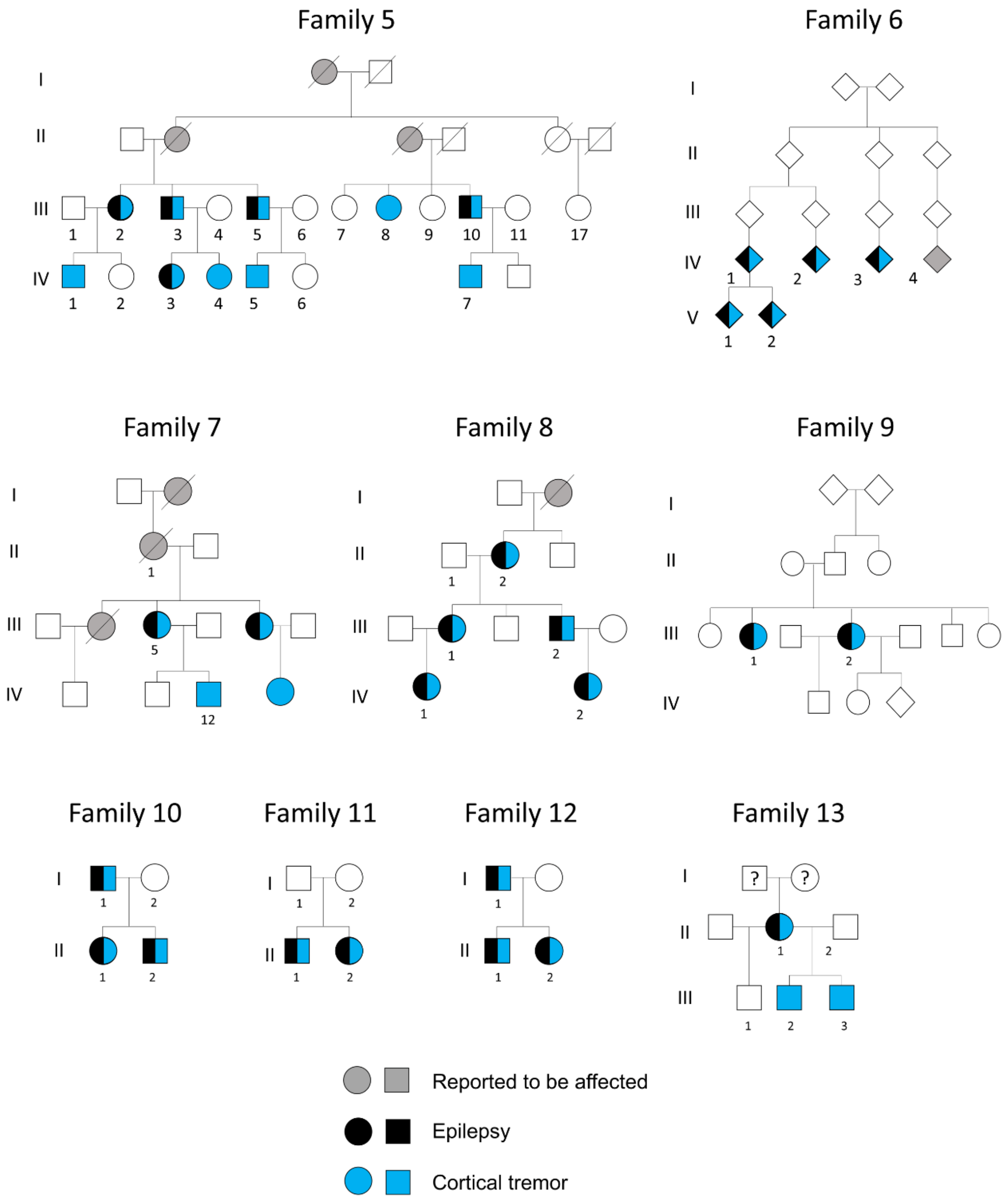

Families 5-7 have a TTTTA/TTTCA expansion on chr 2 (FAME2). Families 8-13 are also tested negative for FAME2.[22]

Supplementary Fig. 3. RP-PCR results corresponding to expansion carriers of Families 1-4.

a

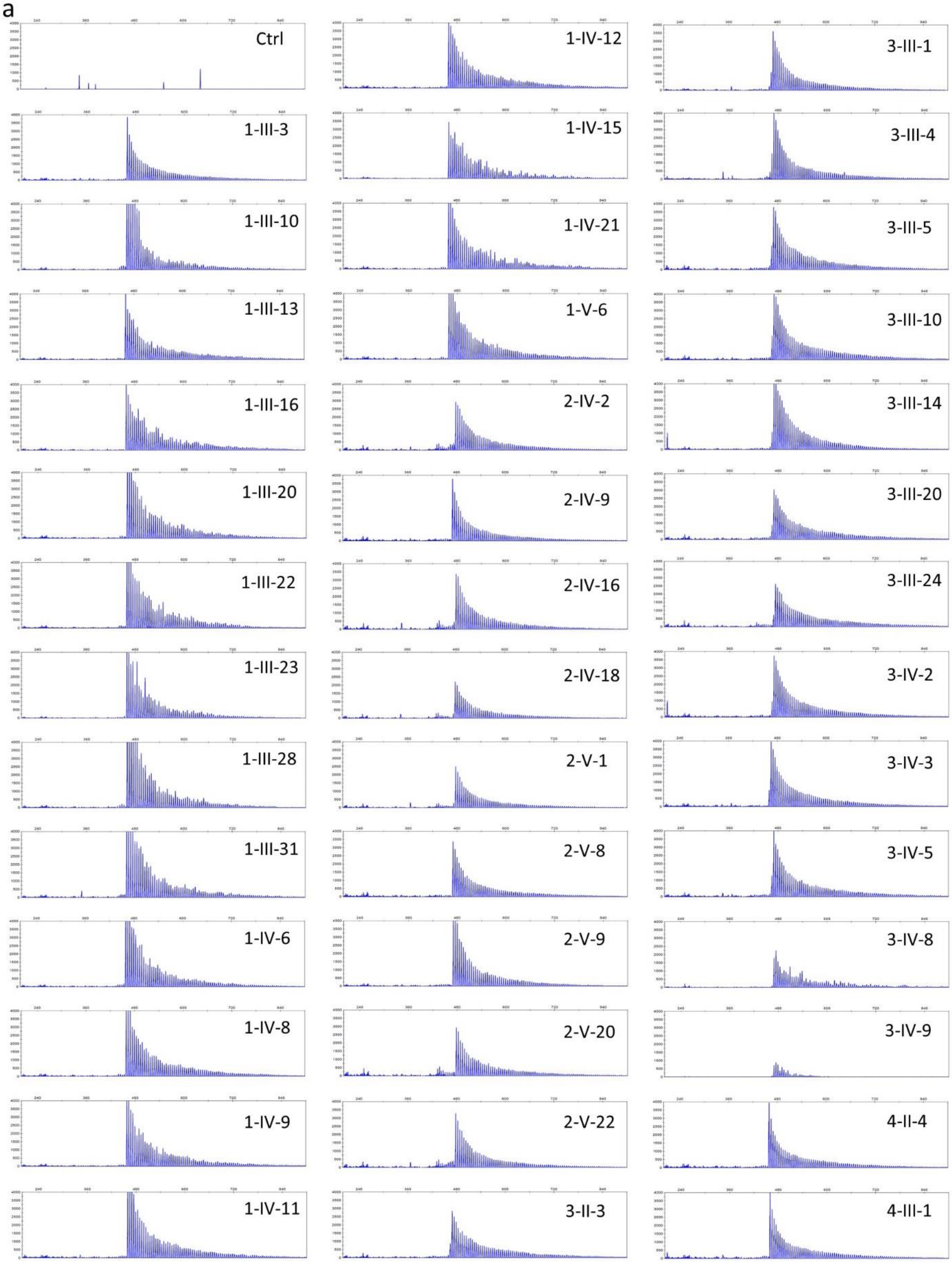

a) Results of 3'-TTTCA RP-PCR assay.

b

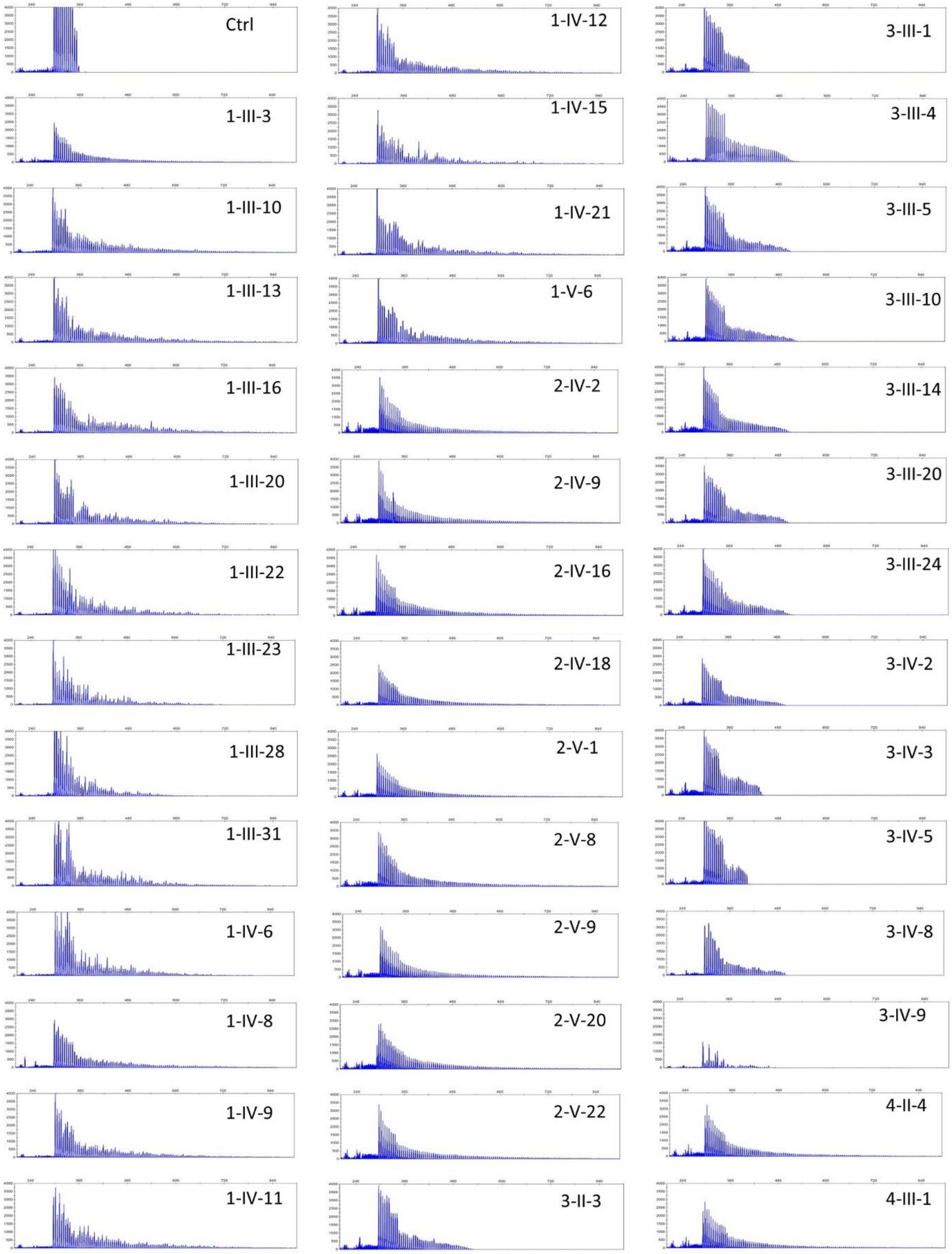

b) Results of 5’-AAAAT RP-PCR assay.

Supplementary Fig. 4. Analysis of the microsatellite region, where the MARCH6 expansion occurs, in healthy control individuals and during evolution.

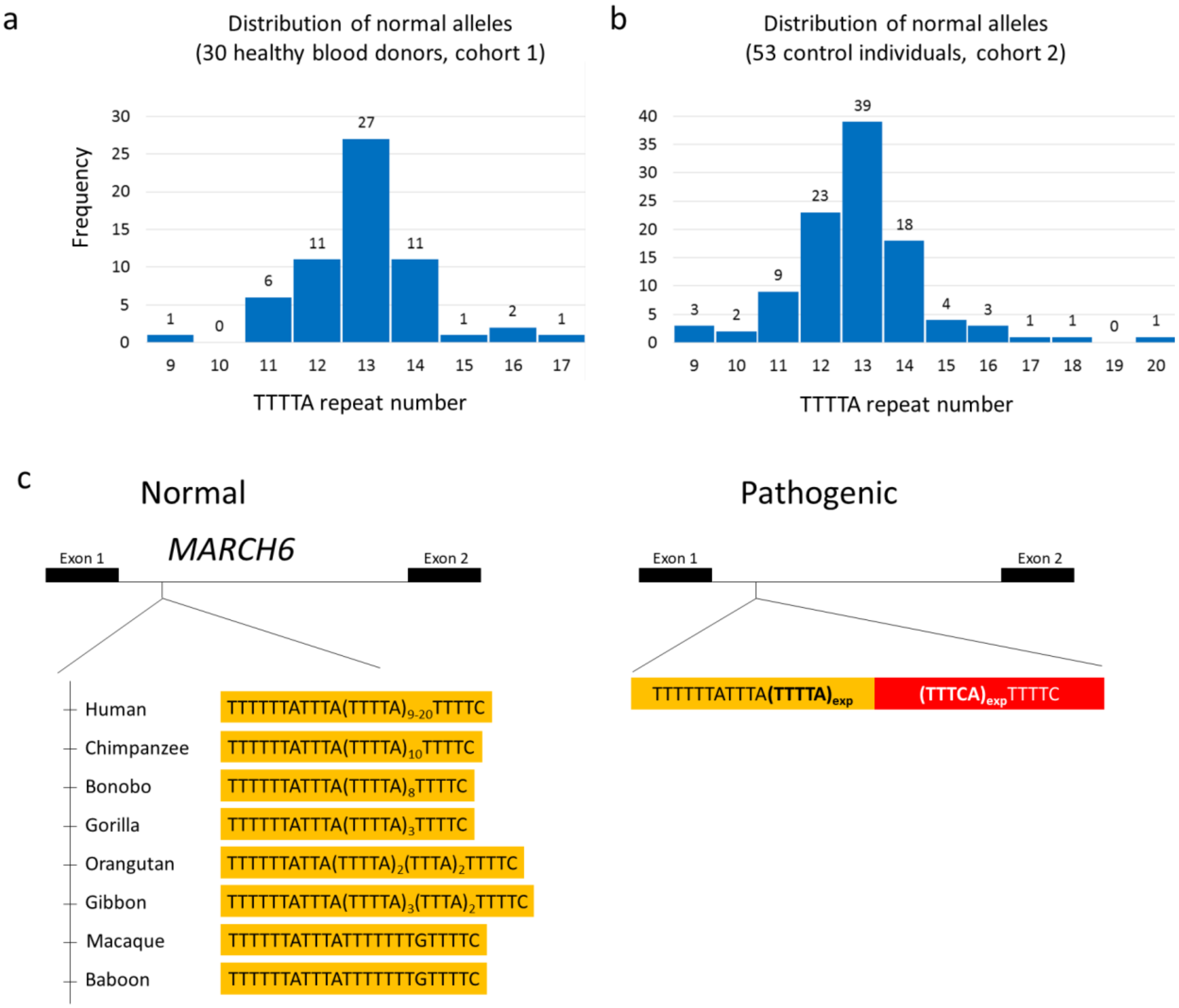

a) Distribution of *MARCH6* microsatellite alleles (number of TTTTA repeats) assessed by Sanger sequencing in 30 healthy blood donors (cohort 1). **b)** Distribution of *MARCH6* microsatellite alleles (number of TTTTA repeats) calculated by ExpansionHunter from genome data of 53 control individuals (cohort 2). **c)** Illustration of the number of TTTTA alleles present in humans and in reference genomes of primates and comparison with pathogenic alleles associated with FAME3.

Supplementary Fig. 5. Haplotyping of chr5 repeat expansion.

Name	Chr	Position	Family 1		Family 2		Shared haplotype
			1-IV-20	1-V-11	2-IV-9	2-IV-16	
rs13168213	5	10289503	TT	CT	CT	CT	
rs3857348	5	10297847	GG	AG	AA	AA	
rs13359758	5	10301295	TT	CT	TT	CT	**T**
rs2290670	5	10307698	GG	GG	AG	AG	**G**
rs606490	5	10318455	GG	GG	GG	GG	**G**
MARCH6 expansion							
rs2011318	5	10446124	GG	GG	GG	GG	**G**
rs11745100	5	10453133	GG	GG	GG	GG	**G**
rs1814856	5	10459942	TT	TT	TT	TT	**T**
rs814597	5	10468929	CC	CC	CC	CC	**C**
rs3932191	5	10470025	AG	AG	AG	GG	**G**
rs2962330	5	10481725	GG	GG	GG	GG	**G**
rs2589661	5	10492095	CT	CT	CC	CC	**C**
rs13180425	5	10492322	CC	CC	CT	TT	
rs6892802	5	10496509	TT	TT	TT	TT	

Common haplotype highlighted in blue. Yellow region refers to the expansion locus. The core haplotype from these two families is located at chr5:10301295-10492095, and is 190.8 kb (0.35 cM) in size. It contains the whole of *MARCH6*, and two other genes.

Supplementary Fig. 6. Visualization of extracted nanopore reads covering the expansion.

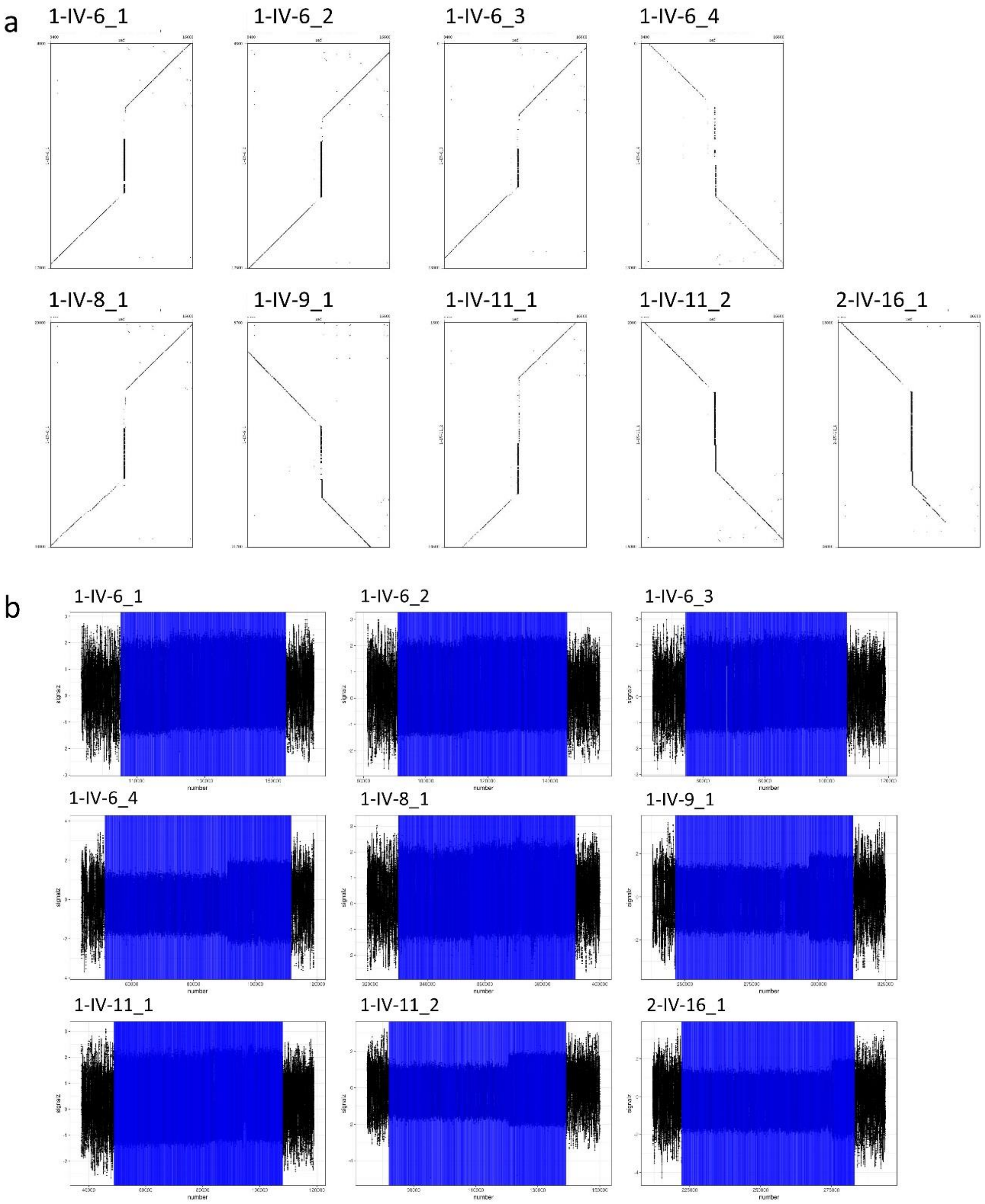

a) Dot plots comparing nanopore reads displaying the expansion (Y-axis, scale: 13 kb) with the corresponding hg19 reference region (X-axis, scale: 8.1 kb). The expansion appears as a vertical line. **b**) Analysis of raw nanopore reads using NanoSatellite. The signals corresponding to the expanded repeats appear in blue.

Supplementary Fig. 7. Distribution and interpretation of signals detected in blood samples by molecular combing.

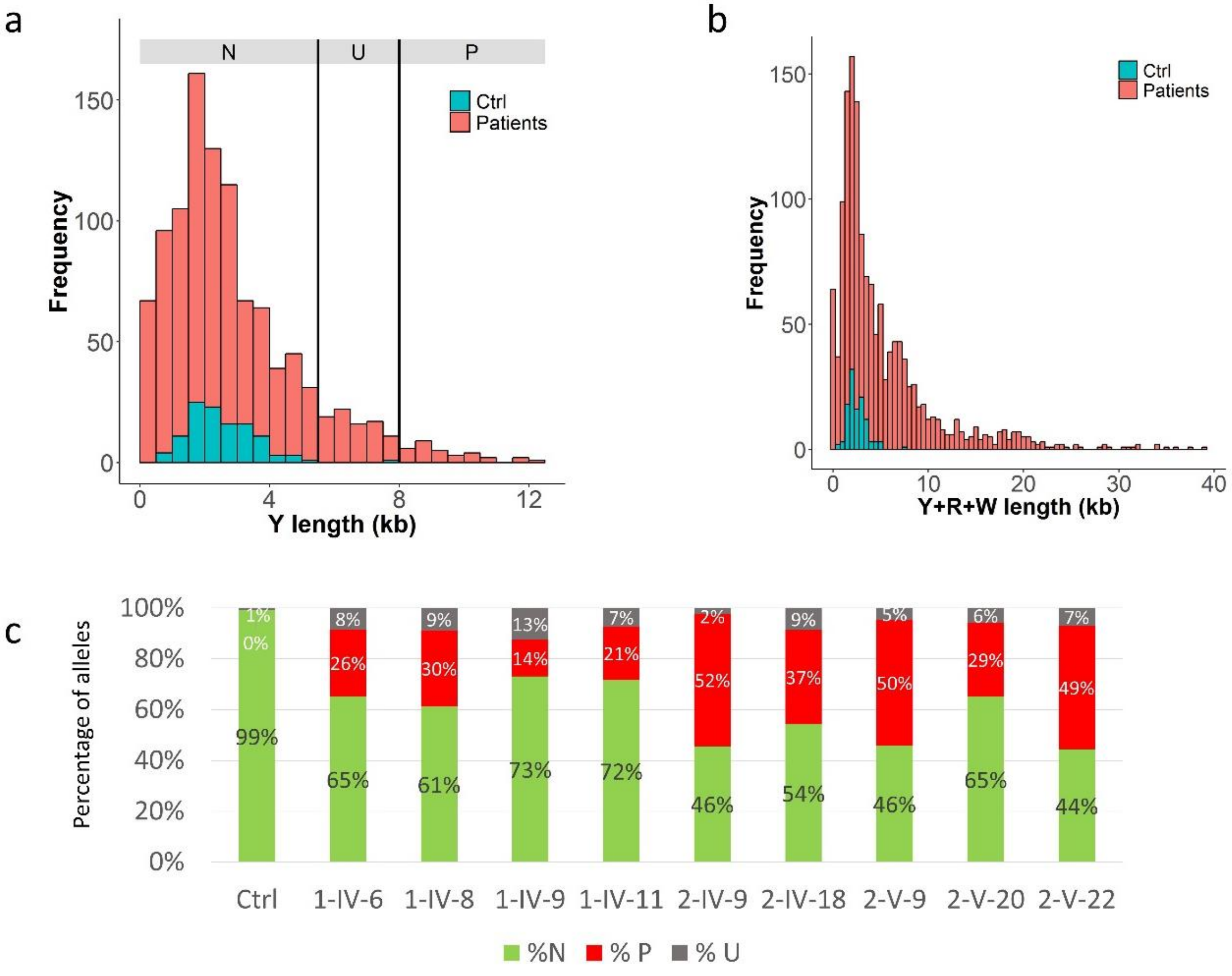

a) Distribution of the length of the unstained part between the blue (B) and green (G) signals (Y) for alleles without red (R) staining in a control individual and in patients. Alleles with Y < 5.5 were interpreted as normal (N); alleles with 5.5 ≤ Y < 8.5 were interpreted as undefined (U); alleles with Y ≥ 8.5 were interpreted as likely pathogenic (P) based on the distribution of Y observed in the control individuals. **b**) Distribution of the length of the unstained part between the blue and green signals (Y+R+W) for all alleles in a control individuals and in patients. **c)** Percentage of normal (N), undefined (U) and pathogenic (P) alleles in the control individual (Ctrl) and the nine patients analyzed by molecular combing. P includes both likely pathogenic alleles (no red staining but Y ≥ 8.5) and definite pathogenic (with red staining) alleles. This graphs shows that for the individuals with the smallest expansions, the percentage of pathogenic alleles is lower than expected by chance, which suggests a higher overlap between normal and pathogenic alleles in these individuals.

Supplementary Fig. 8. Distribution of expansion lengths assessed from molecular combing data.

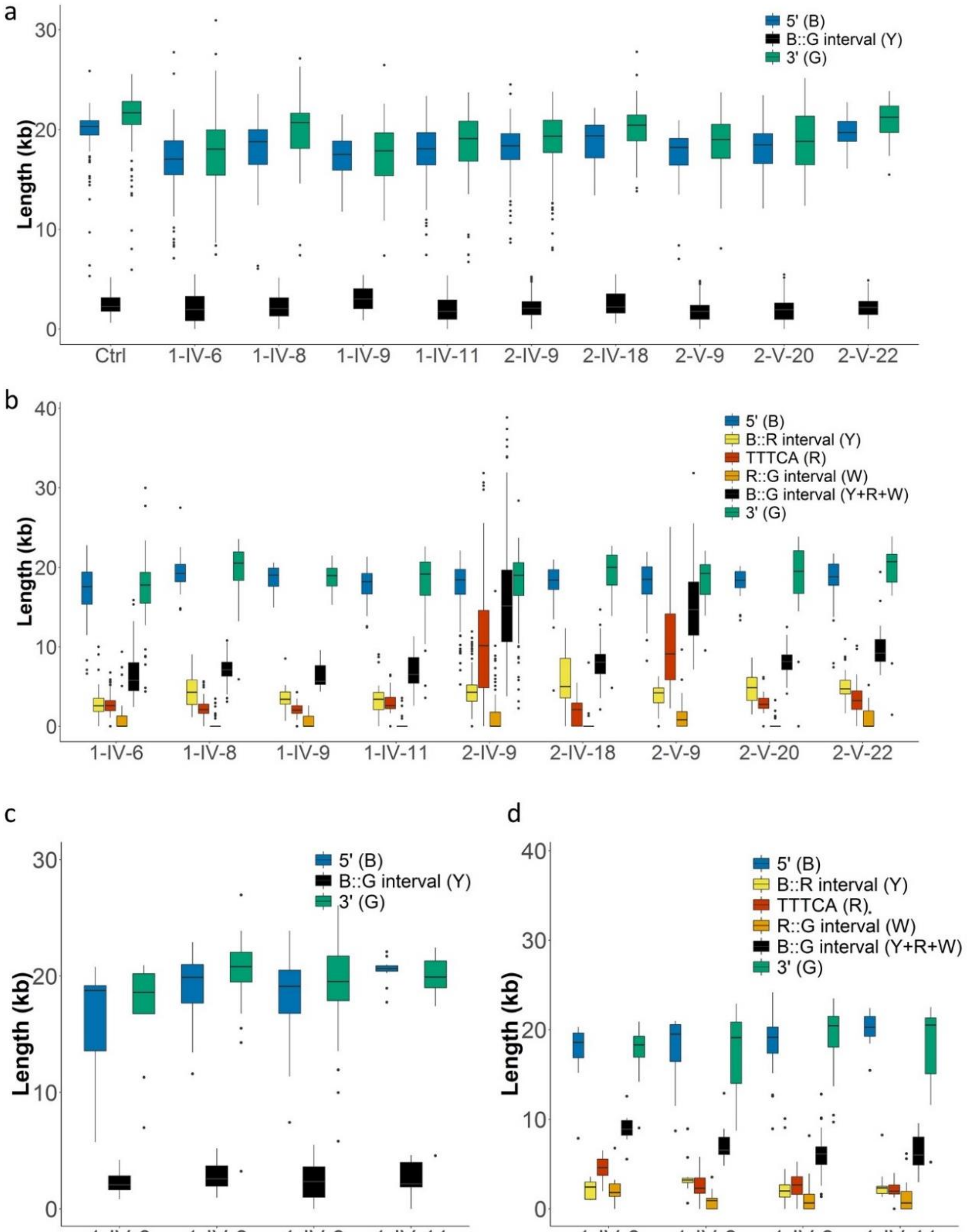

a) Box plots showing the size distributions of each part of the signals (B: blue, G: green) and the interval between them (Y) for alleles interpreted as normal (N) in blood samples. **b**) Box plots showing the distribution of each part of the signals for alleles interpreted as pathogenic (P) in blood samples. B (blue): 5' flanking region; R (red): TTTCA; G (green): 3' flanking region; Y (yellow): distance from B to R, interpreted as 5'-TTTTA; W (orange): distance from R to G, interpreted as 3'-TTTTA; Y+R+W (black): distance from B to G. Data corresponding to fibroblast samples are shown in **c)** for normal and **d**) for pathogenic alleles. Box plots elements are defined as follows: center line: median; box limits: upper and lower quartiles; whiskers: 1.5X interquartile range; points: outliers.

Supplementary Fig. 9. Somatic mosaicism detected by RP-PCR assays.

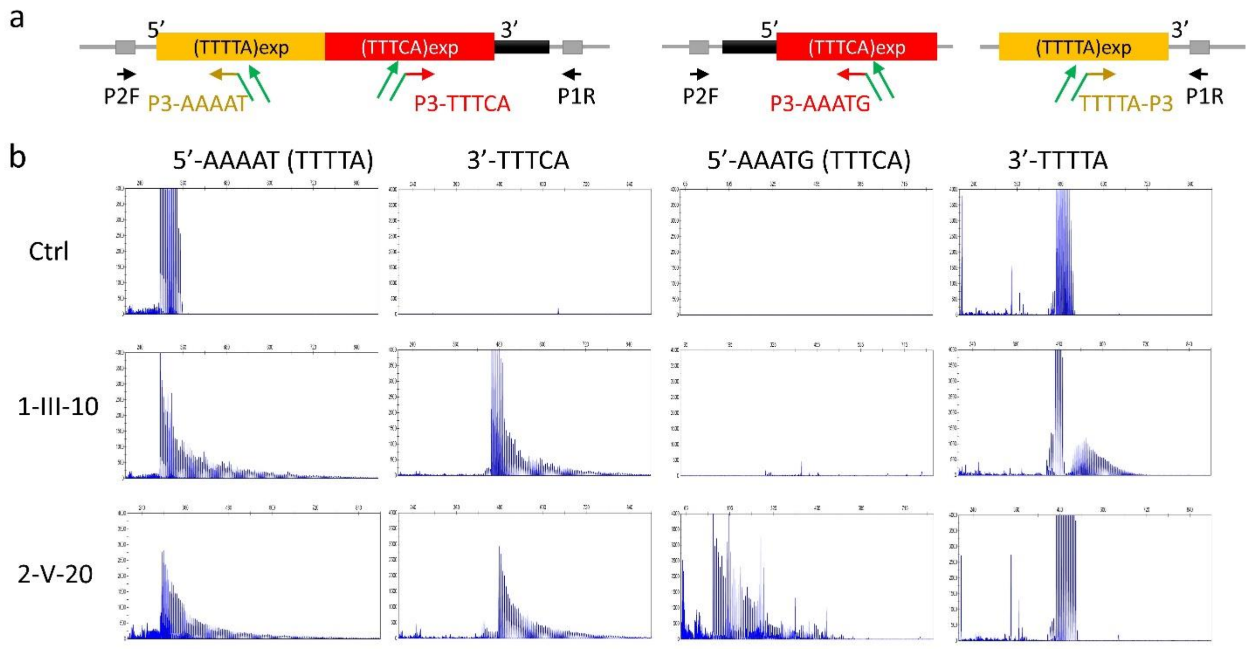

a) Principle of the four different RP-PCR assays that were developed and used to test the presence of TTTTA and TTTCA motifs at the 5' and 3' ends of the expansion. **b)** RP-PCR profiles of all four assays showing positive results for 3'-TTTTA (compatible with configurations C1 to C5, Fig. 4d) and 3'-TTTCA (compatible with configurations C6, Fig. 4d) assays in individual 1-III-10 and positive results for 5'-TTTCA (compatible with configurations C1, C2, C4 or C6, Fig. 4d) and 5'TTTTA assays (compatible with configurations C3 or C5, Fig. 4d) in individual 2-V-20, further demonstrating the existence of several expansion configurations as a result of somatic instability in these individuals.

Supplementary Fig. 10. Micro-rearrangements observed in individuals with the largest expansions.

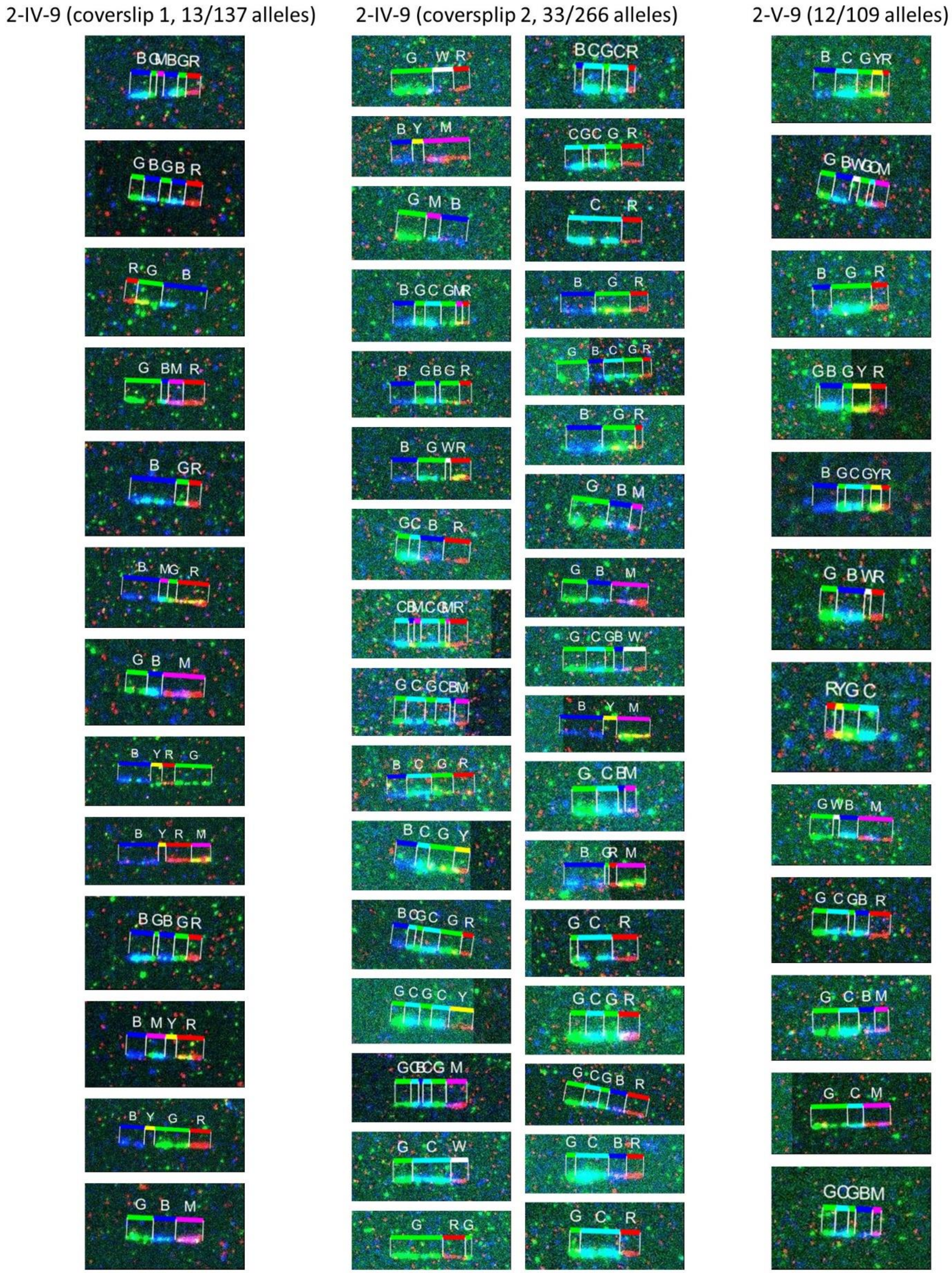

2-IV-9: 46/403 alleles counted on two different coverslips; 2-V-9: 12/109 alleles counted on 1 coverslip.

Supplementary Fig. 11. Archimedes spiral drawings of patients 2-V-8, 2-IV-16 and 2-IV-9 compared to an unaffected individual.

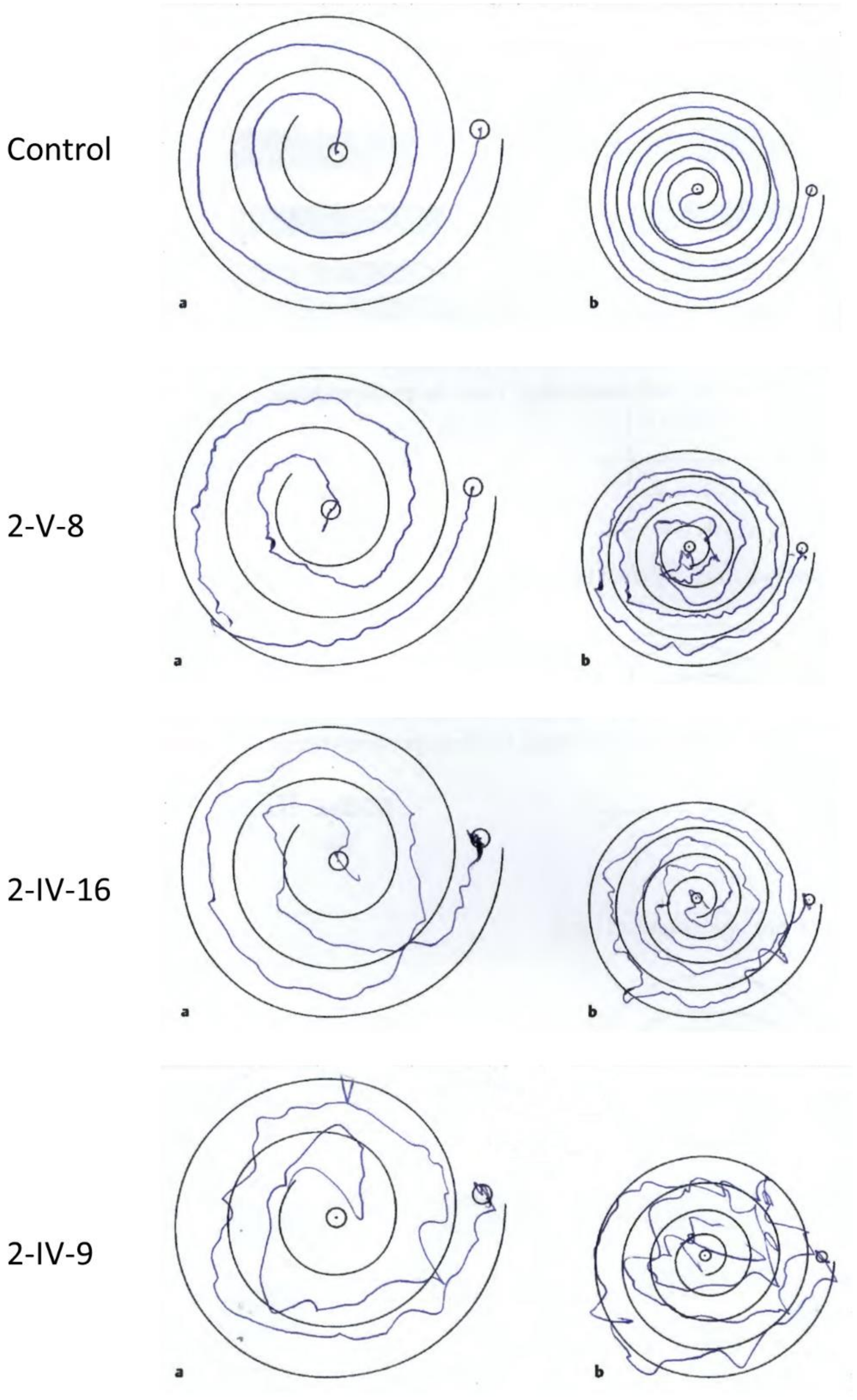

Supplementary Fig. 12. Analysis of *MARCH6* expression in lymphoblastic cells and fibroblasts.

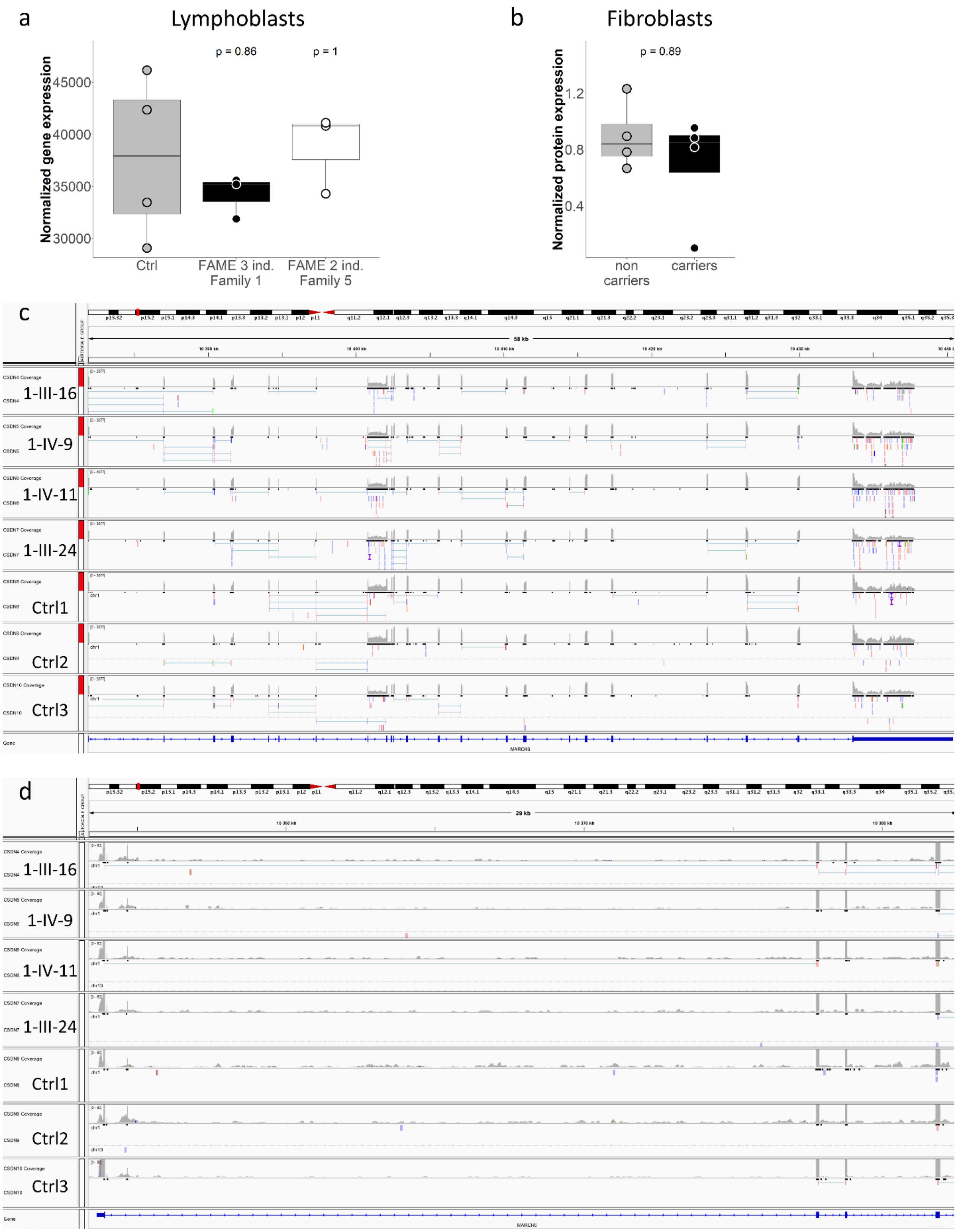

a) Box plots showing normalized *MARCH6* expression in lymphoblasts from four control individuals, three *MARCH6* expansion carriers from Family 1 and three affected individuals with an expansion at the FAME2 locus (Family 5). Box plots elements are defined as follows: center line: median; box limits: upper and lower quartiles; whiskers: 1.5X interquartile range; all values are displayed as points; outliers are shown as disconnected pointsNo statistical difference exists between cases and controls (Wald test). **b**) Quantification of the MARCH6 protein by Western blotting in fibroblasts of four expansion carrier individuals versus four unrelated control individuals. **c**) Visualization of RNAseq reads aligning to the entire *MARCH6* gene in Integrative Genomics Viewer (IGV). No difference was observed between members of Family 1 (1-III-16, 1-IV-9 and 1-IV-11) carrying a *MARCH6* expansion and control individuals (1-III-24 is a heathy spouse and Ctrl1-3 are healthy control individuals). **d**) Zoom of RNAseq reads aligning to the 5' end of *MARCH6*, including intron 1 where the expansion occurs.

Supplementary Table 1 Expansion length characteristics calculated from molecular combing data.

Individual	Tissue	region	mean	median	max	min	IQR	sd	mad
1-IV-6	blood	5'-TTTTA (Yp-Yn)	0.88	0.61	8.04	-1.96	1.70	1.88	1.42
1-IV-6	blood	TTTCA (R)	2.82	2.58	6.76	0.00	1.27	1.30	0.95
1-IV-6	blood	3'-TTTTA (W)	0.92	0.00	9.36	0.00	1.29	1.96	0.00
1-IV-6	blood	**Expansion (Yp-Yn+R+W)**	**4.62**	**3.82**	**13.91**	**0.47**	**3.53**	**3.21**	**2.30**
1-IV-8	blood	5'-TTTTA (Yp-Yn)	2.33	2.23	6.98	-0.92	3.09	2.05	2.28
1-IV-8	blood	TTTCA (R)	2.28	2.09	5.62	0.00	1.17	1.32	0.86
1-IV-8	blood	3'-TTTTA (W)	0.46	0.00	3.47	0.00	0.00	0.94	0.00
1-IV-8	blood	**Expansion (Yp-Yn+R+W)**	**5.06**	**5.05**	**8.73**	**1.03**	**1.76**	**1.75**	**1.41**
1-IV-9	blood	5'-TTTTA (Yp-Yn)	0.57	0.38	5.51	-2.34	1.53	1.81	1.17
1-IV-9	blood	TTTCA (R)	2.10	2.03	4.28	0.00	0.99	1.05	0.86
1-IV-9	blood	3'-TTTTA (W)	0.67	0.00	2.60	0.00	1.26	0.97	0.00
1-IV-9	blood	**Expansion (Yp-Yn+R+W)**	**3.34**	**2.65**	**6.59**	**1.34**	**2.38**	**1.67**	**1.47**
1-IV-11	blood	5'-TTTTA (Yp-Yn)	1.66	1.62	7.27	-1.77	2.17	2.17	1.70
1-IV-11	blood	TTTCA (R)	2.86	2.59	6.47	0.00	1.43	1.52	0.97
1-IV-11	blood	3'-TTTTA (W)	0.40	0.00	3.55	0.00	0.00	0.96	0.00
1-IV-11	blood	**Expansion (Yp-Yn+R+W)**	**4.92**	**4.74**	**9.52**	**0.81**	**3.24**	**2.05**	**1.99**
2-IV-9	blood	5'-TTTTA (Yp-Yn)	2.37	2.19	9.82	-2.09	2.05	2.33	1.66
2-IV-9	blood	TTTCA (R)	10.37	10.12	31.85	0.00	9.74	6.79	7.56
2-IV-9	blood	3'-TTTTA (W)	1.32	0.00	16.99	0.00	1.77	2.34	0.00
2-IV-9	blood	**Expansion (Yp-Yn+R+W)**	**14.07**	**13.05**	**36.77**	**1.68**	**9.02**	**7.19**	**6.69**
2-IV-18	blood	5'-TTTTA (Yp-Yn)	3.47	2.76	10.12	-2.22	4.96	3.02	3.07
2-IV-18	blood	TTTCA (R)	1.99	2.08	5.49	0.00	2.92	1.60	1.72
2-IV-18	blood	3'-TTTTA (W)	0.27	0.00	7.99	0.00	0.00	1.28	0.00
2-IV-18	blood	**Expansion (Yp-Yn+R+W)**	**5.72**	**5.84**	**12.45**	**-0.11**	**2.46**	**2.50**	**1.93**
2-V-9	blood	5'-TTTTA (Yp-Yn)	1.99	2.43	4.50	-1.78	1.87	1.57	1.45
2-V-9	blood	TTTCA (R)	10.04	9.09	25.09	2.24	8.30	5.11	5.74
2-V-9	blood	3'-TTTTA (W)	1.31	0.80	9.64	0.00	1.82	2.07	1.19
2-V-9	blood	**Expansion (Yp-Yn+R+W)**	**13.33**	**12.90**	**30.07**	**5.37**	**6.69**	**5.60**	**5.16**
2-V-20	blood	5'-TTTTA (Yp-Yn)	2.81	2.91	6.72	-0.45	2.89	2.06	2.11
2-V-20	blood	TTTCA (R)	2.93	2.75	6.18	0.00	1.28	1.53	1.16
2-V-20	blood	3'-TTTTA (W)	0.41	0.00	3.28	0.00	0.00	0.86	0.00
2-V-20	blood	**Expansion (Yp-Yn+R+W)**	**6.16**	**6.19**	**10.55**	**2.16**	**1.85**	**2.08**	**1.50**
2-V-22	blood	5'-TTTTA (Yp-Yn)	3.04	2.54	8.82	-0.54	1.71	1.97	1.42
2-V-22	blood	TTTCA (R)	3.60	3.23	10.13	0.00	2.35	2.28	1.68
2-V-22	blood	3'-TTTTA (W)	0.90	0.00	5.18	0.00	1.94	1.44	0.00
2-V-22	blood	**Expansion (Yp-Yn+R+W)**	**7.55**	**7.02**	**17.26**	**4.27**	**2.75**	**2.70**	**1.89**
1-IV-6	fibros	5'-TTTTA (Yp-Yn)	0.13	0.37	1.53	-1.08	1.93	1.04	1.48
1-IV-6	fibros	TTTCA (R)	4.56	4.61	6.53	1.97	1.81	1.41	1.62
1-IV-6	fibros	3'-TTTTA (W)	2.24	1.81	6.77	0.00	1.41	1.87	1.27
1-IV-6	fibros	**Expansion (Yp-Yn+R+W)**	**6.93**	**6.84**	**10.53**	**3.50**	**1.65**	**1.82**	**1.43**
1-IV-8	fibros	5'-TTTTA (Yp-Yn)	1.04	0.63	6.36	-1.94	0.50	2.12	0.46
1-IV-8	fibros	TTTCA (R)	2.63	2.28	5.80	0.00	1.71	1.68	0.90
1-IV-8	fibros	3'-TTTTA (W)	0.97	0.93	3.55	0.00	1.19	1.11	1.38
1-IV-8	fibros	**Expansion (Yp-Yn+R+W)**	**4.65**	**3.96**	**10.32**	**2.23**	**1.92**	**2.28**	**1.96**
1-IV-9	fibros	5'-TTTTA (Yp-Yn)	0.02	-0.35	7.72	-2.34	1.37	1.98	1.02
1-IV-9	fibros	TTTCA (R)	2.73	2.66	5.25	0.00	1.97	1.27	1.53
1-IV-9	fibros	3'-TTTTA (W)	1.08	0.66	8.17	0.00	1.64	1.54	0.98
1-IV-9	fibros	**Expansion (Yp-Yn+R+W)**	**3.82**	**3.79**	**10.46**	**-0.98**	**1.98**	**2.25**	**1.38**
1-IV-11	fibros	5'-TTTTA (Yp-Yn)	0.50	0.18	6.08	-0.83	0.80	1.78	0.55
1-IV-11	fibros	TTTCA (R)	2.04	1.96	4.00	0.00	1.00	0.95	0.47
1-IV-11	fibros	3'-TTTTA (W)	1.59	0.66	6.16	0.00	1.94	2.14	0.98
1-IV-11	fibros	**Expansion (Yp-Yn+R+W)**	**4.12**	**3.86**	**7.42**	**0.82**	**3.12**	**2.17**	**2.48**

Supplementary Table 2 Genotype-phenotype correlations.

Correlation	p-value	R	95% CI		R^2
Expansion ~ age_onset_epilepsy	0.0536	-0.7471	-0.9600	0.0136	0.5582
TTTCA ~ age_onset_epilepsy	0.0403	-0.7759	-0.9651	-0.0550	0.6021
5-TTTTA ~ age_onset_epilepsy	0.7094	0.1738	-0.6665	0.8196	0.0302
5'+3'-TTTTA ~ age_onset_epilepsy	0.6382	-0.2183	-0.8342	0.6400	0.0477
Expansion ~ age_onset_tremor	0.5953	0.2458	-0.6225	0.8428	0.0604
TTTCA ~ age_onset_tremor	0.5727	0.2604	-0.6128	0.8473	0.0678
5-TTTTA ~ age_onset_tremor	0.9145	0.0504	-0.7304	0.7741	0.0025
5'+3'-TTTTA ~ age_onset_tremor	0.8032	0.1167	-0.6977	0.7995	0.0136

R is the Pearson's correlation coefficient; CI: confidence interval
