# Supplementary material for: Unstable TTTTA/TTTCA expansions in MARCH6 are associated with Familial Adult Myoclonic Epilepsy type 3
Source: Nat Commun. 2019 Oct 29;10:4919. doi: 10.1038/s41467-019-12763-9 (PMC6820781; doi:10.1038/s41467-019-12763-9)
Supplement: Supplementary file 4 — Supplementary Data 3 [file 41467_2019_12763_MOESM4_ESM.pdf]

Supplementary Data 3. Sequences obtained from extracted nanopore reads using guppy as Basecaller in six expansion carriers from Families 1 and 2.

a) 1-IV-6_1 (-)

GAAAACACTTATACTGTTGTGGTTCTGTTTTTTATTTATTTTATTTTTATTTTATTTTATTTTATTTTATTTTA
TTTTATTTTATTTTTATTTTATTTTATTTTATTTATTTTATTTTATTTTATTTTATTTTTATTTTATTT
TATTTTTATTTTATTTTATTTTATTTTATTTTATTTTATTTTATTTTATTTTATTTTACTTTATTTTGTTTTAT
TTTATTTTTATTTTATTTTATTTTATTTTTATTTTATTTTATTTTATTTTATTTTATTTTATTTTATTTTATTT
TATTTTATTTTATTTTATTTTATTTTATTTTATTTTATTTTTTATTTTATTTCTATTTTATTTTATTTTTATTT
TATTTTATTTATTTTTATTTTATTTATTATTTTATTTATTTTATTTTTATTTTATTTTATTTATTTTTATTTTA
TTTTTATTTTTATTTTATTTTGTTTTATTTTATTTTATTTATTTTATTTTATTTATTTTATTTTTATTTTATTT
TTATTTTATTTTATTTTATTTTATTTTATTTTTATTTATTTTATTTTATTTTATTTTATTTATTTTTATTTTAT
TTCTTATTTTATTTATTTTATTTTATTTTGGTTTTATTTTTATTTTATTTTATTTTTATTTTATTTTATTTTAT
TTTATTTTATTTTATTTTATTTTATTTTATTTTATTTTTATTTTATTTATTTTATTTTTATTTATTTTATTTTA
TTTTTATTTTATTTTTATTTTATTTTATTTATTTTATTTTTATTTTATTTTATTTTATTTTTATTTTTATTTTTA
TTTTATTTTTTATTTTATTTTTATTTTATTTATTTTATTTTATTTTTATTTTATTTATTTATTTATTTATTTTA
TTTTTATTTTATTTTATTTTATTTTTATTTTATTTTATTTTTATTTTTATTTTATTTTTATTTTATTTTATTTA
TTTTTATTTTATTTTATTTTATTTTATTTTATTTTATTTTATTTTATTTTATTTTATTTTATTTTTATTTTGGT
TTTATTTTATTTTATTTTATTTTATTTTATTTTATTTTATTTTTATTTTATTTATTTTTATTTTATTTCTTGTT
TTTATTTTATTTATTTTATTTTATTTTTATTTTATTTTATTTTATTTTTTTATTTATTTTTATTTTTATTTTAT
TTATTTTTGTTTTTATTTTTATTATTTTTATTTTTATTTTATTTTATTTTATTTTTTATTTATTTTATTTTTAT
TTGTTTATTTTATTTTATTTTATTTTATTTTTATTTTTATTTTATTTTATTTTATTTTATTTTTATTTTTATTT
TTATTTTATTTTTTATTTTATTTTATTTTATTTTATTTTATTTTATTTTATTTTATTTTTATTTTATTTTATTT
TATTTTTATTTTACTTTATTTTATTTTATTTTTATTTATTTTATTTTATTTATTTTTATTTTGTTTTATTTTAT
TTTTATTTTATTTTTATTTTATTTTATTTTATTTTATTTTATTTTATTTTATATTTTATTTTATTTTATTTTAT
TTTATTTTTATTTTATTTATTTTTTGGTTTTATTTTATTTTATTTTATTTTTATTTTATTTTATTTTTTATTTT
ATTTTTATTTTTATTTTATTTTATTTTTATTTTATTTTATTTTATTTTATTTTATTTTATTTTATTTTATTTT
ATTTCTTTTTTATTTTGTTTTATTTTATTTTATTTTTATTTTATTTTTATTTTATTTTATTTTATTTTATTTTT
ATTTTTTATTTTATTTTATTTTATTTTATTTATTTATTTTATTTTTATTTTATTTTATTTTATTTTATTTTATT
TTATTTATTTTATTTTATTTTTATTTTATTTTTATTTATTTTATTTTATTTTATTTTATTTTATTTTATTTTTA
TTTATTTTATTTATTTTATTTTTATTTTTATTTTTATTTTTATTTTATTTTATTTTATTTTATTTATTTTATTT
TTATTTTATTTTTATTTTTATTTTATTTTATTTTATTTTTTATTTTTAGTTTGTTTTATTTATTTTATTTTTA
TTTTATTTTATTTTTATTTTTATTTATTTATTTTATTTTATTTTATTTTGGTTTTATTTTATTTTATTTTATTT
TATTTTTATTTTATTTTATTTTATTTTATTTATTTTATTTTTATTTTATTTTATTTTATTTTATTTTATTTTTA
TTTTATTTTATTTTATTTTATTTTATTTTATTTTATTTATTTTATTTATTTATTTTACTTATTTATTTATTTTA
TTTCTATTTATTTCTATTTATTTATTTCATTTATTTATTTCTTATTTCTTATTTATTTCTATTTATTTATTTAT
TTTCTTATTTCTATTTATTTATTTTTATTTATTTTATTTCTATTTTATTTATTTATTTTATTTATTTCTTTATT
TATTTATTTTCTATTTGTTTTATTTTTATTTTTATTTCTTTATTTCTATTTTTATTTATTTATTTATTTATTTT
CTATTTCTTTATTTATTTTCATTTATTTATTTATTTCATTTCTATTTATTTATTTTTATTTATTTATTTCTTAT
TTCTTATTTCTATTATTTCTATTTCTATTTCTATTTCTATTTCTTATTTCTATTTATTTATATTTTATTTCTAT
TTATTTATTTATTTATTTCTTATTTATTTTTTATTTCTTTATTTATTTTATTTATTTATTTATTTCTATTTCTA
TTTCTATTTCTATTTCTTATTATTTATTTATTATTTCTTATTTATTTCATTTATTTCTTATTTATTTATTTATT
TTATTTATTTTCTTATTTATTTCTATTTCTATTTGGTTTTATTTATTTTCTATTTATTTCTTATTTCAGTTTTA
TTTTATTTATTTATTTATTTCTATTTATTTCTATTTCTTATTATTTATTGTTTATTTATTTATTTATTTATTTT
ATTTCTTATATTTTTTATTTCTATTTTCTTATTTCTTATTTCTTATTTATTTCTTATTTCTATTTTCATTTTAT
TTATTTATTTATTTATTTTTATTTATTTATTTCTATTTATTTATTTATTTCTATTTCTTATTTATTTATTTATT
TTATTTATTTATTTATTTCTTATTTATTTATTTATTTATTTATTTTTATTTTCTTATTTATTTCTATTTATTTA
TTTATTTCTTATTTATTTATTTATTTATTTATTTATTTATTTATTTATTATTTCTATTTCTATTTATTTATTTA
TTTATTTATTTATTTATTTATTTATTTATTTATTTATTTCTATTTTATTTCTATTTTTATTTATTTATTTATTT
TCTTATTTCTATTTCTATTTCTTATTTATTTCTATTTTATTTATTTTATTTTATTTCTTATTTTGCTTATTTCT
ATTTCTTATTTCTATTTCTATTTATTTATTTATTTATTTATTTATTTATTTATTTCTTATTTCTATTTATTTCT
ATTTATTTATTTATTTATTTATTTCTTATTTCTATTTCTTATTTCTTATTTATTTATTTCTTATTTATTTCTAT
TTCTATTTTATTTATTTATTTATTTATTTATTTATTTATTTCTATTTATTTATTTTTATTTATTTCTATTTATT
TCATTTATTTATTTCTATTTATTTCTATTTCTATTTATTTATTTCTATTTCTTATTTCTTATTTCATTTTATTT

CATTTCTATTTCTTATTTATTTATTTCTTATTTCTTATTTCTATTTATTTCTATTTCTATTTCTTATTTATTTA
TTTCATTTCTATTTCTATTTCTATTTCTATTTATTTATTTTCTTATTTATTTCTTATTTCTATTTCATTTCTAT
TTATTTCTATTTATTTATTTTATTTATTTCTTATTTCTATTTCATTTCTTATTTATTTCTATTTCTTATTTCT
TATTTCATTTCATTTCATTTCATTTCTATTTCTTATTTATTTATTTATTTCTTTATTTCATTTCTATTTATTTA
TTTATTTATTTATTTATTTCATTTCTATTTCTATTTATTTATTTCATTTCTATTTCTATTTCATTTCTATTTCA
TTTCTATTTCTATTTCATTTATTTATTTCTATTTATTTCTTATTTCATTTCATTTATTCTCTATTTCATTTCTA
TTTATTTCTATTTCATTTCATTTCATTTCATTTCATTTTGCTTATTTCTATTTATTTCATTTCATTTCATTTCA
TTTCATTTATTTCTATTTCCTTTCATTTCATTTCTATTTCATTTCATTTTCCGAGATGGA

b) 1-IV-6_2 (-)
GAAAACACTTATACTGTTGTGGTTCTGTTTTTATTTATTTTATTTTATTTTATTTTATTTTATTTTTATTTTTT
ATTTTATTTTATTTTATTTTATTTTATTTTATTTTATTTTTATTTTATTTTATTTTTATTTTATTTTATTTTA
TTTTATTTTATTTTATTTTCTTATTTTTATTTTTATTTTATTTTATTTTATTTTATTTTATTTTTATTTTATTT
TATTTTATTTTTATTTTATTTTATTTTTATTTTTATTTTATTTTATTTTATTTTATTTTATTTTATTTTATTTT
ATTTTATTTTATTTTATTTTATTTTTATTTTTATTTTTATTTTTATTTTTATTTTTATTTATTTTATTTTATTT
TATTTTTATTTTATTTTATTTATTTTATTTTATTTTATTTTTATTTTATTTTATTTTTATTTTATTTTTATTTT
ATTTTTATTTTATTTTTATTTTATTTTATTTTATTTTATTTTATTTTATTTTATTTTATTTTATTTTATTTTAT
TTTATTTTATTTTATTTTATTCATTTTATCTTTATCTTTATCTTTATCTTTATCTTATTCTTATCTTTATCTTT
ATCTTTTATCTTTTTATATCTTTATCTTATCTTTATCTTTTATCTTTATCTTTATCTTATCTTATCTTATCTTA
TCTTTGACTTTATCTTTATCTTTATCTTTATCTTTACCTTATCTTTATCTTTATTTTATTCTTTATTTTATTTT
ATTTTATTTTATTTTATTTTTATTTTATTTTTATTTTATTTTTTATTTTATTTTATTTTATTTTTATTTTATTT
TATTTTATTTTATTTTATTTTATTTTATTTTATTTTATTTATTTTTTTATTTTATTTTATTTTTATTTTATTTT
ATTTTATTTTATTTTTATTTTATTTTATTTTATTTATTTTATTTTATTTTATTTTATTTTATTTTATTTTATTTT
ATTTTATTTATTTTATTTTATTTTATTTTATTTTATTTTATTTTTATTTTATTTATTTTTTATTTTATTTTATT
TTATTTTATTTTATTTTATTTTATTTTATTTTATTTTATTTTATTTTATTTTATTTTATTTTATTTTATTTTAT
TTATTTTTATTTTATTTTATTTTTATTTTATTTTATTTTATTTTTATTTTATTTTATTTTATTTTTATTTTTAT
TTTATTTTATTTTATTTTATTTTATTTTTATTTTATTTTATTTATTTTATTTTATTTTATTTTATTTTATTTTA
TTTTTGCTTTATTTTATTTTATTATTTATTTTATTTTATTTTATTTATTTTATTTTATTTTATTTTATTTTATT
TTATTTTATTTTATTTTATTTTATTTTTATTTTATTTATTTTATTTTATTTTTATTTATTTTATTTATTTATTT
TATTTTATTTTATTTTATTTTTATTTTATTTTATTTTATTTTAGTTTTATTTTATTTTATTTTTATTTTATTTT
TATTTTATTTTATTTATTTTATTTTATTTTATTTTATTTTATTTTATTTTATTTTTATTTTATTTATTTTATTT
TTATTTTATTTTATTTTTATTTTTATTTTATTTTATTTTTATTTTATTTTATTTTATTTTTATTTTATTTATTT
TATTTTATTTTATTTTATTTTATTTTTATTTTATTTTATTTATTTTATTTTATTTTTATTTTATTTTATTTTA
TTTTATTTTATTTTATTTTATTTATTTTATTTTATTTTATTTTATTTTATTTTATTTTATTTTATTTTATTTTA
TTTTATTTTATTTTATTTTTATTTTTATTTTATTTTTATTTTATTTTATTTTATTTATTTTATTTTATTTTAT
TTATTTTATTTATTTTATTTATTTTATTTTATTTTATTTTATTTTATTTTTATTTTTATTTTATTTTATTTTAT
TTTATTTATTTTTATTTTATTTTATTTTATTTTATTTTATTTTTATTTTATTTTTATTTTATTTTATTTTATTT
TATTTTATTTTATTTTATTTTATTTTATTTATTTTATTTTTATTTTATTTATTTTATTTTATTTTATTTTTATT
TTATTTTATTTTATTTTATTTTATTTTATTTTATTTTTATTTTATTTTATTTTATTTTATTTTATTTTATTTTA
TTTTATTTTATTTTATTTTATTTTATTTTATTTTATTTTATTTTATTTTATTTTATTTTATTTTATTTTATTTT
ATTTATTTTATTTTATTTTATTTTATTTTATTTTATTTTATTTATTTTATTTTATTTTTATTTTATTATTTTAT
TTTATTTTTATTTTATTTTCTTATTTTATTTTATTTTATTTTATTTTATTTTATTTTATTTTATTTTATTTTAT
TTTATTTTTATTTTATTTTATTTATTTTATTTTTATTTTTATTTTATTTTATTTTATTTTATTTTATTTTATTT
TATTTTATTTTTATTTTATTTTATTTTATTTTATTTTATTTTATTTTATTTTATTTTATTTTATTTTATTTTAT
TTTATTTATTTTATTTTATTTTATTTTATTTTATTTTATTTTATTTTATTTTTTTATTTTATTTTATTTTATTTT
TATTTTATTTTATTTTTATTTATTTTTATTTTATTTTATTTTATTTTTATTTTATTTTATTTTATTTTTATTTT
ATTTTTATTTTATTTATTTATTTTATTTTATTTTATTTATTTTATTTTATTTTATTTTATTTTATTTATTTTAT
TTTATTTTATTTTATTTTATTTTATTTTATTTTTATTTTATTTTATTTTATTTTATTTTATTTTATTTTATTTT
ATTTTATTTTATTTTATTTTATTTATTTTATTTTTATTTTATTTATTTTATTTTATTTTATTTTATTTTATTTT
ATTTTATTTATTTTATTTTATTTTATTTTATTTTTATTTTATTTATTTTATTTTATTATTTTATTTTATTTTA
TTTTATTTTATTTTATTTTATTTTATTTTATTTTATTTTATTTTATTTTATTTTATTTTATTTTATTTTATTTT
ATTTTATTTTATTTTATTTTATTTTTATTTTATTTTATTTTATTTTATTTTATTTTATTTTATTTTATTTTATT
TTATTTTATTTTATTTTATTTTTATTTTATTTTATTTATTTATTTTATTTCTATTTTATTTATTTATTTATTTC
TATTTCTATTTATTTCTATTTCTATTTATTTATTTATTTCTTATTTATTTATTTATTTATTTATTTCTATTTAT

TTCTATTTATTTATTTCTTATTTCTATTTATTTCTATTTATTTCTTATTTCTATTTATTTATTTCTATTTCTAT
TTCATTTATTTTATTTCTATTTATTTATTTATTTCTATTTCTATTTCTATTTATTTATTTATTTATTTCTATTT
TCTATTTCTATTTCTATTTCTATTTCTATTTATTTATTTATTTCTATTTCTTATTTCTATTTCTATTTCTTATT
TCATTTATTTCTTATTTCTATTTCTTATTTATTTATTTCTATTTTTATTTATTTCTATTTTTATTTATTTTATT
TCTATTTCTATTTCTTATTTCTATTTTTATTTATTTATTTCTATTTCTATTTATTTATTTCTATTTATTTCTAT
TTCTATTTCATTTTATTTATTTCTATTTCTTATTTATTTCTATTTCTTATTTATTTCTATTTTTATTTATTTAT
TTCTATTTATTTCTATTTATTTATTTCTTATTTCTATTTCTATTTCTATTTCTATTTATTTCTATTTATTTCTT
ATTTATTTCTATTTATTTATTTCTATTTATTTCTATTTCTTATTTATTTATTTATTTCTATTTATTTCTATTTA
TTTCTATTTATTTATTTCTATTTCTATTTCTTATTTACTTATTTCTTATTTATTTATTTATTTATTTATTTAT
TTCTATTTCTATTTATTTATTTCTATTTATTTCTATTTATTTCTATTTCTTATTTCTATTTATTTCATTTATTT
ATTTCTTATTTATTTCTATTTATTTATTTCTATTTATTTATTTATTTCTATTTCTATTTCTATTTCTATTTCTA
TTTCTTATTTCTATTTCATTTATTTATTTATTTATTTATTTATTTATTTCTATTTATTTATTTATTTCTATTTA
TTTCTTATTTCTTATTTATTTATTTCTATTTATTTCTATTTATTTCTATTTATTTATTTCTATTTATTTATTTT
ATTTATTTCTATTTCTTATTTCTTATTTCTATTTATTTCTATTTATTTATTTATTTATTTTCATTTATTTATTT
ATTTATTTATTTATTTATTTATTTATTTCTATTTATTTATTTATTTCTATTTATTTCTATTTATTTCTATTTCT
TATTTTATTTCTTATTTCTTATTTATTTCTATTTCTATTTATTTCTATTTTTATTTCTATTTATTTCTATTTCT
ATTTATTTCTATTTATTTCTATTTCTATTTATTTATTTCTTATTTCTATTTCTTATTTCTATTTATATTTCTAT
TTCTATTTATTTATTTCTTATTTCTATTTATTTATTTCTTATTTATTTCTATTTCTATTTATTTATTTATTTAT
TTCATTATTTCTTATTTCTTATTTCTATTTCATTTATTTCTATTTCTTATTTCTATTTCTTATTTATTTCTATA
TTTCATTTATTTCATTTCTATTTTATTTCATTTCATTTATTTATTTCATTTCATTTCATTTCATTTCATTTTAT
TTTATTTCTATTTCTATTTCTTATTTCTATTTCTATTTCTATTTCTCATTTCTATTTCATTTCATTTCATTTCA
TTTCTATTTATTTCATTTTCTATTTCATTTCTCATTTCATTTCATTTCCATTTCATTTCATTCTATTTTATTTT
CATTTCTATTTCTATTTCATTTCTATTTCATTTCATTTCCTCATTTATTTCTATTTCATTTCATTTCATTTCAT
TTCATTTCATTTCATTTCTATTTCTATTTCATTTTCTCTGAGATGGA

c) 1-IV-6_3 (-)
GAAAACACTTATACTGTTGTGGTTCTGTTTTTTATTTATTTTATTTTATTTTATTTTATTTTATTTTATTTTAT
TTTATTTTTATTTTATTTTATTTTATTTTTATTTATTTTATTTTATTTTATTTTATTTTATTTTATTTTATTTT
ATTTTATTTTATTTTATTTATTTTATTTTATTTTATTTTTATTTCTTTTATTTTATTTTATTTTATTTTATTTT
ATTTTATTTTATTTTATTTTATTTTATTTTTATTTTATTTTATTTTATTTTATTTTATTTTTATTTATTTTATTT
TTATTTTATTTTATTTTATTTTATTTTATTTTATTTTATTTTATTTTATTTTATTTTTATTTTATTTTATTTTAT
TTTATTTTATTTTATTTTATTTTATTTTATTTTATTTTATTTTATTTTATTTTATTTTATTTTATTTTATTTTA
TTTTATTTTATTTATTTTATTTTATTTTATTTTATTTTATTTATTTTTATTTTATTTATTTTTATTTTATTTT
ATTTTATTTTATTTATTTTATTTTTATTTTATTTTTATTTTATTTTATTTTATTTTATTTTATTTTATTTTATT
TTATTTTATTTTATTTTTATTTTATTTTATTTTATTTTTATTTTATTTTATTTTATTTTTATTTTATTTTATTT
TATTTTATTTTATTTTATTTTTATTTTATTTTATTTTATTTATTTTATTTTATTTTATTTTATTTTATTTTTAT
TTTATTTTATTTTATTTTATTTTTATTTTATTTTTATTTTATTTTATTTTATTTTATTTTATTTTATTTTTATTT
TATTTTATTTTATTTTATTTTATTTTATTTTTATTTTATTTTATTTTATTTTATTTTATTTTATTTTATTTATT
TTATTTTATTTTATTTTATTTTATTTTATTTTATTTTATTTTATTTTATTTTATTTTTATTTTATTTTATTTTAT
TTTATTTTATTTTATTTTATTTTATTTTATTTTATTTTATTTTATTTTATTTTATTTTATTTTATTTTATTTTA
TTTTATTTTTATTTTTATTTTTATTTTATTTTATTTTGTTTTATTTTATTTTATTTTATTTTATTTTATTTTATT
TTATTTTATTTTATTTTTATTTTTATTTTATTTTATTTTATTTTATTTTATTTTATTTTATTTTATTTTATTTT
ATTTTATTTTTATTTTATTTTATTTTATTTTATTTTATTTTATTTTATTTTTATTTTATTTTATTTTATTTTATT
TTATTTTTATTTTATTTTATTTTTATTTTATTTTATTTTATTTTATTTTATTTTTATTTTATTTTATTTTATTT
TATTTTATTTTATTTTATTTTATTTTTATTTTATTTTATTTTTATTTTTATTTTTATTTTATTTTATTTTATTT
TATTTTATTTTATTTTATTTTATTTTATTTTATTTTTATTTTATTTTATTTATTTGTTTTATTTTATTTTATTT
TATTTTATTTTATTTTATTTTTATTTTATTTTATTTTTATTTTATTTTATTTTATTTTATTTTATTTTTATTTTA
TTTATTTTATTTTATTTTATTTTATTTTATTTTATTTTATTTTTTTATTTTATTTTATTTATTTTATTT
TATTTTATTTTATTTTATTTTTATTTTATTTTATTTTATTTTATTTTATTTATTTTATTTTATTTTATTTTATT
TTATTTTTATTTTATTTTATTTTATTTTATTTTGTTTTTATTTTATTTTATTTATTTTATTTTATTTTATTTTA
TTTTATTTTATTTTATTTTATTTTATTTTATTTTATTTTATTTTATTTTATTTTTTATTTTATTTTATTT
TTATTTTATTTTATTTTATTTTTATTTTATTTTATTTTATTTTTATTTTATTTTATTTTATTTTATTTTAGGTT
TTATTTTATTTTATTTTATTTTATTTTATTTTATTTTATTTTATTTTATTTTATTTTATTTTATTTTATTTTTA
TTTTATTTTTTATTTTATTTTATTTTTATTTTATTTTATTTTTATTTTTATTTTATTTTATTTTATTTTATTTT

ATTTTATTTTTATTTTATTTTATTTTATTTTATTTTATTTTATTTTATTTATTTTATTTTATTTTATTTTATTT
TTATTTTATTTTATTTTATTTTATTTTATTTTATTTTTATTTTATTTTTATTTTGTTTTATTTTATTTTATTTT
ATTTTATTTTATTTTATTTTATTTTATTTTATTTTATTTTATTTTATTTTATTTTTATTTTATTTTATTTTATT
TTATTATTTTATTTTATTTTATTTTATTTTATTTTTATTTTTATTTTATTTTATTTTATTTATTTTATTTTTATTTTAT
TTTATTTTATTTTTATTTTATTTTTATTTTTATTTTATTTTATTTTATTTTATTTTATTTTATTTTATTTTATT
TTATTTTTATTTTTTATTTTATTTTATTTTATTTTATTTTATTTTTATTTTATTTTATTTTATTTTATTTTATT
TTATTTTATTTTATTTTTATTTATTTTATTTTTATTTTATTTTATTTTATTTTATTTTATTTTATTTTATTTAT
TTTATTTTATTTTTATTTATTTTATTTTATTTTATTTTATTTTATTTTATTTTATTTATTTTTATTTTTATTTT
ATTTTTATTTTATTTTATTTTATTTTTATTTTATTTTATTTTATTTTATTTTATTTTATTTTTATTTTTATTTTA
TTTTATTTTATTTTTATTTTTATTTTATTTTATTTTTATTTTATTTATTTTATTTTATTTTATTTTATTTTATT
TTGTTTTATTTTATTTTATTTTATTTTATTTTTATTTTATTTTTATTTTATTTTATTTTATTTTTATTTTATTT
TATTTTATTTTATTTTATTTTATTTGGTTTGTTTTTATTTTATTTTTATTTTATTTTATTTTATTTATTTTATT
TTATTTTATTTTATTTTATTTTATTTTATTTTATTTTATTTTATTTTATTTTATTTTATTTTATTTTATTTTAT
TTTATTTTATTTTATTTTATTTTATTTTTATTTTATTTTTATTTTATTTTATTTTTATTTTATTTTATTTTATT
TTTATTTTATTTTTATTTTATTTTATTTTATTTTATTTTTATTTTATTTTATTTTATTTTATTTTATTTTATTT
TATTTTATTTTATTTTATTTTATTTTATTTTTATTTTATTTTATTTTATTTTGGTTTATTTCTATTTCATTTTT
ATTTCATTTCATTTCATTTTCATTTATTTCATTTTGTTTCTCATTTCATTTCATTTCTTATTTATTTCTTATTT
CTATTTCATTTATTTTCATTTCATTTCTTATTTCTATTTATTTCTTTATTTCATTTCTTATTTATTTATTTCTA
TTTATTTCTTATTTCTTATTTCTATTTATTTTCTATTTCTTATTTATTTTATTTCATTTATTTCTATTTCTATT
TCATTTCTTATTTCTTATTTCTATTTATTTATTTCATTTATTTATTTATTTCATTTCTATTTCATTTCATTTCA
TTTCTATTTCTATTTCTATTTCTATTTCTATTTCTTATTTCTATTTATTTATTTCATTTTATTTCATTTATTTC
TATTTATTTATTTCATTTATTTATATTTCTATTTCTATTTCTTATTTCTTATTTATTTATTTATTTCATTTTCT
TATTTCCTTATTTCTATTTCTATTTCTATTTTATTTTCATTTATTTCTATTTATTTATTTCATTTATTTATTTC
TTATTTATTTCTATTTTCTATTTATTTATTTCTTATTTATTTCTATTTATTTATTTCTATTTCTATTTCTTATT
TATTTCTATTTCTATTTCTTATTTATTTATTTCTATTTCTATTTATTTTATTTCTATTTCTATTTATTTATTTA
TTTTTATTTTATTTCTTATTTCTTATTTCTTATTTCTATTTATTTATTTATTTCTATTTTCTTATTTATTTATT
TCTATTTTCTTATTTCTATTTTATTTTATTTCTATTTCTATTTCTATTTATTTATTTATTTCTTTTATTTATTT
ATTTCATTTCTTATTTCTTATTTCATTTTATTTCTTATTTTCATTTCTATTTTCATTTCTTATTTCTATTTTAT
TTCTTATTTATTTATTTCTTTATTTCTATTTCTATTTCTATTTATTTTCTATTTATTTTCATTTCTATTTCATT
TCTATTTCTATTTATTTCTATTTTATTTCATTTCTATTTATTTCTATTTCTATTTATTTCTTATTTCTATTTCT
ATTTCTTATTTCATTTCTATTTCTATTTTATTTCTATTTATTTCTATTTCATTTATTTATTTATTTCTATTTCT
TATTTCTATTTCTTATTTCTATTTATTTATTTCTATTTCATTTCATTTCATTTCTATTTTCTATTTTCATTTCT
ATTTCTTATTTATTTATTTCTATTTCTCTATTTTCATTTATTTCATTTCTTATTTCTATTTCTATTTTCTTTAT
TTCATTTTTCATTTCTATTTCATTTCATTTCATTTCATTTACTTCATTTCTATTTCATTTCTATTTCATTTCAG
TTTTAGATGGA

d) 1-IV-6_4 (+)
AAAAAAAATCGGTATCACCTACTTTTTAGCTTTCCACTTATACCAATTTATTGGTTCTGTTTTTTGTTGTTATT
TTTTATTTTATTTTATTTTATTTTTATTTTTATTTTGTTTTTATTTTATTTTATTTTGTTTTCATTTATTTTT
GTTTTATTTTTATTTTGTTTTATTTTTGTTTATTTTTATTTTTTATTATTTTTATTTTTGTTTATTTTATTTTT
GTTTTTTGTTTTTCCCATTTTTATTTTTTATTTTATTTTATTTTGTTTTGTTTTGTTTTGTTTTTCATTTTTTA
TTTTTATTTTTGTTTTATTTTTATTTTATTTTATTTTTTATTTTGTTTTTTATTTTTTATTTTATTTTTTGTTT
TTATTTTATTTTATTTTGTTTGTTTTTGTTTTATTTTATTTTTTTTATTTTTTGTTTTTGTTTGTTTATTTTGT
TTTTATTTTGATTTTTGATATTTTTATTTTATTTTATTTTTGTTATTTTTTGTTTTTTATTTTGTTTGTTTATT
TTGTTTTTGTTTTGTTTTGTTTGTTTTTGTTTTGTTTTTTTATTTTTGTTTTTGTTTTTTGTTTATTTTATTTT
TTGTTTTTGTTTTTTTGTTTTATTTTGTTATTTTGTTTTTCGCATTTTTGTTGTTTTTTGTTTGTTTTGTTTTT
TGTTTGTTTTGATTTTTGTTTTTATTTTATTTTTGTTTTGACACATTTTATTTTATTTTATTTTTATTTTATTT
TTGACCTTGTTTTATTTTTTGTTTTATTTGTTTATTTTATTTTTATTTTTTGTTTTGTTTTATTTTGTTTTTGT
TTTATTTTTGTTTTATTTTTTGTTTATTTTTATTTTGTTTTTATTTTATTTTTATTTTTTATTTGTTTATTTTG
TTTTATTTTTATTTTATTTTTATTTTATTTTATTTTATTTTTATTTTATTTTTGATTTTTATTTTGTTTATTTT
ATTTTGTTATTTTTATTTTGTTTTATTTTTATTTTGTTTTGTTTTTATTTTTTGTTTTTTATTTGTTGTTTTAT
TTTGATTTTTATTTTTTATTTTATTTTATTTTTATTTTGTTTTTATTTTATTTTTATTTTATTTTGTTTATTTTT
ATTTTTTATTTTTATTTTGTTTTTATTTTATTTTATTTTTTATTTTATTTTTATTTTTATTTTTTATTTTATTT
GTTTTGTTTATTTTTATTTTTTTATTTTTATTTTATTTTGATTTTATTTTATTTTTTGTTTTTATTTTTATTTT

TATTTTTTTATTTTTGTTTTATTTTATTTTATTTTTATTTTATTTTATTTTATTTTTTATTTTGACTTTATTTT
TATTTTGTTTTTTATTTTATTTTATTTTATTTTTGTTTTGTTTTGTTGTTTTATTTTATTTTTATTTTTTATTT
TTGTTTATTTTTTATTTTATTTTTATTTTTTTGTTTATTTATTTTATTTTTATTTTATTTTTGTTTTTGTTTAT
TTTATTTTATTTTATTTTTATTTTGTTTATTTTGTTTTGTTTTGTTTTTTATTTTTTATTTTATTTTATTTTAT
TTTTATTTTATTTTGTTTTTATTTTTATTTTGTTTATTTTTATTTTGATGTTTGTTTTATTTTTTATTTTTTGC
ATTTTATTTTTATTTTGCATTTTTATTTTTATTTATTTTTATTTTGTTTTGTTTTTGTTTTATTTTGTTTATTT
TATTTTATTTTATTTTATTTTTGTTTTTATTTTTTGTTTTATTTTGTTTGTTTTATTTTATTTATTTTGTTTAT
TTTTATTTTATTTTATTTTGTTTGTTTGTTTTTATTTTTATTTTTATTTTTTATTTTATTTTTGTTTTTATTTT
ATTTTATTTTTTATTTTATTTTATTTTATTTTTTATTTTATTTTATTTTTATTTTTATTTTTGTTTTTGTTTTT
ATTTTTTGTTTTTATTTTGTTTGATTTTTATTTTTATTTTTTGTTTTATTTTTATTTTGTTTTTGTTTTGTTTG
TTTTTGTTTTTGTTTTTGTTTTTATTTTTGTTTATTTGTTTTGTTTTATTTTTGTTTTGTTTTATTTTTTGTTT
TTATTTTTTTATTTTGTTTATTTTTGTTTATTTTGTTGTTTTGTTTTGTTTTTTGTTTTTTGTTTTTTGTTTTTG
TTTTGTTTGTTTTATTTTGTTTATTTTTTATTTTTGTTTTGTTTTTGTTTTGTTTTGTTTTTTATTTTGTTTTG
TTTTTATTTTTGTTTTATTTTTGATTTTTGATGCTTGTTTGTTTTTGTTTATTTTGTTTTATTTTGTTGTTTGT
TTTGTTTTGTTTTGTTTTTATTTTATTTTTTATTTTTATTTTGTTTTTGTTTTTATTTTTTGTTTTGTTTTGTT
TATTTTTGTTTTATTTTGTTTGTTTTATTTTTTGTTTTTGTTTTGTTTATTTTGTTTTATTTTGTTTTATTTTG
ATTTTTATTTTTTGTTTATTTTTTATTTATTTTGTTTATTTTTGTTTTATTTTGTTTTGTTTTGTTTTGTTTTT
ATTTTTGATTTATTTTATTTTTATTTTATTTTATTTTATTTTATTTTATTTTATTTTTATTTTATTTTATTTTA
TTTTTATTTTTTATTTTATTTTGTTATTTTATTTTATTTTATTTTTATTTATTTTTTTATTTTATTTTATTTTA
TTTTGTTTTTGACTTTTATTTTATTTTATTTTTATTTTTGTTTTGTTGTTTTTATTTTGTTTTATTTTATTTAT
TTTATTTTTATTTATTTTATTTTTATTTTTGTTTTGTTTGTTTTGTTTTGTTTTTTATTTTTATTTATTTTGTT
TTTTATTTTGTTTGTTTTTTTATTTTTGTTTTGTTATTTTATTTTGTTTTGTTTTAATTTTTGTTTACAATTTT
ATTTTATTTTGTTTTGTTTTTGAAATTTTTATTTTTATTTTGTTTATTTTATTTTGTTTATTTTGTTTTGTTTT
GTTTTGTTTTTGTTTTGGCTTTTTGTTTGTTTTGTTTTGTTTTTGTTGCATTTTGTTTTGTTTTATTTTTGTTT
TGTTTTATTTTGTTTTGTTTTTTATTTTTGTTTTTGTTTTTATTTTTTTGTTTTTGTTTTGTTTTTGTTTGTTT
TTATTTTTCCCATTTTTATTTTGTTTTGTTTTGTTTTTTGTTTTTTGTTTTGTTTTATTTTTATTTTGTTTTTG
TTTTTTGTTTATTTTGTTTTTGATTTTATTTTACATTTTTGTTTATTTTTGTTTTGTTTTTTGTTTTTTGTTTT
GTTTTGTTTTACATTTTATTTTTGTTTTGTTTTGTTTTATTTTCATTTTTGTTTTGTTTTGTTTTGTTTTGTTT
TATTTTGTTTTTGATTTTATTTTTTGTTTTATTTTTGTTTTGTTTCACATTTTCATTTCCTTTTTCATTTCATT
TCATTTCATTTCATTTTCATTTCATTTCATTTCATTTTCATTTCATTTCATTTCTTTCACATTTCATTCATTTC
GCTTTCATTTCATTTCATTTCATTTTCCATTTCATTTCATTTCATTTTCATTTCATTTCGACATTTTCATTTCA
TTTCATTTCATTTCATTTCATTTTCATTTCATTTCATTTCATTTGTTTTCATTTCGATTTCATTTCATTTCATT
TCATTTCATTTCATTTCATTTCATTTCATTTCGTTGTTTTCATTTTTCGTTTCAATTTCATTTCGTTTCATTTC
ATTTCGTTTCATTTCATTTCATTTCATTTTTCGATTTCGTTTCATTTTCATTTCGTTTCATTTCATTTTCATTT
CATTTCATTTCGTTTCATTTCATTCCGAACTTTCATTTTCATTTTCCACGTTTCATTTCATTTTTCATTTCATT
TCATTTCATTTTCATTCATTTCATTTCATTTCATTTCATTTCATTTCATTTCATTCATTTCATTTTCATTTCAT
TTCATTTCATTTTCATTTCATTTCATTTCATTTCATTTCATTTCACGTTTTCATTTCATTTCATTTCATTTCAT
TTCATTTCATTTCATTTCATTTCATTTCATTTTCAATTTCATTTCATTTCATTTCATTTCATTTCATTTCATTT
CATTTCATTTCATTTCATTTCATTTCATTTTCTTTCATTTTTTCATTTCATTTCCATTTTCATTTCGAACAATT
TCGTTTCATTTTCATTTCATTTCATTTCGTTTCATTCAATTCTAGAACTTTCATTTCATTTCGTTTCATTTCAT
TTCATTTCGTTTCGATTTCGTTTCATTTTCTGTTCATTTCATTTCATTTCATTTCGATTTCATTTCATTTCATT
TCATTTCATTTCATTTCATTTCATTTTTCATTCATTTCGTTTCATTTCATTTCATTTCATTTCGTTTCATTTCA
TTTCATTTCATTTCATTTCATTTCATTTCATTTCATTTCGTTTTGTTTCATTTCATTTCATTTTCATTTCATTT
GAACTTCATTTCATTTCATTTCATTTTCATTTCATTTCATTTCATTTCGTTTCATTTCGTTTTTCATTTCATT
TCGTTTCATTTTCATTTCATTTTCATTTCATTTCATTTTCATTTTCATTTCATTCATTTCGTTTCATTTCGTTT
TCATTTCATTTTCATTTCGTTTTCATTTCATTTCATTTCGTTTCATTTTGTTTTCATTTCATTTCATTTTCATT
TCAACAAATTTCATTTTCATTTCGATTTCATTTCATTTTCATTTCATTTTTGTTTCATTTCATTTCATTTTCAT
TTCATTTCATTTCATTTCATTTCATTTCATTTCATTCATTTCATTTCATTTCATTTCATTTCATTTCATTTCAT
TTTCATTTCATTTCATTTCATTTCATTTCATTTCATTTCATTTCATTTCATTTCATTTTCATTTCATTTCATTT
CATTTCATTTCATTTTCATTTCATTTCATTTCATTTTCATTTCATTTCATTTCATTTCATTTCATTTCATTTCA
TTTCATTTCATTTTCGTTTCCATTTCATTTCATTGTTCATTTTTAATTTAGTTTCATTTCATTTCATTTCATTT
TCATTTCATTTGTTTCATTTCATTTCATTTCCATTTTCATTTCGTTTCATTTCATTTCATTTCATTTTCATTTC
ATTTCATTTCATTTTCTAGTTCATTTCATTTTCATTTCATTTCATTTCATTTCATTTCATTTCATTTTCATTTC
ATTTCATTTCATTCATTTCATTTCATTCTTTAGTTTCGAACTTCCATTTCATTTCATTTTCGAGGATGGA

e) 1-IV-8_1 (-)

GAAAACACTATACTGTTGTGGTTCTGTTTTTTTTTATTTTATTTTATTTTTTATTTTATTTTATTTTATTTTAT
TTTATTTTTTAGTTTATTTATTTTATTTTATTTTTATTTTATTTTTATTTTATTTATTTTATTTTATTTTATT
TTATTTTATTTTATTTTATTTTATTTTATTTTATTTTATTTTATTTTATTTTTATTTTATTTTATTTTATTTTA
TTTTATTCTTATTTTATTTTATTTTAGTTTTATTTTATTTTATTTTATTTTATTTTATTTTATTTTATTTTATT
TTTATTTTATTTTTATTTCTTATTTTATTTTATTTTATTTTATTTTATTTTTATTTTTATTTTTATTTTATTT
TATTTTTATTTTATTTTATTTTATTTTTATTTTATTTTATTTTATTTTATTTTATTTTATTTTATTTTATTTTA
TTTTATTTTATTTTATTTTATTTTATTCTTTTATTTCCATTTTATTTTATTTTATTTTATTTTATTTTATTTTA
TTTTATTTTCTGGTTTTAATTTTTATTTTTATTTTTATTTTATTTTATTTTTATTTTATTTTTATTTTATTTTA
TTTTATTTTATTTTATTTATTTTATTTTATTTTATTTTATTTTATTTTATTTTATTTTATTTTATTTTATTTTA
TTTTATTTATTTTATTTTATTTTATTTTATTTATTTTTATTTTATTTTATTTTATTTATTTTATTTTATTTTAT
TTTTATTTTATTTTTATTTATTTTATTTTTAGTTTTATTTTTATTTTTTATTTTATTTTATTTTCTTATTTATT
TCTTATTTTATTTTATTTTTATTTATTTTATTTTATTTTTATTTTATTTTATTTTTATTTATTTTATTTTATTT
TATTTTATTTTATTTTATTTTATTTTATTTTATTTTTATTTTTATTTTATTTTATTTTTATTTTATTTATTTCA
TTTTTATTTTATTTTATTTTTATTTTATTTTATTTTATTTATTTTATTTTATTTATTTATTTTATTTTATTTTT
ATTTTATTTTTATTTTATTATTTTTAGGTTTTTATTTTATTTTTATTTTTATTTTATTTTTATTTTATTTTATT
TTATTTTATTTTATTTTATTTTATTTTATTTATTTTTATTTTATTTTATTTTATTTTATTTATTTTATTTTATT
TTTATTTTATTTTATTTTTATTTTATTTTTATTTTATTTTATTTTTATTTTATTTTATTTTATTTATTTTATTT
AGTTTTATTTTTATTTTATTTGTCAAGAGTTCTCTATTTTATTTTTATTTTATTTTATTTTATTTTATTCTATT
TTATTTTATTTAACTTATTTTATTTTCATTTTATTTTATTTTATTTTTATTTTATTTTTATTTTATTTTATTTT
ATTTTATTTTATTTTTATTTTATTTCTATTTATTTTATTTTTATTTTTATTTTATTTTATTTTATTTTATTTTT
ATTTTTATTTTATTTTTATTTTTATTTTATTTTATTTTATTTTTATTTTTATTTATTTATTTTATTTTATTTAT
TTTATTTTATTTTTATTTTATTTTATTTATTTTTATTTTATTTTATTTTTATTTTATTTTTATTTTATTTTATT
TTATTTTATTTTATTTTATTTTTATTTAGAGATTAGATATTTATTTATTATTTATATTTATTATTTTTATTTTA
TCTTTATTTTATTTATTTTATTTTATTTTATTTTATCTCTTATTTTTATTTTATTTTTATTTTATTTTATTTTA
TTTTATTTAATATTTATTTTATTTTATTTTTATTTTATTTTATTTTATTTTTATTTTATTTTTTATTTTATTTT
ATTTTATTTTTATTTTTATTTTATTTTTTATTTTTATTTTATTTTTATTTTATTTTATTTATTTTATTTTATTT
TATTTTATTTCATTCTGTGTCTGAAATCTTTATTTTATTTTATTTTATTTTATTTATTTTATTTTATTTTATTT
ATTTATTTATTTATTTTATTTATTTTATTTATTTTATTTTATTTTATTTTATTTTATTTTATTTTTTATTTTAT
TTTTAGTTTTATTTATTTTGGTTTTATTTTATTTTATTTTATTTTATTTTATTTTTTATTTTATTTTATTATTT
TATTTTATTTTATTTTTATTTTATTTTATTTTATTTTATTTATTTTATTTATTTTTATTTTTATTTTATGATGA
TTATTTTATTTATTCTGTTTTATTTTATTTTATTTTATTTTATTTTATTTTATTTTATTTATTTTATTTTATTT
ATTTTTATTTTATTTTATTTTTATTTTATTTTATTTTATTTATTTTATTTTATTTTATTTTATTTTATTTTATT
TTATTTTATTTTATTTTTATTTTATTTTATTTTTTATTTTTATTTTATTTATTTTATTTTTATTTTATTTTTTA
TTTTATTTTTATTTATTTTTATTTTATTTTATTTTATTTTTATTTATTTTATTTTATTTTTATTTATTTTTATT
TTATTTTATTTTATTTATTTTTATTTTATTTTATTTATTTTATTTTATTTTTATTTTATTTTATTTTATTTTAT
TTTATTTTATTTTATTTTATTTTATTTTATTTTATTTTATTTTATTTATTTTATTTTTATTTTTATTTTATTTATT
TATTTTAGTTTTATTTTATTTTTATTTTATTTATTTTATTTTATTTTTATTTTATTTTATTTTTTATTTTTATT
TTATTTTATTTTTATTTTTATTTATTTTATTTTATTTTATTTTATTTTATTTTATTTTATTTTTATTTTATTTT
ATTTTTATTTTATTTTATTTTATTTTTATTTATTTTATTTTATTTTATTTTTATTTTATTTTATTTTATTTTAT
TTTATTTTATTTTATTTATTTTTATTTTATTTTATTTTATTTTTTTATTTTTATTTATTTTATTTTTATTTCTA
TTTATTTCATTTCTTATTTCATTTATTCATTTCATTTCATTTATTTCCTTATTTTATTTATTTCTATTTCATTA
TTTATTTATTTCATTTCATTTCTATTTCTATTTCTATTTCTTATTATTTTCATTTATTTCATTTTCATTTATTT
CTTATTTTATTTATTTCTTATTTATTTCATTTATTTATTTATTTATTTCTTATTTCTATTTCTATTTTATTTCT
ATTTCTATTTTTATTTATTTCTTATTTATTTTATTTTCTTATTTTCTATTTTCATTTCTTATTTATTTATTTCT
ATTTCTATTTCTATTTCTATTTCTATTTCTATTTATTTTTATTTTATTTATTTATTTTATTTATTTTCTATTTC
TATTTCTTATTTCTATTTATTTATTTCATTTTATTTCTATTTATTTATTTCTATTTTCTAGTTTATTTATTTAT
TTCTATTTTCTTATTTCTATTTCTTATTTATTTATTTCTATTTATTTCTTATTTCTTATTTATTTATTTTATTT
CTATTATTTATTTCTATTTCTATTTATTTCTTATTTATTTTCTTATTTCTATTTATTTCTATTTATTTCTATTT
CTATTATTTATTTATTTCTATTTTATTTCTATTTATTATTTTATTTATTTATTTCATTTATTTATTTCTATTTA
TTTCATTTCTATTTCTTATTTTCTATTTTCTATTTTCTATTTCTATTTCTTATTTCTTATTTCTTATTTCTATT
TCTATTTCTATTTCTTATTTCTATTTCTTATTTTATTTCTATTTCTATTTATTTCTTATTTTATTTATTTCTAT
TTATTTATTTATTTCTTATTTATTTCTTATTTCTATTTATTTCTATTTCTTATTTCTATTTATTTCTATTTATT

TCTTATTTATTTTCTATTTCTATTTATTTTATTTTCTATTTCTATTTCTATTTCTTATTTCTTATTTTCATTTC
TTATTTATTTATTTCTATTTCTATTTCTTATTTATTTCTATTTCATTTATTTTTATTTCTTTATTTCATTTCTA
TTTCTATTTATTTCTATTTCTATTTCTATTTATTTCTTATTTCTTATTTATTTATTTATTTTATTTTATTTATTT
ATTTTTCTATTTATTTCTTATTTATTTATTTCTATTTCTATTTCATTTCTTATTTCTATTTCTTATTTTCTATT
TATTTATTTCTATTTATTTCTTATTTCTATTTCTATTTCTATTTATTTATTTTGCTTATTTTCTTTTATTTATT
TATTTCTTATTTCTTATTTCTATTTCTTATTTCTATTTCTATTTCTTATTTCTATTTCTATTTCATTTATTTCT
TATTTCTATTTCTATTTCTACTTATTTCATTTCATTTCTATTTCTATTTATTTCTTATTTTTCATTTCATTTTC
ATTTCTATTTCATTTCATTTCTCTATCATTTATTTTCATTTCATTTCATTTCATTTCTATTTCATTTCATATTT
CATTTCTTTACTCTTCTATTTCCTTATTTCATTTCATTTCTATTTTTCATTTCTTACTTATTTCATTTCCTTAT
TTCATTTTTATTTCTTTATTTCATTTCTTATTTCATTTATTTCATTTCATTTCATTTTCATTTCATTTCATTTC
TATTTCATTTCATTTCTATTTCATTTCTCATTTCTATTTCATTTTCATTTCATTTCATTTCATTTCTTATTTCA
TTTTCTTTATTTCTATTTCTTATTTCTATTTCATTTCTATTTCTTCTATTTCCTTATTTCTATTTCATTTCATT
TATTTTCATTTATTTCATTTCATTTCTATTTCTATTTCTTATTTCTATTTCATTTCTATTTCATTTCTATTTCA
TTTCTATTTATTTCTATTTCTATTTCTATTTATTTCTATTTCTATTTCTATTTCATTATTTCTTTATTTATTTC
TATTTCTATTTCATTTATTTCTATTTCTATTTCTATTTCTATTTATTTCATTTCTTTATTTCTATTTCATTTCT
ATATTTTCTATTTTATTTCTATTTCTATTTTATTTCTTATTTATTTCTATTTATTTCATCATTTTCATTTCATT
TTATTTATTTTCATTTCTATTTATTTTCTATTTCATTTCATTCATTTCTATTTCTATTTCTATTTATTTATTTC
ATTTATTTCATTTACTTATTTCTATTTCATTTCTATTTCTATTTATTTCCATTTTTCTATTTTCTGAGATGGA

f) 1-IV-9_1 (+)

GAAAACACTTACATACTGTTGTGGTTTCTGTTTTTTATTTATTTTATTTTATTTTATTTTTATTTTTATTTTTT
ATTTTATTTTATTTTATTTTATTTATTTTATTTTATTTTTGTTTATTTTATTTTTATTTTATTTTATTTTTATT
TTTATTTTATTTTACATTTGTTTTTATTTTATTTTTTATTTTATTTTATTTTTATTTTATTTATTTTTGTTTTA
TTTTATTTTATTTTTATTTTTATTTTATTTTATTTTTTATTTTATTTTATTTTATTTTATTTTATTTTTTATTTT
ATTTTTATTTTTTATTTTATTTTATTTTATTTTTTATTTTATTTTATTTTATTTTATTTTATTTTATTTTATTT
TATTTTATTTTATTTTTATTTTTATTTTATTTTATTTTATTTTATTTTATTTTATTTATTTTATTTTTTATTTT
ATTTTATTTTTTATTTTATTTTATTTTTATTTTATTTTATTTTTATTTTATTTTTTATTTTATTTTTATTTTTA
TTTTATTTTATTTTTATTTTATTTATTTTATTTTTATTTTATTTTATTTTTATTTTTATTTTATTTTTATTTTA
TTTTATTTTTATTTTATTTTTATTTTATTTTATTTTATTTTTATTTTATTTTTATTTTATTTTTATTTTTATT
TTATTTTTATTTTATTTTATTTTATTTTATTTTATTTTATTTTTATTTTATTTTTATTTTATTTTTATTTTATT
TTATTTTATTTTATTTTATTTTATTTTTATTTTTTATTTTATTTTATTTTTATTTTATTTTTATTTTTATTTT
ATTTTATTTTATTTATTTTATTTTATTTTTATTTTATTTTATTTTATTTTTATTTTATTTTTGTTTTA
TTTTTATTTTTATTTTATTTTATTTTTATTTTATTTTATTTTATTTTATTTTATTTTATTTTTATTTTAT
TTTATTTTATTTTATTTTATTTTATTTTATTTTATTTTATTTTATTTTATTTTTATTTTATTTTATTTT
ATTTTATTTTTATTTTATTTTTATTTATTTTATTTTATTTTTATTTTTATTTTATTTTTATTTTTATTT
TTATTTTTATTTTATTTTATTTTATTTTATTTTTATTTTTATTTTATTTTTATTTTTATTTTTTATTTTTATTT
TTATTTTATTTTTATTTTATTTTATTTTATTTTATTTTATTTTTATTTTATTTTATTTTATTTTTATTTTATTTT
ATTTTTATTTTTCATTTTATTTTATTTTATTTTATTTTTATTTTTATTTTTATTTTATTTTTATTTTTATTTTAT
TTTATTTTATTTTTTATTTTTTATTTTATTTTTATTTTATTTTATTTTTTATTTTTATTTTATTTTTATTTTTA
TTTTTATTTTATTTTATTTTATTTTTATTTTATTTTATTTTATTTTATTTATTTTTATTTATTTTTATTTTTAT
TTTATTTTATTTTATTTTATTTTATTTTATTTTATTTTATTTTATTTATTTTATTTTATTTTTATTTTA
TTTTATTTTATTTTATTTTATTTATTTTATTTATTTTATTTTGTTTTGTTTGTTTGTTTTTTGTTTTTATTTTT
ATTTTTATTTTATTTTTTATTTTATTTTATTTTTATTTTTATTTTTATTTTTTATTTTATTTTTTATTTTTTTATTT
TTGTTTTATTTTATTTTTATTTTTTATTTTTATTTTTATTTTATTTTATTTTTGTTTTTTATTTTATTT
TTTATTTTATTTTTATTTTTTATTTTTATTTTTTATTTTTATTTTTATTTTTGTTTATTTTTATTTTT
TTATTTTTATTTTTATTTTTTATTTTTATTTTATTTTTATTTTTATTTTATTTTTATTTTTATTTTTTTATTTTTAT
TTTATTTTATTTTTATTTTTGTTTTATTTTTATTTTTATTTTATTTTTTATTTTTTATTTTTCATTTTTTTATT
TTATTTTTATTTTGTTTTATTTTGTTTTGTTTTATTTGTTTTATTTTATTTTTTTGTTTTATTTTATTTTTATT
TTATTTTATTTTTTATTTTTATTTTATTTTTATTTTATTTTTATTTTATTTATTTTATTTTATTTTATTTGTTT
TTTATTTTTTTATTTTATTTTATTTATTTTATTTTTTATTTTTATTTGTTTTGTTTTGTTTTTATTTTGTTTTTA
TTTTTTGTTTTTATTTTTTATTTTTATTTTTATTTTGTTTATTTTTTATTTTTTATTTTTTATTTTTATTTTTG
TTTTTTATTTTTGTTTTTGTTTTATTTTTGTTTTATTTTTGTTTTTATTTTGTTTTATTTTTTTGTTTTTATTTT
TTATTTTGTTTTATTTTTGATTTTTGTTTGTTTTGTTTTTGTTTTATTTTTATTTTGTTTTATTTTATTTTTTT
GTTTTTGTTTTATTTTTATTTTGTTTTGTTTTTTATTTTATTTTATTTTTATTTTTTTTGTTTGTTTATTTTGT

TTATTTTTTGTTTTGTTTTTTGATTTTTGTTTTTATTTTATTTTTGTTTTTTATTTTTGTTTTATTTTTTGTTTT
TGTTTTTATTTTTGTTTATTTTTGTTTTGTTTTGTTTGTTTTGTTTTGTTTTATTTTTATTTTTATTTTTATTT
TATTTTTGTTTTGTTTTGTTTTATTTTGTTTTTGTTTTTTATTTTTTATTTTATTTTGATTTTGTTTTTATTTT
TTATTTTTTATTTTTTATTTTTATTTTTGTTTTTATTTTTTATTTTATTTTTATTTTTATTTTTATTTTTATTT
TATTTTATTTTGTTTATTTTTGTTTATTTTTGTTGTTTTTGTTTTGTTTTTATTTTTTATTTTTTGTTTTTGTT
TTTGTTTTTGTTTTTGTTTTTATTTTTGTTTTTGTTTTTGTTTTGTTTATTTTATTTTTATTTTTGTTTTTGTT
TTTATTTTGTTTTTTATTTTTTATTTTTTATTTTTGTTTTTGTTTTGTTTATTTTTTATTTTTTTTGTTTTTGT
TTTTGTTTTTGTTTTTTGTTTTTTGTTTTTTGTTTTTTGTTTTTGTTTTTGTTTTATTTTTATTTTTGTTTTT
ATTTTATTTTATTTCATTTCATTTCATTTCATTTCATTTCATTTCATTTCATTTCATTTCATTTCATTTCGTTT
CATTTCGTTTCATTTCATTTCATTTCATTTCATTTCATTTCATTTCATTTCATTTCATTTCATTTCATTTCATT
TCATTTCATTTCATTTCATTTCATTTCATTTCATTTCATTTCATTTCATTCATTTCATTTCATTTCATTTCATT
TCATTTTCATTTCATTTCATTTCATTTCATTTCATTCATTTCATTTCATTTCATTTCATTTCATTTCATTTCAT
TTCATTTCATTTCATTTCATTTCATTTCATTTCATTTCATTTCATTCATTTCATTTCATTTCATTTCATTTCTG
TTTCAATTTCATTTCATTTCATTTCATTTCTTCATTTCATTTCATTTCATTTCATTTCATTTCATTTCATTTCA
TTTCATTTCATTTCATTTCATTTCATTTCATTTCATTTCATTTCATTTCATTTCATTTCATTTCATTTCATTTC
ATTTCATTTCATTTCATTTCATTTCATTTCATTTCATTTCATTTCATTTCATTTCATTTCATTTCATTTCATTT
CATTTCATTTCATTTCATTTCATTTCATTTTCATTTCATTCATTTCATTTCATTTCATTTCATTTCATTTCATT
TCATTTCATTTCATTTCATTTTCATTTCATTTCATTTCATTTCATTTCATTTCATTTTCATTTCATTTCATTTC
ATTTCATTTTCATTTCATTTCATTTTCATTTCATTTCATTTCATTTCATTTCATTTCATTTCATTTCATTTTCA
TTTCATTTCATTTCATTTCATTTCATTTCATTTCATTTCGATGTTTTCATTTCATTTCATTTCATTTCA
TTTCATTTCATTTCATTTCTGACTTGTTTTTCATTTCATTTCATTTCATTTGTTTCATTTCATTTCATTTCATT
TCATTTCATTTCATTTCATTTCATTTCATTTCATTTCATTTCATTTCTGTTTTCATTTCATTTCATTTC
ATTTCATTTCTGTTTTGTTTCATTTCATTTTCATTTCATTTCATTTCATTTTCATTTTCGAGGATGA

g) 1-IV-11_1 (-)
GAAAACACTTATGTACTGTTGTGGTTCTTGTTTTTATTTATTTTATTTTATTTTATTTTATTTTATTTTTATTT
TATTTTATTTTTATTTATTTTTATTTATTTTATTTTATTTTATTTTATTTTATTTATTTTATTTTATTTTATTT
TATTTTATTTTATTTTTATTTTATTTTATTTTTATTTTTATTTTATTTTATTTTATTTTTTATTTTATTTTAT
TTTATTTTATTTTATTTTATTTTATTTTATTTTATTTTATTTTATTTTATTTTATTTTATTTTTATTTTTATTTT
TTATTTTATTTTATTTTATTTTATTTTATTTTATTTTATTTTTATTTTATTTTATTTTATTTTTATTTTATTTA
TTTTATTTTATTTTATTTTATTTTTATTTTATTTTATTTTTATTTTATTTTATTTTTATTTTATTTTATTTTTA
TTTTATTTTTATTTTATTTATTTTATTTTATTTTATTTTGTTTTATTTTATTTTATTTTATTTTATTTTATTTT
ATTTTATTTTTATTTTATTTTATTTTATTTTATTTTATTTTATTTTATTTTATTTTTATTTTATTTTATTTTAT
TTATTTTATTTTATTTTATTTTATTTTTATTTTGTTTTTATTTATTTTATTTTATTTTATTTTATTTGGTTTTA
TTTTTATTTTATTTTATTTATTTTATTTTATTTTATTTTATTTATTTTATTTTATTTTTATTTTATTTTATTTA
TTTTATTTTATTTTTGTTTATTTTATTTTATTTTATTTTATTTTATTTTATTTTATTTTATTTTATTTTATTTT
ATTTTATTTATTTTATTTTATTTTATTTTATTTTATTTTATTTTATTTTATTTTATTTTATTTTATTTTTATTT
TATTTTATTTTATTTTATTTATTTTATTTTATTTTATTTTATTTTATTTTTATTTTATTTTATTTTATTTTTAT
TTTATTTATTTTATTTTTATTTTTATTTTATTTATTTTGTTTTATTTTATTTTATTTATTTTTATTTTATTTTA
TTTTATTTTATTTTATTTTATTTTATTTTATTTATTTTTATTTTATTTATTTTATTTTATTTTATTTTATTTTA
TTTTATTTTATTTTATTTTATTTTATTTTATTTTATTTTTATTTTATTTTATTTTATTTTATTTTATTTTTATT
TTTATTTTATTTTATTTTATTTTATTTTATTTTATTTTATTTTATTTTATTTTATTTTATTTTATTTTATTTTA
TTTTATTTTATTTATTTTATTTTATTTATTTTATTTTTATTTTATTTATTTTATTTTATTTTATTTTTATTT
TATTTTATTTTTATTTTATTTTATTTTATTTTATTTTATTTTATTTTATTTTATTTTATTTCATCTTTATTTTC
ATTCCTATCTTATCTTCATCTTTATCTTATCTTATCTTCATCTTATTTCTTATTTTATTTTATTATTTTATTTT
ATTTTATTTGTTTATTTAGTTTTTATTTATTTTGTCTATTTATTTTTATTTTATTTTATTTTATTTATTTTATT
TTATTTTATTTTATTTTGTTTTATTGTTCATCTTCATTCATCTCTCATTCCTTATATTTTATCTTTTATCTTAT
TCTATCTTTATCTTATCTTTATCTTTATTTTATCTTTTATTTTATTTATTTTATTTTATTTTTATTTTATTTTA
TTTTATTTTATTTTATTTTATTTTATTTTATTTTATTTTATTTTATTTTATTTTATTTTATTTTATTTTATTTT
ACTTTTATTTTATTTTATTTTATTTTATTTTATTTTATTTTATTTTATTTTATTTTATTTTATTTTATTTTATT
TTATTTCTATTTTATTTTATTTTATTTTATTTTATTTTATTTTATTTTTATTTTCTATTTTATTTTTATTTTAT
TTTATTTATTTTTATTTTATTTTATTTATTTTATTTTATTTTATTTTATTTTATTTTTATTTTATTTTATTTTA
TTATGTTTTATTTTATTTTATTTTATTTTATTTTATTTTATTTTATTTTATTTTATTTTATTTATTTATTTTTA
TTTTTATTTTATTTTATTTTATTTTTAGTTTATTTTATTTTATTTTTATTTTATTTTATTTTATTTTATTTTAT

TTTATTTTTATTTTATTTTATTTTATTTTATTTTATTTTATTTTATTTTATTTTATTTTATTTTATTTTATTTT
TATTTTATTTATTTTTATTTTATTTTATTTTATTTTATTTTTATTTTATTTATTTTATTTTATTTTATTTTATT
TTATTTTATTTTTATTTTATTTTTATTTATTTTATTTTATTTTATTTTATTTTATTTTATTTTATTTTATTTTA
TTTTATTTTATTTTATTTTATTTTTATTTTATTTTATTTTATTTTATTTTATTTTATTTATTTTTATTTTATTT
TATTTTATTTTATTTTATTTATTTTATTTTATTTTATTTTATTTTATTTTATTTTATTTTATTTTATTTTATTT
TATTTTATTTTATTTTATTTTATTTTATTTTATTTTATTTTATTTTTATTTTATTTTATTTTATTTTATTTTAT
TTTATTTTATTTTATTTTATTTTATTTTAATGTTATTTTATTTGTTTTATTTTATTTTATTTTTATTTTATTTA
TTTTATTTTATTTTATTTTATTTTATTTTATTTTATTTTATTTTATTTTATTTTATTTTATTTTATTTTATTTA
TTTTATTTTATTTTATTTTATTTTATTTTATTTTATTTTTATTTTATTTATTTTATTTTTTTATTTATTT
TATTTATTTTTATTTTATTTTATTTTTATTTTATTTTATTTTTATTTTTATTTTATTTTATTTTATTTTATTTT
ATTTTGTTTTTATTTTATTTTATTTTATTTTATTTATTTTATTTTATTTTATTTTAAGTTTTGAGTTTTGGTTT
TGTTTTATTTTATTTCTATTTATTTATTTCTTATTTCTTATTTCATTTCATTTCTATTTCTATTTATTTCATTT
CTTATTTCTTATTTATTTCTATTTCATTTCATTTATTTCATTTCATTTTCATTTCATTTCATTTCATTTCATTT
CATTTCATTTCATTTCATTTCTATTTCTATTTATTTCTATTTATTTATTTATTTCTATTTTCTATTTCTATTTC
ATTTCATTTTCTATTTCATTTCATTTATTTCATTTTCATTTTTCATTTCATTTCATTTCATTTCATTTCATTTC
ATTTCTATTTATTTATTTCTTATATTTCTATTTCTTATTTTTATTTTCATTTATTTCATTTCATTTCATTTCAT
TTCATTTCTATTTCATTTTCATTTCTTATTTCTATTTATTTCATTTCATTTTCATTTCATTTCTATTTCTTATT
TCATTTCATTTCTATTTCATTTTCATTTCATTTCATTTATTTCTATTTCTATTTCATTTCATTTCTTATTTCTA
TTTCTTATTTTACTTATTTCTATTTTCATTTCATTTCATTTCATTTCATTTATTTCATTTCATTTTTATTTCAT
TTCATTTCTATTTCATTTCTATTTATTTCTATTTTCATTTCATTTCATTTCTTATTTCATTTCATTTATTTCAT
TTCATTTCATTTCTTCATTTCATTTCATTTCTATTTCATTTCATTTTATTTCTATTTCTATTTCTATTTCTATT
TCATTTCATTTCATTTCTTTATTTCTTATTTCATTTCTATTTCATTTCATTTCATTTCTATTTCATTTCATTTC
TTATTTCTATTTTATTTATTTCTATTTCTTTATTTATTTATTTATTTCTCTCTATTTATTTCTTATTTCATTTA
TTTTTATTTCTATTTATTTCATTTCTATTTCTATTTCATTTATTTCTATTTCGACATTTTATTTCTTTATTTAT
TTCATTTCATTTCTATTTATTTCATTTTCATTTCTATTTCATTTCTATTTCTATTTCTATTTTATTTCTATTTA
TCATTTCTATTTCATTTCTATTTCTATTTCATTTCTATTTTTATTTCTATTTTCTATTTATTTCATTTATTTCA
TTTATTTATTTCATTTCTTATTTCATTTCTATTTCATTTCATTTCATTTATTTTTATTTCTTATTTTTATTTCA
TTTCATTTCATTTCTATTTATTTTCTATTTCTTATTTCGTTTATTTTATTTTTATTTCTATTATTTCATTTATT
TTCATTTCATTTCATTTCTTATTTCTATTTCATTTCTATTTCATTTCTTTATTTCTTATTTATTTATTTTATTT
CATTTCATTTCATTTCATTTCATTTCTTATTTCTATTTTCATTTCTTTATTTTTATTTCTATTTCTATTTTCTA
TTTCTATTTCATTTCATTTTCATTTCATTTCTTTATATTTTCATTTCTATTTCATTTCATTTCTATTTCTATTT
CATTTCTACTTATTTCTATTTCATTTCTCATTTATTTCATTTCATTTCATTTCATTTCATATTTCTATTTATTT
CTATTTCTATTTCATTTCTATTTTCATTTTATTTCATTTATTTCATTTATTTCTATTTCGGTTTTTGGTTTATT
TCTATTTATTTCATTTCATTTCATTTCTATTTCATTTCATTTCTATTTCATTTCTATTTCATTTCATTTCATTT
CATTTCATTTCTATTTATTTCTTATTTCATTTCATTTCATTTATTTCTATTTCATTTCATTTCTATTTCATTTC
ATTTTCATTTCTATTTCTATTTCATTTATTTCTATTTCTATTTCTTATTTTATTTCTATTTCATTTCTATTTCT
ATTTCTATTTTCATTTCATATTTCATTTCTATTTCATTTCTTATTTCTATTTCTTATTTCATTTATTTATTTCA
TTATTTATTTCTATTTATTTCATTTCTATTTCTATTTCATTTTATTTCATTTTATTTATTTCTATTTTCTATTT
ATTTCTTATTTCTATTTCATTTCTATTTTCTATTTCTATTTCTATTTCTTATTTCATTTCTATTTATTTCTATT
TCTATTTATTTCTATTTCTATTATTTATTTATTTTCATTTATTTATTTTCATTTCTTATTTCATTTCATTTCAT
TTCATTTCTATTTCTATTTCTATTTCATTTTTCTATTTTCTATTTCATTTCTTATTTATTTATTTATTTCTATT
TCATTTATTTCTATCATTTTTATTTCTATTTTCTATTTATTTCATTTCTATTTCATTTCTATTTATTTCTATTT
CTATTTTATTTCATTTATTTCATTTCTTATTTATTTATTTATTTCTATTTCTATTTATTTCATTTCTATTTCTA
TTTATTTATTTATTTTTATTTCATTTCTATTTCTTATTTATTTATTTCTATTTCATTTCATTTCTATTTCTGGT
CATTTCTATTTCTTATTTTATTTCTATTTTTATTTCTTATTTATTTCTATTTTCTATTTATTTTATTTTCGCTC
TTTATTTCATTTTTATTTCTATTTCTATTTTATTTCTCTATTTATTTATTTCTATTTCATTTCTATTTCTATTT
ATTTCATTTCATTTCATTTCATTTATTTCTATTTCATTTTCATTTTCATTTCTATTTATTTCTATTTCTATTTA
TTTATTTCTATTTCATTTTATTTCTCTCTATTTCATTTCTACTTATTTCTATTTATTTTCATTTTCATTTCATT
TCATTTCTATTTCATTTCTATTTATTTCTATTTTATTTTATTTATTTCTTATTTGCTTCATTTATTTCTATTTC
ATTTCATTTCATTTCTATTTCATTTATTTATTTCTATTTTCTATTTCTATTTCTATTTTCTTATTTCTATTTCA
TTTCTATTTTTATTTCATTTTTATTGTTTTATTTCATTTCTATTTATTTATTTATTTCATTTTTATTTCTATTT
CATTTTCATTTCTATTTATTTTTATTATTTATTTCATTTCTATTTTTCATTTCTTTTCATTTCATTTATTTCAT
TTCATTTCTTATTTCTATTTATTTCATTTCTATTTATTTCATTTCTTATTTCTATTTCTATTTCATTTATTTCA
TTTCATTTTTATTTCATTTTATTTCATTTCTATTTTATTTCATTTCTATTTCTATTTCTATTTCTATTTCTTAT

TTCCTATTTATTTTCTATTTCTATTTATTTCTATTTCATTTCTATTATTTTATTTATTTTTATTTCATTTCTAT
TTCTTATTTCATTTCTATTTCTTATTTCTGTTTCTTATTTATTTCATTTTATTTCTATTTCATTTATTTATTTA
TTTATTTTCTATTTTCATTTCATTTCTATTTATTTCATTTCTATTTCATTTCATTTCTATTTCTTTTATTTATT
TCATTTCTATTTCATTTCATTTTCATTTCATTTATTTCTTATTTCATTTATTTCTATTTCATTTCTATTTCTTA
TTTCATTTCATTTCTTATTTCATTTCTATTTCATTTCTATTTCTTTATTTCTTATTTCATTTATTTTATTTTTA
TTTATTTCATTTCATTTTATTTATTTCATTTCATTTCTATTTCATTTCTATTTCTTATTTCATTTCTTATTTTA
TTTCATTTCATTTCATTTCTATTTATTTATTTATTTCTATTTCTATTTATTTCTATTTCATTTCTATTATTTCT
ATTTCTATTTCTATTTCATTTTATTTTATTTCATTTCTATTTCTATTTCATTTTATTTATATTTCATTTCATTT
CATTTTATTTCATTTATTTCTTATTTCATTCATTTCATTTCATTTCATTTCATTTCATTTGAGATGGA

h) 1-IV-11_2 (+)
GAAAACTTACTTATACTGTTTATTGGTTCTGTTTTTTATTTATTTTATTTTATTTTATTTATTTTATTTTATTT
TAAACTTTTATTTTATTTTATTTTATTTTTATTTTATTTTATTTTATTTATTTTATTTATTTTATTTTATTTAT
TTTATTTTATTTATTTTATTTTATTTATTTTATTTTTATTTTATTTATTTTATTTTATTTATTTTATTTTATTT
TATTTTATTTTATTTTATTTTATTTTATTTTATTTTATTTTATTTTATTTTATTTTATTTTATTTTATTTTTAT
TTTATTTTTATTTTATTTTATTTATTTATTTTATTTTTATTTTATTTTATTTTATTTTATTTTATTTATTTTTA
TTTTATTTTATTTTATTTTATTTTATTTTATTTTATTTTATTTTATTTTATTTTATTTTATTTTATTTTATTTT
ATTTTATTTTATTTTATTTTATTTTATTTTATTTTATTTTATTTTATTTTATTTATTTTATTTTATTTTATTTT
ATTTTATTTTATTTTATTTTATTTTATTTTTATTTTATTTTATTTTATTTTATTTTATTTTATTTTATTTTAT
TTTATTTTATTTTATTTTATTTATTTTATTTTATTTTATTTTTATTTTTATTTTATTTTATTTATTTTATTTTA
TTTTATTTTATTTTATTTTATTTTATTTTATTTTATTTTATTTTATTTTATTTTATTTTTATTTTATTTTATTT
TTATTTTATTTTATTTTATTTTATTTTATTTATTTATTTTATTTTATTTTTATTTATTTTATTTTATTT
TATTTATTTTATTTTTATTTTATTTATTTTATTTTATTTTTATTTTATTTTATTTTATTTTATTTTTAT
TTTTATTTTATTTTATTTTTATTTTATTTTTATTTTATTTTATTTTTATTTTTATTTTATTTTATTTTATTTTA
TTTTATTTTATTTTATTTTTATTTTATTTTATTTATTTTATTTATTTTATTTTATTTTATTTTATTTTATTTTA
TTTTATTTTATTTTATTTATTTTATTTTATTTTATTTTATTTTATTTTATTTTATTTTATTTTATTTATTTTA
TTTTATTTTATTTTTATTTTTATTTTATTTTATTTTATTTTATTTTATTTTAAATTTTATTTTGATTTTATTT
TTATTTTATTTTATTTAAACATTTTATTTTTATTTTATTTTATTTAATTTTATTTTATTTATTTTATTTTATTT
TATTTTATTTTTATTTTATTTATTTTATTTTTATTTTATTTTATTTTATTTTATTTTATTTTATTTTATTT
TATTTATTTTATTTTATTTTATTTTATTTTGTTTTATTTTATTTTATTTTATTTTATTTTTATTTTATTTTATT
TTATTTTATTTTATTTTATTTTATTTTATTTTATTTATTTTATTTTATTTTATTTTATTTTATTTTATTTTATT
TTATTTTATTTTATTTTATTTTATTTTTATTTTATTTTATTTTATTTTATTTTATTTTATTTTATTTTATTTTT
ATTTTATTTTATTTTATTTTATTTATTTTACTTTTATTTTATTTTATTTTATTTTATTTTATTTTATTTTATTT
TATTTTATTTTATTTTATTTTATTTATTTATTTTATTTTATTTTTATTTTATTTTATTTTATTTTATTTTATTT
TATTTTATTTTATTTTATTTTTATTTTATTTTATTTTATTTTATTTTATTTATTTTATTTTATTTTATTTTTAT
TTTATTTTATTTTATTTTATTTTATTTTATTTTTATTTTATTTTATTTTTATTTTATTTTTATTTTATTTTATT
TTATTTTATTTTATTTTATTTTATTTTATTTTATTTTTATTTTATTTTATTTTATTTTTATTTTATTTT
ATTTATTTTTATTTTATTTTATTTTATTTTTATTTTATTTTATTTATTTTATTTTATTTTTTATTTTATT
TTATTTATTTATTTTATTTTATTTTATTTTATTTTATTTATTTATTTTATTTTATTTTTATTTTATTTTATTTT
ATTTATTTTATTTTATTTTATTTTATTTTATTTTATTTTATTTTATTTTATTTATTTTTATTTTTATTT
TATTTTTATTTATTTTTTATTTTATTTTATTTTATTTTTATTTTATTTTATTTTATTTTATTTTATTTTATTT
TTATTTTATTTTATTTTTATTTTATTTTATTTTATTTTATTTTATTTTTATTTATTTTATTTTTATTTT
ATTTATTTTATTTTATTTTATTTTATTTTATTTATTTTATTTATTTTATTTTATTTTATTTTATTTTTAT
TTTTTATTTTTATTTTATTTTATTTTATTTTATTTTATTTTATTTTATTTTATTTTATTTTATTTTATTTTATT
TTATTTTATTTTATTTTATTTTATTTTATTTTATTTTTATTTTATTTTATTTTATTTTATTTTATTTTATTTTA
TTTTATTTTATTTTATTTTATTTTTATTTTATTTTATTTTATTTTATTTTATTTTTATTTTATTTTATTTTAT
TTTATTTTTTATTTTATTTTTATTTTATTTTATTTTATTTTATTTTTATTTATTTTATTTTATTTTATTTAATT
ACATTTTTTATTTTATTTTATTATTTTATTTATTTTATTTTATTTATTTTATTTATTTTATTTTTATTTTTATTT
TATTTTATTTTTATTTTATTTATTTTATTTTATTTTATTTTATTTTATTTTTATTTTATTTTATTTTTA
TTTTATTTTATTTTATTTTATTTTATTTTATTTTATTTATTTTATTTTATTTTATTTTATTTATTTATTT
TATTTTATTTTTATTTTATTTTATTTTATTTTATTTTATTTTATTTTATTTTATTTTATTTTATTTTATTTTAT
TTTATTTATTTTATTTTATTTTATTTTATTTTATTTTTATTTTATTTTATTTTTATTTTATTTTATTTTATTTT
TATTTTATTTTATTTTATTTTATTTTTATTTTATTTTATTTTATTTTATTTTATTTATTTCATTTCATTTCATT
TCATTTCATTTCATTTCACATTTCATTTCATTTCATTTCATTTTCATTTCATTTCATTTCATTTCATTTCATTT

CATTTCATTTCATTTCATTTCATTTCATTTCATTTTCATTTCATTTCATTTCATTTCATTTCATTTCATTTCAT
TTCATTTCATTTCATTTCATTTCATTTCATTTCATTTCATTTCATTTCGTTTCATTTCATTTCATTTCATTTCA
TTTCATTTCATTTCATTTCATTTCATTTCATTTCATTTCATTTCATTTCATTTTCATTTCATTTCATTT
CATTTCATTTCATTTCATTTCATTTCATTTCATTTCATTTCATTTCATTTCATTTCATTTCATTTCATTTCATT
TCATTCATTTTCATTTCATTTCATTTCATTTCATTTCATTTTCATTTCATTTCATTTCATTTCATTTCATTTCA
TTTTCATTTCATTTCATTTCTATTTCATTTCATTTCATTTCATTTCATTTCATTTTCATTTCATTTCATTTCAT
TTCATTTCATTTTTCATTTCATTTCATTTCATTTCATTTTCATTTCATTTCATTTCATTTCATTTCATTTCATT
TCATTTCATTTCATTTCATTTCATTTTCATTTCATTTCATTTGTTTTCATTTCATTTCATTTCATTCATTTCAT
TTCATTTCATTTCATTTCATTTCATTTCATTTCATTTCATTTCATTTCATTTCATTTTCATTTCATTTCATTTC
ATTTCATTTCATTTCATTTCATTTCATTTTCATTTCATTTCATTTCATTTCATTTCATTTCATTTCATTTCATT
TCATTTCATTTCATTTCATTTCATTTCATTTTCATTTCATTCATTTCATTTCATTTCATTTCATTTCATTTCAT
TTCATTTCATTTTCATTTCATTTCGACCATTTCATTTCATTTTCATTTCATTTCATTTCATTTCATTTCATTTC
ATTTCATTTCATTTCATTTCATTTCATTTCATTCATTTCATTTCATTTCATTTCATTTCATTTTCATTTCATTT
CATTTCATTTCATTTCATTTCATTTCATTTCATTTCATTTCATTTCATTTCATTTCATTTTCATTTCATTTCAT
TTCATTTCATTTCATTTCATTTCATTTCATTTCATTTCATTTCATTTCATTTCATTTCATTTCATTTCATTTCA
TTTCATTTCATTTCATTTCATTTCATTTCATTTCATTTCATTTCATTTCATTTCATTTCATTTCATTTCATTTC
ATTTCATTTCATTTCATTTCATTTCATTTCATTTCATTTCATTTCATTTCATTTCATTTCATTTCATTTCATTT
CATTTCATTTCATTTCATTTCATTTTCATTTCATTTCATTTCATTTCATTTTTCATTTTCATTTCATTTCATTT
CATTTCATTTTCATTCATTTCATTTCATTTTCATTTCATTTCATTTCATTTCATTTCATTTTTCATTTCATTTC
ATTCATTTCATTTCATTTCATTTCATTTCATTTCATTTCATTTTCGAGATGGA

i) 2-IV-16_1 (+)

GAAAACACTTATATTTGTGGTTCTGTTTTTTATTTATTTTATTTTATTTTATTTTATTTATTTTATTTTATTTT
ATTTTTATTTTATTTTATTTTATTTTATTTTATTTTATTTTATTTTTTATTTTATTTTTTATTTTATTTTAATTTATTT
TATTTTATTTTATTTTTATTTATTTTAATTTTATTTTATTTATTTTATTTTATTTTATTTTATTTTATTTTATT
TTATTTTATTTATTTTATTTTATTTTATTTTATTTTATTTTATTTTATTTTATTTTATTTTATTTTTATTTTAT
TTTATTTTTATTTTACATTTTATTTTATTTTATTTTATTTTATTTTATTTTTATTTTTTATTTTATTTTACTTT
ATTTTATTTTATTTATTTTATTTTATTTTATTTTATTTTTATTTTATTTTATTTTATTTTATTTTATTTTATTT
TATTTTATTTTATTTTTATTTATTTTTATTTTATTTTATTTTATTTTATTTTATTTTATTTTTATTTATTTTTA
TTTTTATTTATTTCATTTTATTTTATTTTTATTTTATTTTATTTATTTTATTTTATTTTATTTTATTTTATTTT
ATTTTATTATTTTATTTTTATTTTTATTTTATTTTATTTTATTTTATTTTATTTTATTTTATTTTATTTTTATT
TTATTTTATTTTATTTTATTTTATTTTTATTTTTTATTTTATTTTATTTTATTTTATTTTATTTATTTTTATTT
TATTTTTATTTATTTTTATTTTATTTTATTTTATTTTATTTTATTTTATTTTATTTATTTTATTTTATTTTATT
TTATTTTATTTTTATTTTATTTTATTTTATTTTATTTTATTTATTTTATTTATTTTATTTTATTTTATTTTATT
TTATTTTATTTTATTTATTTTTATTTTATTTTATTTATTTTTATTTATTTTATTTTATTTTATTTTATTTTATT
TTATTTTATTTTTATTTTATTTTATTTTATTTTATTTTATTTTATTTTATTTTTATTTTATTTTATTTTATTTT
ATTTTATTTTATTTTTATTTTTATTTTATTTATTTTATTTTATTTTATTTATTTTATTTTAATTTTTATTTTAT
TTTATTTTATTTTATTTTATTTTATTTTATTTTATTTTAGCTTTTATTTTATTTTATTTTTATTTTATTTTATT
TTTATTTATTTTATTTTATTTTATTTTATTTTATTTTATTTTATTTTATTTTATTTTGTTATTTTATTTTATTT
TTTATTTTATTTATTTTAATTTTATTTTATTTATTTTATTTTATTTTATTTTATTTATTTATTTTATTTTATTT
TATTTTATTTTATTTATTTTATTTATTTATTTTATTTTTATTTTATTTTATTTTATTATTTTATTTATTTTAT
TTTATTTTATTTTTATTTTTATTTTATTTTATTTTTATTTTATTTTATTTTATTTTATTTTATTTTATTTATTT
TATTTTATTTTATTTTATTTTATTTTATTTTATTTTATTTTATTTATTTTTATTTTATTTTATTTTATTTTATT
TTATTTTTATTTTATTTTATTTTATTTTATTTTTATTTTATTTTATTTTATTTTATTTTATTTTATTTTATTTT
ATTTTTATTTTATTTTATTTTATTTTATTTTTATTTTTATTTTATTTTATTTTATTTTATTTTATTTTATTTTA
TTTTATTTTATTTTATTTTTATTTTATTTTATTTATTTTATTTTATTTTTATTTTATTTTATTTTATTTTATTT
TATTTTATTTTATTTTATTTTTAGTATTTTATTTTATTTTATTTTATTTTATTTTATTTATTTTATTTTATTTT
TTCTTTGCATTTTATTTTATTTTATTTTATTTTATTTTTATTTATTTTATTTTATTTTATTTTATTTTTATTTT
ATTTTATTTTATTTTATTTTATTTTATTTTTATTTTTATTTTATTTGTTTTATTTTATTTTATTTTTATTTTAT
TTTTATTTTTTTATTTTATTTTTATTTTTATTTTATTTATTTTATTTTATTTTATTTTATTTTTATTTTATTTTA
CTATTTTTATTTTTATTTTATTTTATTTTATTTTATTTTATTTTATTTTATTTTATTTATTTTATTTTATTTAT
TTTATTTTATTTTATTTTATTATTTTATTTTATTTTTATTTTATTTTTATTTTATTTTATTTTATTTTATTTTA
TTTACTAATTTTACTATTTTATTTATTTTATTTTTATTTTTTATTTTTATTTTATTTTATTTATTTTATTTTATTTTAT
TTTATTTTATTTTATTTATTTTATTTTATTTTATTTTATTTTATTTTATTTTATTTTTATTTTATTTTATTTAT

TTTATTTTATTTTATTTTATTTTATTTTTATTTACTTTATTTATTTTATTTTATTTTATTTTATTTATTTTATT
TTATTTTATTTTATTTTATTTTATTTTATTTTTATTTTTATTTATTTTTATTTTTATTTTATTTTTATTTTATT
TTATTTTAACTTCTTTTTATTTTATTTTTGTTTATTTTATTTTATTTTTTATTTTATTTTATTTTATTTTTATT
TTATTTTATTTTATTTTATTTTATTTTATTTTATTTTATTTTATTTTATTTTATTTTTATTTTTATTTTTATT
TTATTTTATTTTTATTTTATTTTATTTTATTTTATTTTATTTTATTTTATTTTATTTTATTTTATTTTATTTTA
TTTTTATTTTATTTTATTTTATTTTATTTTATTTTATTTTATTTTGTTTTATTTTATTTTATTTTATTTTTAT
TTTATTTTTATTTTTATTTTATTTTATTTTAGTATTTTTATTTTATTTTATTTTATTTTATTTTATTTTATTTT
ATTTTATTTTATTTATTTATTTTTATTTTATTTTATTTTATTTTATTTTATTTTATTTTATTTTATTTTATTTT
ATTTTATTTTATTTTTATTTTATTTTATTTTATTATTTTATTTATTTTATTTTATTTTATTTTTATTTTATTTT
ATTTTATTTTATTTTATTTTATTTTATTTTATTTTTATTTTATTTTTATTTTATTTTATTTTATTTTTATTTTA
TTTTATTTTATTTTATTTTATTTTATTTTATTTTATTTTATTTTATTTATTTTTATTTTATTTTATTTTATTTT
ATTTTATTTTATTTTATTTTATTTTATTTTTTATTTTATTTTATTTTATTTTTTATTTTATTTTTATTTTATTT
TATTTTGTTTATTTTATTTTATTATTTTATTTTATTTTATTTTATTTTTATTTTATTTTTATTATTTTATTTTA
TTTTATTTTATTTATTTTATTTTATTTTATTTTATTTTATTTTTATTTTATTTTATTTTTATTTTATTTTTATT
TTATTTTATTTTTATTTTATTTTATTTTTATTTTATTTTATTTATTTTATTTTATTTTATTTTATTTTATTTT
ATTTTATTTTATTTTATTTTATTTTATTTTATTTTTATTTTATTTTATTTTTATTTTATTTTATTTTATTTTTA
TTTTTATTTTATTTTATTTTTATTTTATTTTATTTTATTTTATTTTATTTTATTTTATTTTATTTTGTTTTATT
TTATTTTTATTTTATTTTATTTTATTTTATTTTATTTTATTTTATTTTATTTTATTTTATTTTATTTTATTTTA
ACTTTTATTTTATTTTATTTTATTTTATTTTATTTTATTTTATTTATTTTTATTTTATTTTATTTTATTTTATT
TTATTTTATTTTATTTTATTTTATTTTATTTTATTTTATTTTATTTTATTTTATTTTATTTTATTTATTTTATT
TTATTTTATTTTATTTTATTTTATTTTATTTTATTTTATTTTATTTTTATTTTATTTTATTTTTATTTTATTTT
ATTTTATTTTATTTATTTATTTTATTTTATTTTATTTTATTTTATTTTATTTTATTTTATTTTATTTTATTTTT
TTATTTTTTATTTTATTTATTTTTATTTTATTTTATTTTATTTTATTTTATTTTATTTTATTTTATTTTATTTT
ATTTTATTTTATTTTTATTTTATTTTATTTTATTTTATTTTATTTTATTTTATTTTATTTATTTTATTTATTTT
ATTTTATTTTATTTTTATTTTATTTTATTTTATTTTATTTTATTTTATTTTATTTTTATTTTATTTTATTTTTAT
TTTATTTTATTTTATTTTATTTTATTTTATTTTTATTTTTATTTTATTTTATTTTATTTTATTTTATTTTTTAT
TTTTATTTTATTTTATTTTATTTTATTTTATTTACTATTTTATTTTATTTTATTTTATTTTATTTTATTTTATT
TTATTTTATTTTATTTTTATTTTATTTTTATTTTATTTTATTTTATTTTTATTTTATTTTATTTTATTTTTATT
TTATTTTATTTTATTTTATTTTATTTTATTTATTTTATTTTATTTTATTTTATTTTATTTTATTTTATTTTATT
TTATTTCTATTTTATTTTATTTTATTTTTTTATTTTATTTTATTTTATTTTATTTTATTTATTTTTTATTTTTAT
TTTTTATTTATTTTATTTATTTTATTTTATTTATTTTATTTTATTTTATTTTATTTCATTTCATTTCATTTCATTTTTC
TTCATTTCATTTTCATTTCATTTCATTTCATTTCATTTCATTTCATTTCATTTCATTTCATTTCATTTCATTTC
ATTTCATTTCATTTCATTTCATTTTTCATTTCATTTCATTTCATTTCATTCATTTCATTTCACATTTCATTTCA
TTTCATTTCATTCATTCATTTCATTTCATTTCATTTCATTTCATTTCATTTCATTTCATTTCATTTCATTTCAT
TTCATTTCATTTCATTTCATTTCATTTCATTTCATTTCAATTTCATTTCATTTCATTTCATTTCATTTCATTTC
ATTTCATTTCATTTCATTTTCATTTTCATTTCATTTCATTTTCATTTCATTTCATTTCATTTCATTTCATTTCA
TTTCATTTCATTCATTTCGATGTTTCATTTTCATTTCATTTCATTTTTCATTTCATTTCATTTCATTTCATTTC
ATTTCATTTCATTTCATTTTCATTTCATTTTCATTCATTTCATTTCATTTCCTTTCATTTTCATTTCATTTCAT
TTCATTTCATTTCATTTCATTTCATTTTCATTTCATTTTCATTTCATTTCATTTCATTTCTGTTTCCATTTTCC
ATTTCATTTTCATTTCATTTCATTTCACATTTCATTTCATTTCATTTCATTTCATTTCATTTTGTTTCATTTCA
TTTTCATTTCGTTTCATTTCATTTCATTTCATTTCATTTCATTTCATTTCGACTTCATTTCATTTCATTTCATT
TCATTTTCATTTCATTTCATTTCATTTCATTTCATTTCATTTTCGAGATGGA

j) 2-IV-9_1
TCAATGCCACCATTCAATTACAACCCGCCGTAAATAAAATAAATAAAATAAATAAAATAAATAAAATAAATAAA
TAAATAAATAAAATAAATAAACTCCGCTCCACGCTCCGCTCCGCTCCCGCTCCATCCGCTCACTCCGCTCCGCT
CCGCTCCCGCTCCGCTCCGCTCCCGCTCCGCTCCACTCCGCTCCGCTCCACTCACTCCCGCTCCACTCACTCCC
GCTCCACTCCACTCCGCTCCACTCCACTCCGCTCCGCTCCACTCCACTCCACTCCACTCTCCACTCCACTCCGC
TCCCGCTCCCGCTCCACTCCCGCTCCACTCCACTCCACTCCACTCCACTCCACTCCATCTCCCACTCCCACTCC
ACTCCCTCCACTCCACTCCCACTCCCACTCCACTCCACTCCACTCCACTCCACTCCACTCCACTCCACTCCGCT
CCACTCCACTCCGCTCCGCTCCACTCCACTCCGCTCCACTCCGCTCTCCCGCTCCACTCCACTCCGCTCCACTC
CACTCCGCTCCACTCCCGCTCCCGCTCCCGCTCCACTCCGCTCCACTCCACTCCACTCCACTCCGCTCACTCAC
TCCACTCCACTCCACTCCACTCACTCCACTCCGCTCCACTCCACTCCGCTCCCTCCACTCCGCTCCACTCCACT
CCGCTCCACTCCCACTCCCGCTCCACTCCACTCACTCCGCTCCACTCCACTCCACTCCCACTCCACTCCCTCCC

ACTCCACTCCACTCCACTCCACTCCACTCCGCTCCCGCTCACTCACTCCACTCCACTCCACTCCACTCCACTCA
CTCCACTCCCGCTCACTCCGCTCCACTCCACTCCACTCCACTCCACTCCACTCCCGCTCCACTCCACTCCACTC
CACTCCACTCCACTCCACTCCACTCCGCTCACTCCGCTCCACTCCACTCCACTCCATCCGCTCCGCTCCACTCA
CTCCGCTCCACTCCACTCCACTCCACTCCACTCACTCCACTCCCTCCACTCCACTCCACTCACTCCACTCCACT
CCACTCCACTCCCGCTCCGCTCCGCTCCGCTCCCGCTCCACTCCACTCCACTCCACTCCACTCCCTCCCGCTCC
ACTCCACTCCACTCCACTCCACTCCTCCACTCCACTCCACTCCACTCCACTCCACTCCACTCCACTCCACTCCA
CTCCACTCCACTCCACTCCACTCCACTCCCTCCCACTCCACTCCACTCCACTCCACTCCGCTCCCACTCCGCTC
CACTCCACTCCACTCCCGCTCACTCCACTCCACTCCCGCACCCTCCACTCACTCCGCTCCACTCCACTCCATCC
GCACTCCACTCCACTCCGCTCACTCCACTCCGCTCACTCCACTCCACTCCACTCCACTCCACTCCACTCCACTC
CACTCCACTCCACTCCACTCCACTCCGCTCCACTCCACTCCACTCCCGCTCCACTCCACTCCACTCCACTCCGC
TCCACTCCGCTCCACTCCACTCCGCTCCCTCCCTCCACTCCACTCCACTCCCTCCACTCCACTCCGCTCCACTC
CGCGCTCCGCTCCACTCCACTCCACTCCACTCCACTCCACTCCACTCCACTCCACTCCACTCCGCTCCACTCCA
CTCACTCCACTCCGCTCCACTCCGCTCCACTCCGCTCCACTCCACTCCACTCCACTCCACTCCACTCCCACTCC
ACTCCACTCCGCTCCACTCCACTCCGCTCCACTCCGCTCCACTCCACTCCGCTCCGCTCCACTCCCTCCCGCCC
GCTCCACTCCGCTCCGCTCCGCTCCACTCCGCTCCGCTCCGCTCCGCTCCACTCCCCTCCGCTCCGCTCCGCTC
ACTCCGCTCCGCTCCGCTCCCGCTCCGCTCCCGCTCCAGCTCCGCTCCGCTCCGCTCCGCTCCGCTCCGCTCCG
CTCCATCCCTCCCGCTCCATCCGCTCCCATCCCGCTCCATCCGCTCCGCTCCGCTCCGCTCCATCCATCCCGCT
CACCGCTCCGCTCCGTCATCATCGCTCCGTAAATAAAATAAATAAAATCATCCGTCGTCAAATAAAACAAATAA
AATAAAATAAATAAAATCGTCATCAAATAAAATAAATAAAATAAAATAAAATAAAATAAAATAAATCAAATCAA
AATAAATCAAATCATCAACTCCGCTCCACGCTCCGCTCCGCTCCGCTCCACTCCACTCCGCTCCACTCCACTCC
ACTCCACTCCACTCCCTCCCACTCCACTCCACTCCGCTCCACTCCGCTCCACTCCACTCCACTCCACTCCCTCC
ACTCCACTCCACTCACTCCACTCTCCACTCCCGCTCCGCTCCACTCCACTCCCTCCACTCCGCTCCACTCCACT
CCACTCCCGCTCCACTCCACTCCACTCCACTCCACTCCCTCCACTCCACTCCACTCCACTCCACTCCGCTCCCA
CTCCACTCCACTCCACTCCACTCCGCTCCACTCCACTCACTCCACTCCACTCCACTCCGCTCACTCCACTCCAC
TCCGCTCACTCCACTCCACTCCGCTCCACTCCACTCCACTCCACTCCACTCCACTCCACTCCGCTCACTCCCGC
TCCGCCTCCACTCCACTCCACTCCACTCCACTCCACTCCGCTCACTCCACTCCACGCTCCACTCCACTCACTCC
ACTCCGCTCCGCTCCACTCCACTCCACTCCACTCACTCCACTCCCACTCCACTCCACTCCACTCCACTCCACTC
CACTCCACTCCACTCCACTCCACTCTCCACTCCACTCCATCACTCCACTCCACTCCCGCTCCACTCCACTCCAC
TCCACTCCGCTCCACTCCGCTCCACTCCCGCTCCACTCCCACTCCGCACTCACTCCGCTCCACTCGCTCCACTC
CACTCCACTCCCGCTCCACTCCGCTCCACTCCACTCACTCCACTCCACTCCACTCCACTCCACTCCACTCACTC
CCTCCACTCCGCTCCACTCCACTCCCGCTCCGCTCCACTCCACTCCACTCCACTCCACTCCACTCCGCTTTCCC
TCCACTCCCGCTCCCGCTCCACTCCACTCCGCTCCACTCCACTCCACTCCACTCCGCTCCGCTCCCGCTCCACT
CCACTCCACTCCACTCCGCTCCACTCCACTCCACTCACTCCACTCACTCACTCCACTCCACTCCACTCCACTCC
ACTCCACTCCACTCCGCTCCACTCCCGCTCCGCTCCCGCTCCACTCCCGCTCCACTCCGCTCACTCCACTCCGC
TCACTCCACTCCACTCCACTCCGCTCCCGCTCACTCCACTCACTCCGCTCCACTCCGCTCCACTCCCCGCTCCG
CTCCCGCTCCGCTCCGCTCCGCTCCATCGCTCCGCTCCGCTCCGCTCCGCTCGCTCCACTTTCAATATAAAATG
TTCAAAAAGAAATGAAGTAATCACAATTTCATTAGTTTATGTTGCTCCATCGAATAAATTTCTAAAAGCAATTC
GCTCAAAATCGCATTTTTAAAATAACGATTTCACAAATAGAACATTTTAATGGTTTTTAGCATCTTGCTATATC
ATTTTAATCCTTTAAGTGTTATTCAACAAAATTCAATTATAAAATGTCAGAACTTCGATTGCAAAACATGAAAT
AAATTCATTTCATGACTAATAATTCATAAAATAAAGCTCGTCAAGTTCCACCAAATTATCTCGTAGTTCATCCT
GGGGAGTGTCAAAATAAATTTCAACGATGGACTTAGCCAGTATACACTCGCCAAAATAATCAATTGGGTCGACT
CTGACAGACCTGGATCTTACCTTGATATCCTGGACGGTGCCAATCATACTAACCATCGGTTCCTCATCTAAAAT
GAAATGCAAACCTGATGTGTAAGGGGTAATAAATGCTCTCAAGTGTTCATCGCTTAGCCCGATTGGAGTCTTCA
ACTTGTATTATTTTAACAAGAGCAACAGTGTTCAATAAATGTTATGTATACATATCAGATTATAATTTACAAAA
TATGTTCGCCGCAATTGGTGAAAAATATTCATGAGTTATACGATAGCTTCGAGCGCAATTCCTAAGATCTGGTC
TATCCCTATAAAGTATCTAAATGAAACAGTATCTTC

k) 2-IV-9_2

CTGTTATGCCTGCTTCCGTTCAGTTACACGTATTGCTATTTACATGATCTCTCTGGGTCAAATGTGAGATGCCT
CCTTCTGAAGGGAAAGTCAAGCTCAGCAAACAGGTGCCCCAACTCTGGCATCTCAAATGTTCACGGATGCCCAA
TATAGCATTGTCTGGAGAACAATATCAGTCACGACCCACTGGGCCACACCTTTCCCCAATTTAAGAGAAGGGGT
ATTCACACCATCAAGGAAGCAATTGAATTTCATTATGGAGTTGACATCTTTTTCTGAATAAATGCTGTTTATAT
TCATGTTCTTGCTGCTCAGCATGTCTGGGGAATGTCTTTAAGAACTGTCACAATTTTTCGATGATTCTGACTTA
AAGTTGGCCGTGGTAGCATGGCTGTAGATAACGGTTCTTGAAGAAAGGGACATTGTGCTTGTAAATCCAACTTT

TAATGGCACAATCTGGAATGTCTCAGGTTGAAGGGTTTTGATTTTATTATGTTGGCAGCCCGAGCAAATCAGGT
CTTGCAACAGTAACCTCAGTTCTTCCAACCGAGGGCATAAAGGCACATGTGGGACTGAGACAGAGGCTCAGTAG
TGAAGAGTTTGATAAAGGTTTTACAGCAGGGTGAAAGAAGTAAATTACACTTGGAGGTCAGAGGAGTTCTTGGG
GGTTTACAATGGCATGGTTCAGGATCTTTGCGTCTCAGTTCTTCCTTGGGGTGTTTGTCTGCATGTGCAGTGGC
ATTGCTAACATGGAGGGCTGCATGTCTGAGTTAGCAGGGTGTGCTTTTTCCTTACCAGGTCATAGACCAGTTCT
GCCAATCCACTTGGGATTCTACCTGATGACCTTTCCTGGTTGGCTGTGACCAAGTGCTGACAGAGGACAGTTTA
ACAACGCAGTTGCGCCCCACCATTTCCTAAAGTACTGGTTGCTCTGGTTTGCTCCCAGATTCAATTGAGATTGA
TGTGGCTTCATTCATGAAACCAAAATTCTCACCATTCATTTTCACGTATACAGCAAGGCAAGAAATATTCCGTT
CTAGGACAGATGGATAACAAATCTCAGTAAATGAGTCAGGCAAAAATCAAACCATTCACTTGGCTGTGGAGTCA
ATTTGTTATGTGCAAATATATTCTGGGCACGGTGGGAACCAAATAAAAGCATTTAAACAACTTTGTAATGCTAC
AGAGAAATGTTGGCTGCATGACAACGCTGAAGGATGTAAGATTCTATTGTTTGTGGCTAAATAACATTCAATGT
TTTTGTGATTTTTTTAACTTGGTTGAGAATATTTTTTCAAAAAGAATGATGGCCAGAGTTCTACCCAAAAGTAT
TTATGCAGGGGGTCAGAACCAGTGATTAAAAGTCCTATAACCCCAGCAGTTGTTTTATTATGTTATGCATGATG
CAGGATATTGCACATGGATTACTACTATGCATTGAATCACCACTCTAATGGTTTGGGTTTCTGTTTGTTTCTCT
TCAGCTCAGTTCAATGTGGATCAATGATCGGATCAGGCCAATGTTGGGTGTTCTTCTCACTTTTTGGGATGAGA
ACAGGATTGTGTTCAAACGGATGAGTTCGTTTTCATTTCGTTTTCATTTCTCATTTCGATTTCGTTTTCGATTT
CAGGTTTTCGTTTCGTTTCGGTTTCGTTTCGTTTCTGGTTTTCGTTTCGTTTCGTTTCGTTTCGTTTCGTTTCT
GGATTTCAGGTTTCGTTTTTCTGGATTCGTTTTCGGTTTGGGACTTGGTTTTTTTTCAGATGGTTTTTTTTTTT
TTTCGTTTTTTTTTTTTTTTGAACACATCTTGGTTTTCATTTCATTTCATTTCATTTTTCATTTCATTCATTTC
ATTTCACATTTCATTTCTTTCAATTTTCAGTTTCATTTTTTCTGTTTCATTTCATTTCATTTCATTTTTTGTTT
CTGGAACTTTCATTTTCATTTTCAATTTCATTTCATTTCATTTCGTTTCATTTCATTTCATTTCATTTCATTTC
GTTTCGACAATTTCATTTCATTTCATTTTTTTCGATTTTTCATTCCTCATTTCGGAGGTTTTTCATTTCATTTC
ATTTCATTTTCATTTCATTTTTTCATTTCATTTCATTTCGATTTCATTTCATTTTCATTTCATTTTCGATTTCA
TTTCATTCATTCATTTCATTTCATTTCATTTTTTCATTTCATTTCATTTCCGATTTCATTTCATTTCATTTTTT
CATTTCTGTTTCATTTCATTTCATTTCATTTTCATTTCATTTCATTTCATTTCGTTTCATTTTCCTTCAATTTC
ATTTCTGTTTTCATTTCGATTTTTTCTCATTTCATTTCATTTCGATTTCGTTTCATTTCATTCATTTCTGTTTG
TTTCATTTCATTCATTTGTTTCATTTCATTTCGTTTCATTTCATTTTTCATTTTTCATTTCGACTTTCATTTCA
GATTCATTTCATTTCGTTTCATTTCTGGTTTCATTTTCGTTTCATTTCTTCCGATTTCATTTCGGTTTCATTTC
ATTTCATTTTTCATTTCATTTCATTTCATTTCATTTCATTTCATTTCGTTTTTCATTTTCATTTCATTTCATTT
CATTTCAGACGTTTCGTTTCATTTCATTTCATTTCATTTCATTTCATTTCATTCGTTTTCATTTCGATTTCATT
TCATTTTGTTTCATTTCAATTTCATTTCATTTCATTTTCATTTCATTTCATTTCATTTCATTTCATTTCTGATT
GGGAGTTTTTCGATTTCGATTTCGTTTCATTTCGATTTCGTTTTTCGTTTTCGGTTTTTCGTTTCTGTTTCATT
TCATTTTTCATTTCATTTCATTTCGATTTCATTTCTGTTTTTTCATTTCATTTCATTTCATTTCGTTTTCTGCA
TTTCATTTCATTTTCATTCATGGAACTTTTTCATTTCGGTGACATTTCATTTCATTTCATTTCGGTTTCTGTTT
CATTTTCATTTCATTTTCATTTCATTTCATTTTCATTTTCATTTCATTTCATTTCAATTTTCGATTTTCATTTC
GACATTTCATTTTTCGTTTTCGAATTTCATTTCATTTCGTTTTCATTTTTCATTTCGTTTCAAATTTCATTTCA
TTTCATTTCATTTCATTTCATTTTCTGTTTTCATTTCATTTCACAACGACTTCATTTCATTTCATTTCATTTCA
TTTCACCATTTCATTTTCGGAAGACTTTCATTTCGTTTCATTTCATTTCATTTCGATTTTTCGGTTTCATTTCA
TTTTGTTTCATTTCATTTTCATTTCATTTTCATTTTGGAACTTTCGACTTTTTCATTTCATTTTCATTCCATTT
CATTTCATTTTCATTTCATTTCATTTCGTTTTCATTTCATTTCATTTCGTTTTTCATTTCACGTTTCATTTCGT
TTTCATTTCATTCGTTTCATTTCATTTCGTTTCATTCGTTTTTTCGATTTTGTTTCGTTTCATTTCGTTTTCA
TTCGATTTCTGGAACTTCGTTTCGTTTCATTTCGTTTCGTTTCATTTCGTTTTCTTTCGTTTTTCGTTTCTGGA
CTTTCATTTCATTCATTTCATTTCATTTCGTTTTCATTTCATTTCGTTTCATTTCGTTTCATTTCATTTTCATT
TCGTTTCATAATTTCATTTTTTCATTTCATTTCAATTTTCATTTTCATTTCATTTCTCATTTCATTTCGATTTC
ATTTCGATTTCATTTCATTTCATTTCTAATTTCGTTTTTACGTTTCATTTCATTTCATTTCGTTTCATTTCGTT
TTCATTTCGAATTTCATTTCATTTCATTTCTAGTTTCATTTCATTTCGTTTTCATTTTCATTTTCATTTTCATT
TCATTTCATTTCATTTCATTTCGATTCGATGTTTCATTCATTTCATTTCATTTCATTCATTTCATTTCATTTCA
TTTTCTGTTTTCATTTCATTTCATTTCATTTCATTTCTGTTTCATTTCATTTTCATTTCGTTTCATTTCATTTC
ATTGTTTTCATTTCATTTCATTTCATTTCATTTCATTTCAATTTTTATTTTCCGAGGATGGAGTCTTGCCCTGT
TGCCCAGGCCTTGATCTCTGCTCACCAACCTCCACCTCCCGGGTTCAAGGCGATTCTCTACTTGCTCCACAGCC
TCCTGGTGGCTGGGATTGGGGAGGCGCCACCTCCCTCTTAATTTTTTGTGTTTTTTAGTAGAGATGCTGGGGGT
TTAGCCATGTTAGCCAGGGACTGGTCTCAAACTCCTGACCTCAGGTGATCTGTCCGCCTCAACCTCAGGTTCTG
GCGTGGGCAGCTGGTTCCTGTTTTCTCTGTCCTTTAATGATATTCTGAACCATGAGATTAAAGTGTTACCAAAA
CATCAACCGTGCGCCAAAAGTGGGAGATGCTTGTTGCTTGCCTCTGATTTTCTTGATGGTCTGATGCTCAGAAT

GTTGTATGCATTTGTAGTAATGCTTAGTTCCGACATGGCTGTTTCTAATTGATGTTTAGCATAGATTTACTAGA
TCTCTAAGTTTCTTCAGTATTAAATTAATTTTATTAAAATCAACAGGTTTATAATCCATTCTCATGGGGATGGA
TAAATCATTCTTACTATTATTGTAATATTGTTGTATGAGTGCAGATGTGTTATGTCAACTGCTATCCATCCTAA
ATGACATTTTTAAACTTCAGAGCTATAAGGAACCTTGAACATTTGCCCTCCTCATTTATCGGTTAGGAGTCCGA
TCCTGTAATGCACTGAGATTTCTCAGTTTCAGAGGAAAAAGTCTATCCTTTGGTCAAGGATATACTTAGGAAAA
GCAATTACAATATTGGAATAGATGTTGCAAGAAGTGTTGATGTGCAGTTCTCAAAGATAATATTAAACTTTCAT
TTGTCATAGAATGCCCATTTTACCGGCTCAGTGATATAAAGCTGAGGGTTTTTTTTTCTTTTCCGTTTTGCTA
AATTATATACTTGTTAATTTTGATACAGGAAGCAGCTGTTCTTTGTGCTTTACGGACATATATTCATTCGAAAG
TGTGAAGGCTAAAGAGTATTATGGTTGATCTGTGTTCTATATCACTATGACAAAAGTTGTGAAAACTTAAATTT
GGAAGAAATGAGTTAAAAGGATAGAAGTTGTCAGAATCAGACAGCTGGTTTTTCTCTCTTTCTAGACCCACACA
GTCTTCTATTTTGTTTCAGAGATGTCTTATATAAATGTTTCTTACCACGCATTACCTTTCACTGTTTTGGCATG
CTTGACCAAGGTGTACGGCCATTTAGTTCGTTAAATGTTTACTGAGGGACCTGTGCCGGAGCCAGGCACTATTC
TGGGCAAGGTGGTTATTAGCTGGGGGTTCTTATAAGCGGCTGTGGAGAACAGTGGATTACGGGAGGGATAAGAG
GTCTGGGATATGAGGGGGCATGGGTTTGCTGGGCTGGGTCGCTGCTTTTGATGCAGCACAGAGGGTCGCAGTTT
AAAACGATAAGGAGGAGGACTCACTTGAGGAGGAACAAGGAGGGGGATTAATGATAAGCCAGGATATACTGGTT
CCCCAGGAAGGAGGAACATAGCCACAACCTGTGAAGTGTCTAAGATGGAATGGGAGCTATGGAGGGTTTGAGCT
GACATAGGTGTCTTGATGGCCCAACTTGTTGTGGGTTCCTCATCATATTTCAGTTCGTCGGTGTTATGGTGTTT
GGACAGTTAAAGTTATGTCAAATATGTAGATATA

l) 2-IV-9_3

CTCGACAGTTGTGTGCTGGCGTCTTAGCGTCAGGTTCCGTGAAGGTTCCTTTTCATTTCGTTTGTTTCATTTCA
TTTTCATTTCATTTCTGTTTCATTTCATTTGTTTCATTTCATTCATTTCATTTCATTTCATTTCATTTCATTTC
ATTCATTTCATTTCATTTCTGTTCATTTTCATTTCTGTTTCATTTCATTTCATTTCATTTCATTTCATTTCTGT
TTCTTCATTTCATTTCATTTCATTGTTTCTTTCATTTCGACTTTCTGACTATTTCATTTCGATGTTTCGGTTCA
TTTCTATTTCATTTCATTCATTTCATTTCATTTCATTTCATTTCATTTCATTTCATTTCATTTCATTTCATTTC
ATTTCATTTCGTTTTCATTCTGTTTCATTTCATTTCATTTCATTTCATTTCAACTTTGTTTCATTTCAACTTTC
ATTTCATTTCATTTCATTTCATTTCATTTCATTTCATTTCATTTCATTTCATTTCATTTCATTTCATTTCATTT
CATTTCATTTCATTTCATTTCATTTTCATTTCGACTTTGTTTCATTTCATTTCATTTCATTTCATTTCATTTCA
TTTCATTTTCTGTTTCATTTCATTTCATTTCATTTCATTTCATTTCATTTCATTTCATTTCATTTCATTTGTTT
CATTTCATTCATTTCATTTCATTTCATTTCATTTCATTTCATTTCATTTCATTTCATTCATTTCATTTCACATT
TCATTTCGACTTTCATTTCATTTCATTTCATTTCATTCATTTCATTTTCATTTCATTTCATTTCATTTCATTTC
ATTTCATTTCATTTCTGTTCGACTTTCATTCTTTTCATTTCATTTCTTTGTTTTCATTTCATTTTCATTTCATT
TTTCATTTCTTTTCATTTCATTTCTTTCATTTCATTTCATTTCATTTCATTTCATTTCATTTCATTTCTTTCAT
TTCATTTCATTTCATTTCATTTCATTCATTTCATTTCATTTCATTTCATTTCATTTCATTTCATTTCATTTCAA
TTTCATTTTCATTTCATTTCATTTCATTTCATTTCATTTCATTTCATTTCATTTCAATTTCATTTCGAC
TTTGTTTCATTTCTTTCATTTCATTTCATTTCATTTCATTTCATTTCATTTCATTTCATTCATTTCATTTCATT
TCTTTTCATTTCATTTCATTTCATTTCATTTCATTTCATTTCATTTCATTTCATTTCATTTCATTTCATTTCAT
TTTCATTTCATTTCATTTCATTTCATTTCATTTCATTTCGTTTCATTCATTTCGCTTTCATTTCATTTCATTTC
ATTTCATTTCATTTCTGTTTCTTTCATTTCATTTCATTTCATTTCATTTCATTTCATTTCATTTCATTTCATTT
CATTTCATTTCATTTCATTTCATTTCATTTCATTTCATTTCATTTCATTTCATTTCATTTCGACTTTCATTTCC
ATTTCATTTCATTTCATTTCATTTCATTCATTTCATTTCATTTCATTTCATTTCTTTTTGTTTCATTCATTCAT
TTCATTTCATTTCATTTCTGTTTTCATTTCATTTCATTTCATTTCATTTCATTTCTATTTCGACTTTTCATTTC
ATTTCATTTCATTTCATTTCATTTCATTTCATTTCTTGTTTCATTTCATTTCATTTCATTTCTTTCATTTCATT
TGACTTCATTTCATTTCATTTGTTTCATTTCATTTCATTTCATTTCATTTCATTTCATTTCATTTCATTTCATT
CATTTCATTTCATTTCATTTCATTTCATTTCATTTTGTTTCATTTCATTTATTTCATTTCATTTCATTTCATTT
CGACTTTCATTTCATTTCATTTCATTTCATTTCATTTCATTTCACATTTCATTTCTTCATTTCATTTTCATTTC
ATTTCATTTTTGTTTCATTTCATTTCATTTCATTTCATTTCATTTCATTTCATTTCTGTTTCATTTCATTTCAT
TTCATTTCATTTCATTTCATTTCAACATTTCATTTCACATTTCGACTTCTGTTTTGTTTCATTTCATTTCATTT
CATTTCATTTCATTTTGTTCATTTCATTTCATTTCATTCATTTCATTTCATTTTGTTTCATTTCATTTCATTTC
ATTTCATTTGTTTCATTTCATTTCATTTCATTTCATTTCATTTCATTTCATTTCATTTCATTTCATTTCATTTT
CATTTCATTTCATTTCTTTCATTTAATTTCTTTCATTTCATTTCATTTCATTTCATTTCATTTCATTTCTGACT
TCATTTCATTTCATTTCATTTCATTTCATTTCATTTCATTTCATTTCTGTTTCATTTCATTTCATTTCATTTCA
TTTCATTTCATTTCATTTCTTTTCGGCTGTTTGTTTCATTTCATTTCATTTCATTTCATTTCATTTCGACATTT
TCTGTTTCATTTCATTTCATTTCATTTCATTTCATTTCATTTCATTTTGTTTATTTCATTTCATTTCATTTCAT

TTCATTTCATTTCATTTCATTTCATTTCAAAACTTCATTTCATTTCACATTTCATTTCAATTTTCATTTCATTT
CATTTCATTTCATTTCACAATTTCATTTCATTTCATTTCATTTCATTTCATTTTCATTTCATTTCATTTCATTT
CATTTCATTTCATTTCATTTCATTTCATTTCATTCATTTCGACTTCATTTCGACTTTGTTTGTTTCATTTCATT
TCATTTCATTTCATTTCATTTCTGTTTCATTTCATTTCATTTCATTTCATTTCATTTCATTTCATTTCGGCTTC
ATTTCATTTCATTTCATTTCATTTCATTTCATTTCATTTCATTTCATTTCATTTCATTTCTGTTCATTTCATTT
CTGTTTCATTTCATTTCATTTCATTTCATTTCATTTCATTTCATTTCATTTCATTTCATTTCATTTCATTTCAT
TTCATTTCATTTCATTTCATTTCATTTCATTTCATTTCATTTCATTTCCTTCATTTCATTTCATTTCAT
TTCATTTCATTTCATTTCATTTGTTTCATTTCATTTCATTTCATTTCATTTCATTTCATTTCATTTCATTTTCA
TTTCATTTCATTTCATTTTGTTTCATTTCATTCATTTCATTTCATTTCATTTCATTTCATTTCATTTCATTTCA
TTTCGATTCATTTTTCCATTTCATTTTCATTTCATTTCATTTCATTTCATTTCATTTTCATTTCATTTTCATTT
CATTTCATTTTGTTCATTTCATTTCATTTCATTTCTGTTCATTTTGTTTCATTTCATTTCATTTCATTTCATTT
CATTTCATTTCATTTCATTTCATTTCATTTCATTTCTGTTTCATTTCATTTCCATTTCATTTCTGTTTCATTTC
ATTTCATTTCATTTCATTTCATTTCACCATTTCATTTCTGTTTTCATTTCTATTTCATTCATTTCATTTCTGTT
TCATTTTCGACTTTGTTTCATTTCATTTCATTTCATTTCATTTCATTTCATTTCATTCATTTCATTTCATTTCA
TTCTGTTTCATTTCATTTCATTTCATTTCATTTCATTTCATTTCATTTCATTTCATTTCATTTCATTTCATTTC
ATTTCATTTCATTTCATTTCATTTCTATTTCGACTTTCATTTCATTTCATTTCATTTCATTTTCATTTCATTTC
ATTTCATTTCATTCATTTCATTTCATTTCGACTTCTTCATTTCATTTCATTTCATTTCATTTCATTTTGTTTCA
TTTCATTTCTGTTTCATTTCATTTCATTTCATTTCATTCATTTCATTTCATTTCATTTCATTTCATTTCATTTC
ATTTCATTTCATTTCATTTCATTTCATTTTCATTTCATTTCATTTCATTTCATTTCATTTCATTTCATTTCATT
TCATTTCATTTCATTCATTTCATTTCTGACTTTCATTTCATTTCATTTCATTTCATTCATTCATTTTCATTTCA
TTTCATTTCATTTCATTTCATTTCATTTCATTTCATTTCATTTCATTTCATTTCATTTCGACTTTTCATTTCAT
TTCATTTCATTTCATTTCATTTCATTTCATTTCATTTCATTTCATTTCATTTCTGACTTTCATTTCATTTCTTG
TTTCATTTCATTTCATTTCATTTCATTTCATTTCATTTCATTTCATTTCATTTCATTTCATTTCATTTCATTTC
CATTTCATTTCATTTCACATTCATTCATTTCATTTCATTGTTTCATTTCATTTCATTTCATTTCGACTTTCATT
TCATTTCATTTCATTTCATTTCATTTCATTTCATTTCATTTCATTTCATTTCATTTCATTTCATTTCATTTCAT
TTCATTTCATTTCATTTCTTTCATTTCGACTTTGTTTCATTTCATTTCATTTCATTTCATTTCATTTCATTTCA
TTTCATTTCATTTCATTTCTGTTTCATTTCTTTCATTTCATTTCATTTTCATTTTGTTTCATTTCATTTCATTT
CTGTTTCATTTCATTTCATTTCTGTTTCATTTCTGTTCAATTTCATTTCATTTCATTTCATTTCATTTCATTTT
ATTTCATTTCATTTCATTTCATTTCATTTCATTTTGTTTCATTTCATTTCATTTCATTTCATTTCATTTCATTT
CATTTTCATTTCATTTCATTTCATTTCATTTCATTTCGTTCATTTCATTTCACATTTCATTTCATTTCATTTCA
TTTCACTGTTTGTTTCATTTCATTTCATTTCATTTCATTTCATTTCATTTCATTTCATTTCATTTCATTTCATT
TCATTTCATTTCATTTCATTTCCTTTTCATTTCATTTCATTTCATTTCATTTCATTTCATTTCATTTCATTTCA
TTTCATTTCAAATATTGTTCATTTCAATTTCATTTCATTTCATTTCATTTCATTTCATTTCATTTCATTTCATT
TCATTTCATTTCATTTCATTTCATTTCATTTCATTTCATTTCATTTTGTTTTGTTTTGTTTCATTTCATTTTCA
TTTCATTTCATTTTGTTTATTTTGTTTTCGAGATGAGTCTTGCCCTGTTGCCCAGGCTTTGAAGTGCAGCTCAC
CCTCCACCTCCCCAGGTTCAGCGATTCTCTGTCAGCTGGGGTAGGCCAATTTTGTGTTTTTGGTAGAGATGAGG
TTTCGCCATGTTGGCAGACTGGTCAAACTCACAACCTCAGGTGATCATCTCAACCTCCCAAAATGCTGGATTCT
GGCCAGGCACCGTTCCTGGCAGTTCTCTTATGTTTTATTATTCTGAACCAAAAACACATCAACGTGCACTTAGT
AGTCTTCCAAAAATAACACATTGCTTTCTGATTAGTGCCATATATGATGGTCCCCAGATGTTGTGCATTTGTGT
AATATTGGTCCGACATGGCTGTTTCTGTACTAATTGTTTGCGTTTAGATCTCTAAGTTCTTCGGTATTAAATTA
ATTTTTGTAAATCAACAGGTTTTATGTCCATTCTCATAGCCCACAGGGATGGATAAATCATTCTTACTGTGTAT
GTTGTTTGTATGAGTGCAGATGTTATGTCAACACTGTCCATCTGACATTTTTTGACTTCCAGGCTTAAAATTAG
ACATTTCTCTCATTGGGTTGAGTCGATGTATTCTTTAATTTCAGAGAAAAAAGAGTCTGTCCTTTTGGTCAGGA
TATGCTTTTTTTAAAGCAGTACAATGAATAGATGTTTTTAGTGTTGATATTGCAGTTCTCAAATAATAATACAT
TTCAGTGATATAAAGCTGAGGATTTTTTTCTTTTCCATTTTCTTGCTTGTATTGCTTTTAACATATTACAAGTC
TTAATTTTTACCAGAGCCATGTTCTTTAACTGCAGGTTATATTCATTGAAAAATTATTGGCTTTAAAGTATTAT
GGTTGATCATGTTCTATCTTTCTTAAATTTGAATGAAAGATTTGTCAATCAACAACTGAATTCTCTTTCTGAAC
CCACAGTGTTTGTTTCCAGAATGTCTGCATGTTTCTTACATTACCTTTCTGTTTTTGTGCTTTGACAGAGTCTT
ACATGCATTTGACCGTTATTTTTGTGGAGACCTGTTATTCATGCATTGGGCTGGGCAGGAACAATATGAAAATC
TTTTCATAGAGGCTTGTAGTTATTGGCTTAAAGACAAAATAAACATGTAGGAGGTATATGTAACTGAAGGTTAT
AAAGCGGCTGTGGAGGAGACAGTGGTGGGAGGAATAAAGAAGTGCCGGGATATGAGGGGAGGCATGGAGTTGCT
TTTTTTTTTTTTTGAGGCAGTGTGCTCTGTCTGCCAGACTGGGTCTGATGGCGCGATCTTGGCTCACTAGAGA
TTAAACAGTTTTAAATAGCAATGGAAGGCCTCTGAGGAAAGGAGCAGAAGGAGTTAGCCATACAGTCTCTGGGC
AGAGCATTCCCAGGCAGGGGAACATGGCAGATGGAGCAGCCTGGAGTGTTGACGTCACCAGGAGAGCAGTGTGA

GTTGGAGAAGTAAGTATAATGAGGTCAGAAGATGTAAGATGATGAGGGAGAGGCATGGCAGGACTTCATTGACT
TTATGAGATGAGCTATGGGAGGGTTTTGAACTGGCGTGAGACATAAATTGATATGCAGCCAACTTTCTGTATTT
GAGTTTGCGTCTATTAGTTCATCCAATGTCGGATGGGCAGAAAAAAAAATAGATGATTATTGCTGAACATGCTTG
CAACCTTTTTTTCTTGTTATTCATAAAGCAATGCAATTATGATGACTGTAACATTTATGTTATAATTCTGGTAA
TCTAGAGGATATTTAGTTGACTCTTGGGTTTTTGAACTACATGGGTCCTTCTTTTTTCTTCAATGAATATACCA
ATTTGCAGATGAACCTTCCAGAAATAGTGAAGAGATTAAAGTTGTCATTGATACAAAATATACTGATGCTTTTC
ATAACCTTAGATCTGTCATGAATGTTATCAAAACAGGCATTCTGGTTGAAGAGATGTAAAAAGATACGGTATTA
AATCATAACTGTAAATTAACCATGGTGCATACTGTGCTGCAACAGCCAGGTGGCCACCTTCCGTTTGCTTTGTT
GTTGTGGGACTCCATGATGTTGCTGATGAGCTCATCTCTCAGTAAATTATTGTATGGTATCTCTCTGGTTCTCT
TGTATTTTCATTTGGTGCGATTACCTGAATAACATGATGGAGTCCATAGCGAGGTGCCCTGAAAGTGATGCAGT
GTCAGGAAGCTGGGCAGGCATTATGAAAGAAAAATTCAGATTGCTTCTTGCCCATGGGTTGAGTTTAACAACCG
GGCCATTTCGAATGATTTATCTTATGGGCAGGTATCAATAAGCCTTAATTTCAGTGCTTAGCCAATTTCCTTCA
GTTTTTCTTGTTCTAGCTTTAATAAGTATATATACAATACCAATACATGCAGCATATAATACAGTCATGGCCAA
TACAGTTTATTCTTGGCATGATTTTTTGTTTTTTTCTTACAGTTCGTCTTTTTTAAATTTCTTCAATATATTTA
TTGAAGAAAATCGATATGAAGTGGACCCATGTAGTTCAAACATGATGTTCCTTTGGTCTGTGCTTTAATAATCT
TTCACCAATACCTGGTATATTAATACAAAGGTCGGTGGTCAGATTGGCAGGCAGATCCTGGGGTCAGGTTGAGA
CCAGCCTGGACAACATGGCGAACCCAATAGCTGGATGGTGGCAGGTGCCATGATCCAGGCTGAACAGATACATT
GGAGCTTTGATCTTGAATCGCGCCACTCAGCAGACTCGTCTCAATGTATTAATCTATTTATGTTTGACGATATG
TTATGGGAGACTTCTGGTCACCAATGACTGTTAGTAGTTCCGTTTCGAGGTCAAAGTTGTATTGTTTTTTTTTG
AGACAGAGTTTTGCTCTTGACTGAAATTCCAATTGGCACCATCTCAGCATTGCAACCTCCAGGTTCCAGCCTCA
GCTGGATTACAGGCATGCCACAGCCTGTATTTTTCGTAGATGGGTTTCATCATGACCCAGCAGACTCCTGACCT
CAGGTGATCGTCTGCCTCCCAGTTATAGGCTTTAATTTTTGATTGTGGGGAGTCAGTTACTAACTTTCTTGTTC
AGGGGACTCTGTATATGGAAGGATGTGCATAGTTGCCCAAATACTATGCTGTTTTGCCAGGCATCTGTAGTTTT
AACTTTTGTGGACTTCCTGAAGCAATCCCAAGTTACTAGATCTCTCTCTGGTTAAGACAGAGATTTTTTGTTTC
TTAAATTCAGGGCTTACAATCTCTTGGGTCGGTTAACATCCTTGATCAGGTGGTGGGTAATGGAAGAATAAGTC
ATGACTGGCTGTGTCATTCTTGAGCTGTTAAGACAGTGCAAAAGATCTGCTATATTGCTCAATTAAGATACTTT
GAACTGAAGCCCTGTGGTGATTATTGCTGGGCTATCCCCACTCTTCAGATACTACTTCCCATTTCTTCAGGCTA
GGTACTTTGAGTGGGTTTTAACTTGTCCCATAATAAGACTATAGAAGATCTGGTATAATACCAGAACAAAATTG
AATGGTAAAAAGGAAAACTTAATGATTCGGACTATGTTTTTGGGAATGCTGTTGCGGTAGGCTGGAATCAGAT
GTAGAGGAATGGAAAGAGATAAAAGGGAGGAGGGAGTTAAAAATGATTGATTTTTTATATTATTTCAAATGGAA
GAGATGAAGTGGTTCAGTTTTTAGATATTGTTTTTGGTTGCAGAGACTCTTCAACAAAAGTACTACTAGATTGG
GTAAATTTGTTTCTTGCTCTAGGCTAGAGTTTGAGATGAAGGTGTTGGTAGGCCTGGTTTCTTCTGAGGGGTCT
CTCTTGGCTTGTGAATGATTGTCTTCTATTAAGCAACAGTCTTCTCTCTTCATTGTTGGTTGTCACAATAATAA
ATCTCTTCTTTTTTGGTCATGATTGGATTGGGGCCTCTCTAAGGACCTCATTTTAGTACACTCTTTACCAAAGA
TCTGTCTCCAAATGGTTGTTGACATGCAGGTTAAGGGCTTCAACAGGGGGGCAGTGCAATTCAGCCATGATGAA
CATGGTTGCTTGAAGAACTTTGGAGGCCAAATACATAGCCTTCATTTATAATGTCTTTGCTTGATTTGCCCAGG
CTGCTGGTGCAATGGCGCGATCAACCTCCGCCTCCTGGTTCAAGTGGCAGGTTACAGGCATGCCACCACACCAG
CTGATGACTTTTTGGTGAGATAGGTTTCTCCTTGTTGGTCAGGCTGAGTCTTGGACTCTGACCTCAGTCAGCCT
CCCAAGGGTGCTGGGATTACAGGTGTGGCCACTGTGCCCTGGCACAATACTTTTTTGCTTGTGATGCAAAGATG
GAATCAATAAAATTGTAATTTACAAATTTTCCCTGTTTGGTGCAGTTCTTAACACTTTTTGATAGATATTGGAG
ATTCATGTCTTTTTGATTTTATTGTTTACATGTCTACTACGTATCTAGGACCTTATTGCCCAACAATGTTTCTG
CATGTTCATTTCTTCATTTCAAGTCATGAAAGTGCCAAGGGCATTCTATTGAATGGAACAGTTTGAAAGAAACA
TTGATCTTAGCTTCAGTAGTGGTTTTCAAGACCCTTTGCATTTCAATTGAGGACCCCAAGGACTTTTGTTTATG
TGGGTTTTATGTAGATATTTACCATAGCCAAAATGAATGATTATTTGAATATTTAAAAGTAACAGCAAACTTTC
AATGTAATAATAAAATAGCGTTATCTGGTTATTATAAGACAATTGGTCTCCTTTTACGGTCTTTTGTTGAGTGT
TTTTGGTTATCTCACAGATTGCTTAGCTTGAAGGATGGAATATTTTTCAGATGTTTGTATGTTCTTTGATGCTT
TGACAAGTAGTAGTTTGATATCTGAAATGATCACCTTAAAGTCGATTAACTGAAATCCGATTTTGTCACAGTGT
AGCATAGGAGTCAGCCATGGACCAGTTGGACCCACTAAGGATATCGTTTTCAACAAATGGCTTTTCGTGGTTAG
CTATGCCGCCCAAAATTAACTCAAGGCTGATGAACCTAACTTCTGGGAGATCTTGGGTTAGACCAAGGTCTCTT
ATGACTCCAAGCATGATCTAAAGTGGATTAATCAGATGGCAAATTCAATTTCTTTCAAATTCAGCCCTTAACAG
ATGAGGATACAGAAGATGTATCTTGGGTATATAAAATTCTCAGAACAATAACTCAGTTTTAAAAAATGGTCCAA
TTTATAGACACTTTAAAAATGCCAACACATTGGCTTCTTAAGCATACCAAATATGGGCTCATAATGGGAACACC
ACTACCCTGCTGGATGGTGTCAAAAAGACAGGTAGTGTTGGTGAACATTGGAACTTCATTTTGTTAGAATGTAA
GATAGGTGTGACTAGTTTTGGTAATTCTCAAAATAACTGACCTTATGCAACCAGATTGCAGTATTATAGCAATA

AAACAACCAGTCAACATTGGTAGATAAATAGCGATCTTCAGGTAAATGCTGATGCCTTGAATGCATCATAAACA
TTGAATAAGCCAGTCACAAAAGAGCAAACAATTGGTTGTCTGGAATGGTAAATGTCCTTTTGGAATTGATGATA
ATGTTCTAAGATTATGATGGTTGCTATACTTAAAACAATTTGAAGTCTTTGGTGAACTTTTACTGACATATATC
TGGATAAATTGTTAATTAAAACTACTGCAACAGGATGTCATTACAATGATAAAAATGAATGACTGACCAGAGGT
GATGTTTATGGGAGGTGAGCAGTTGATTAAAGTGGTACTTCTTCTGGGGAGCAAGCAACCTTCAGGTACAAGGT
GGAGCCAAGGTTGATGGGCAGTTATGCAGAGGAGTTGGGATATCTGTGGGTGTGTTGAATTAAAACAAAACTTC
TTGTGGTGTGGGGGCTACACCTTTCTTGATGTCTCCAGGTTTGAAAGACAGGGAAGGGATGCATGGATTAACTG
GCTCATTGCTGGTCATTTAATGCTTGAGTGTTGCCCAGCTGTTGAATCTCCCATGACTTGCGTGTCTCACCTAT
ATATTGGCTGCAACTGCTGGTTTAAACAAATGAAAATGGTTTGGATGGAGTTCGCTCTTTAGCCCAGGCTGGAG
TGCAATAGATCTTGGCTGGCTTTTTGAGTTGGCGATTCTCTGCCTCCTCCTGAGGCTGGGATTACAAGCACATT
GACTATTTTTTAGTAGAGACGGGGTTTCCATGTTGACCCAGCTTTGGTCTTGAACTTTCTGACCTCAGTTGATC
TACCCTCAGCCTCCCAGTGCACAGGTGTGAGCCTGCACCCAACCTCCTGCTTCTTCTGATCTCCCAGAGTTGAC
AGGCCTTGGCCTGGTTGAGGCCAGAAAGATTACCAGACTTCAGAACAACAGTACAGGTGAATTTTTTAGTTTGA
CAATGGCTCTAAACTTTGTTGGAATTATTGGTATTATTATTTTTGGGGAGCAAGTTTTAGCTTTCTGATTGCCA
GGCTGGAGTACAATAGCACGATCTTGGCTCCACTCCCAGGTTCCAGCTGCCTCAGCCTCCCCGGTGGCTGGGAT
TGCAGGCACTTCATTTAGTGGTTTCTCCATGACCTTGAACTTTGCCTCCTGGATTGCAGATGAGCCCTGGTGGA
TTTTTTTAGGCTGTGTTTAAGACTGTTCCCAAGTGCTCACTTAAAATTTGACAGTTAAACATTTTTTTCTTGAT
TGTAGCAATTAAACTTTTTGGGGCAGAGAAAGGAAAGAAAAAATTACCCATGTTCTCGTCTTGACCAGATACAG
TGGCTTTTTGCATTTCTTCTTTATGGTGTTATGAGGTGTGGTCATTTTAAAATGTTGTATCTTTCCATATTAGC
AAATAGTTATATCTTTTTTTTTGTTTTTGGTCTGTTGCTTGAGCTTGAATACGGTCACAATTTGGTTCTCCTGG
GTTGACCAGTTAGGTTATAGGCGTGTGCTTTAGTTTTTTGGTAGAGATGGGTTTTGGCTGTTGGACTCAAATTG
ATCTGTCTTGCCACAGGCATGAGCCTGGCAGCCTTGTTTATTTTTAAGGGACTGCCATTCCATATAGTGGTGCT
GCTTATAGGTTGTCTTGAACATTTTTGGGTAATGATTTTATTATGTTTCAAAATTGTCTACTATATTAACTAAT
TGCCAAAAATTTTTGACCAATGATTACATATTATTGAATGAACAAGGTCATTGCCCAGTTGCATGGCATAGATT
GCAGTTCTTGAGACTGTCTGCCAGTTATGATTGAGGGACAAATGTAAATGGCAAAAAAAAGATTGGTGATAATA
GTCAACTTTTCTTGGTGATCACAACCCAGATGTACCTGGTCACAAAACTAGGCTTGGTGTGCTTGGCATAGGCA
GTTGTTCAGTTGCCTTGACCTTGCGTTTAGAGAACAGTTTGCTGTTCACTTTCTTATAACCTCCAGTGAACATG
CTGGTTGATCTGACCTCAGTCTTTTGGCCTCAATTTCAAATCATGTTGACATGTCATGATGCTGATATGATATG
ATATAGATACTACACTTCATGTGATTTCAAACCCAACCCCAGTCTTCATGAATCCTTTTGTTCCAGTAAACAGA
CATTCTGTTAACTTAAGGTGGTCAACCAACTAAATATGATCCTAGAACAGGACGTTAGGCCAAGACTAAAATCA
AACCGTTTGAGTTAGCAATGTATTAATATTAGTTCTGAGTGTAACAAAAGTATATAAATCTAATAAGATGTTAA
TATGCGAGGGATATTGTGCTGGGTATGAACCTCTTTCTTCATATTTTTCTTCAGTCTAGTTTCTAAATCTCAAG
TCTTGTTGGTAATAAATAATTCTTTGTATGTGCGTGTCACAGTTGCTTGAAAGGAGCTTTCAAGAATGAATAAT
GAACATGTACCCCCAAAGTTTGTTTTTTAGGCACTTGGGATGATTATTGAACTGGTTTTCTTTCTTTCATCTCT
GGGCAGTCTCTGTGAAAAAGTGGGGACTTGGCTTTTTTGTTAAAAACACTCTTTGGTGGAATCTTTAAAAGGTG
TCATTGTAAAAGACATTGGTATTCAAATACAAAAATTCTGGATTAAATAAATTTCAAATGGAAGGCTTTTTGCC
ATATTGTTTCTAGTGGGTTTTAACAAGCAAGGTGGTTCAGCACAGGCAAGATTCTTAATTTTACTGCATCTGGA
GCCTCAATGAACCTACTTGATTGGTTTGATTTTAATGCTAGACTTTGCTCTTGGGTGGTGGATTGGAACAGGTA
TACAAAGCAGGCTATTCTTTTTGACTTTAACATGTTTGTGATAATATATTGGCTTTAAGGTTAGGATCGGCTAA
GTTATGCCGTGATCACAAACTACCCTCAGGCAGGTTAATCTTCTTGGGCACGATGGCTCACCTAATACGGTTGA
ATTGACACCCAGTTAGTGGTGATAAGAGGGCCTGCCTCTACACAGGTGATTGAGTGTTTTGTTTGTTTGTTTCT
TTTTTAGAGATGATCTTGCTCAATTGTTTGCCAGGCAAGATGCAGTAGGCGTCATCTGGTTTAGCTTGGGGTTC
AGCGATTTTCCCTCTTGGCACAGGTACTTCACAGGTGTAACGCCACTGCGTTCTCTGATGTAATTTTTTAGTAG
ATGAGTGTTTCATGTTGGCAGGCTCAAACTCTGATCTCAAACAGTCTGCCCATAGCCTCCCAAAGTGTTGACCC
AGGAGTTTGAGGCTGCAGTGAACTATGGTTGTGCCATTACAAAGTAAAACAAAAAATTACGCTTTGATGTGGCT
TGATGACTCCATCCATTGGGCAGGAAAATGCAGTGTGTTCATTAGCTTTCAGACATACCAGAAAGGGGATACTT
GTCATTTATTGCCTCATATTTCATTGACCAAAATGGGATGTCCTATTTCCAGTGCAGTTTCTTGGTGAAATGTT
GATGACCAATGCTGATGTTCTTCTTACGTTTCTACCAGTTGGTTGATATTGCTCGTGGGTGTACACTTCAATCT
ACAATTTCATTGGAACATTTTTAGAGGAACAAGTATGTTTTTCTGTCATCCCTTCTTTCTTATTTCTTTCTTCC
CACCAGATTTCTGTTTTTCAAATTAAAGTATAATTTAATACCGATAAAATTTACCTTGTTTATTATCTGGCAAA
ATATGGTCATGGTCGAGTTCCACCTTTTTACCTTTTATGGGTCAACTTTGTCCCAGCCCAGTCATTTCTGTCCT
ATCGTTCTTTTCCCATAAATAAGTTTCATACAGGTATGGTCTTTTGGTCTGGTTTCTTTAACATACAATGTTTG
ATTGTCACTAGTTCGACTCTTTTTTGTTATTTTTGACTAGCAAGGGATGTACCGGTTGTCTATTACCCCAGTTG
TAGATTCTTGAGTTGATTGTTTCTAGTGGATGAAACCAGATTATACTTTCCATGGGTTTTTGTGAACGCCGTTT

GTTGTTTGTCAGGTTGAAATATGTTAATAGTTCTGGTTGTTTTTTCGTTTTTTTGCCTTTCTTAGGTATGGGTT
TTTTTTTTTTTTTTTTTTGGGACAGGATGCTTATGACCTTTGGTGGTACTTCTGGCTCCTGTTCAAGCAATCTT
ACCTTGCCTCCTGGCTGGGACTACAAGCAGGCATACCCTGCTAATTTTTAAATTTTATTGTAGATATGATTCCC
TATGTTCCCAGGCTGGTCTTGAACTCCTGGGTCCTCTTGCACAAGCCTCAGGTGCTGCTTACAAGTGCTGTTTT
TTTATTACAGTTGGTAAGGTATATTTAATATGCTTGCACCTTATTTTTAGCCATCACCATCTTCTTGTGATTCC
ATTTCTTGAATGACTTGAACATTTAAAATTTTTCCTCTTTTGATTGGTGTTGTTTCAGATCTTTACTGTTTTTA
AACCATTTCTTCTTTTTTAACACCAGATTTTTTTTTTGGTTTTCTTGCTGTTGAATGCCAGTTAACAGACAAAA
GGCTTGAACAGATAATTCACAAAAACTTGGTAACATGAAATATTCAGGTTGTGTGTCAATTAGCTGCTACCAGT
TATTTTTTATCTTTATTTCGGTAATGCTTGTCTTTCGATGGAGCAGTCTACTATGCAGTGGGAGGAATATAGAT
TGGAGGAACAAGATAAATAGAATATTGACATTATGAAAAAAAATACATTCCGATAACCTGGTTGGCAGTGAAGA
AGCATGATAAAGGTCAAAGGAAATTTCTTGGTGGTTTTGATCTCATTGGTTGGAAGTAATTACTTGGTTCTCAA
TAACAAGCTATTCTTAGTTCCAATGATTTTCTGAGGTGTGAGTTCAGGTACCCTGGATCTCTTGGTCTTGTGGC
CCGCTTGGATGATTATATTATAGCCTCAGTTCCAAACTGAGGATTGTTATTGACAGAATACATTCAGGTCTTGA
CTGTGGTGGGTCTTGATGATCTTTCATGCTGATAAGACATAGGACCTGAAAGAAAGTTTATAATGGTGGAGGAC
GATGATCCCAGGATGGCAAAAGAGCAAAGTAGGCTTGATGACAGAGGCTCTTCCAGGCCATCAGATCTCATGAG
ACAGCATTGCAAGACAGTTCAATTACCTGGGTCCTCCCACAACAATTCAAGATGGTTGGTGGGGACACAGCCGT
GATGATTGACAAATAAAGGGTAGTTCTTGGCTGATGTCAAAGTAACAATTGAATGTTATTAACTTTTTTATGCT
TTTTCTTTGAGGCAATTGGCTTGGTCTTTTAAATGCCTTCATTGAAGTTTCTGGTGCAGTATACCAAATGAGCC
ATACCAGTTGCTGCTGGCTGGCATCATAATCCCAGCTATTCAGGAGTCTGGACAGGATGATTCCAGGAGGTTTG
AGACTAGCCCGGGCAATAGTGGACTCATCTCTAAGTTGGCTGGTGGTGTCCTCTGAATGTTCCAAAGTAATATT
GCCAATGAGTAGGCCAAAAAGTTATAGAGACATGGAGGTCAAATGCCAATTAATTGCTTGATTTTGAGAATATG
GTTTTGATGTTTTCTGCTCATAATAAGACTTGAAATTGATTTTCATTTTCCTTATGTTGTTTTTTCTCTAAGAC
CCAATTGAGGAAAACCCTTATCTTAAGATCTTTATTTTTTGTCATGTCTTAATGACTGTTGAGTGGATATAATG
CTTTATGAAATTAACCAGTCATTTCAAGTTTATAAAATGTTTTTGATTAACAATGATCATTAATCCATTCATTA
AATTTTTTTTTATGCTTCATTACAATTGACTCTCAGAAAATAGATGCTGAGATTTAAGGAGTTATTAATATAGC
AGATAATATGTCAGATTCCACATTTTTTCCCAGCAGGACTAACTGTCATTGTTTTCATATAAAGCCAAACCTGT
TTCGTTAATGCACTTGTTCTTCCTCTTGACGAACTCATGAGGTATTTGATTTGTTTTTAAATATATGCGTTTTC
GTGGCTTCTTCAAACTCCCAAGGTGGTGGTTGTTTTATGTTGACTCTTTTTATTTTGATGGCTTGGCTGGGTCT
TTTGTTCTGTTAGCCTCTAAACCCACAGCAAGACAATGAGGTCTTGAACTTGGGCATGGCTCATTATAGCAATC
CCTAGTGCTTTGGGAATGAGATGGATTACTTACAGGCCAGGAGTTAGAGGAGTTATTGAGCTATAATTGGTCTT
CACACTCCAGCCTTTCATGTACTGGATATTGATTGTCTTTGCATTCAGGTGAAACTTGGTCCAGTTCTCTTCTC
TAGGCCAAAGCAGAGCCCTGGGGCTTGTTCCAGAGTTGTTGGTATCTTGTTTGTTGGTTTGTCATTCCAGAGGA
CAGGGTCTTCAGTGCCCTAATTCGTAATGATGACAATAAGTGGAGTCAGCAACCTGTTCACCAGCCTTGGAGTC
AGTGCTTTGTAATTGAGTGTTATTGCTTTTGCCGGGTCACGACCACAGCTTTCTCAGGCAGGTGGTGTTTTTGT
CATGAACCAAAATGACCAACGATGATGTCGCAGCAATGTAAGGACTGTGCTGCCGCCAGTCAATTATGTGCCGA
CCCGTCTGTTCTGGAGACACCTTTGTCATGCACAGCAGCTGCTTTATACCTGCTTCCTGCTTTTTGACCCATCA
CAGATATTGGGCTGAGGCCCCAGGCTTATGCAGAGATGCATCCACTCACAATGGCCCTTTTTATGCCAGTTTCA
AAAGATTGTCCACAGTGACTGACTGGCTGAATGAGATTTCCTTTCCTATATAGATTGTAGAGATCCGCCTGTAA
CTTCGGCTCTGATTCTGGTGCCTAGATGTCACATTTCTTGGGTGGCAGTTGTGACCCACGACCAAGAATCTCTG
ACTTGGAACACCCCAGACAGACTGCAATACGTAAT

m) 2-IV-9_4 (-)

ACATTATACTTCGTTCAGTTACGTATTGTAATGCAAAATCAGAGGTTCTAGGTGAAATTCTAATATTAAGATTC
TTGGTCACGGAAATTACTCAGTAGGCATTGGGAGGCCATGGTGGAGGGTGGGGAGAAAATGTGATTGATCTAAG
GTTACAAATCACTGAAGCCACAGAGTGCGGGTCAGTCCTACAATAGCAAAAGTTATCTGTAGGGCTGAACCCTC
ATCCAGCCAAATTCACAACAATGCTGTGGATTAACTTTTTTGAAACTGGCATGATACTGCTGCCAGGATCTGAG
GTGGGTGCAAGTGGTGGATGTACTGGAGGCATGAGGGTTTTTGGGCTCTAAGCCCATTCAGATGGCTGTGCAAG
TGGGAAGTGAGGGTATGCTTGGCCACTTGGCATGACAAGGGTGTCCGGAACATCATTGACGGTCGGCCCAGCCC
TGGGGAACCAGTCCTGCTGGCACGGCTGAGGCAAGGACAACAGGTGGAGGTGACATCCCACATTAAGGAATTTT
AACAATTGGTCACTTGGTGGGCTCATTCCATGATACAAAACACCTGAACAACCTGGCCCTGCGTGCTGTGGTCA
GGACTGAAGCAGTACAGACACACTCTTGGGAGCTTGCTGACCAGAGTGGATGACTAGCGAGATGGTGGAACAGG
ACAGATTGGGTGGGGAGACATGGATCTCCTTAGAAGGATGTGGACACACAGATTAGGACAGACCCTGGATGATG
ACCAACAAACTCTTGAGATACAAACCCAACAACTCTGAAGCCCAGGAACTGGGATCCCAGGTTCTCACTCAGAG
GGAATGCCAAAGAACACAACTATCTAGTAACACTTAGGTTTTGTTTGCTGTTGTTGTTGTTTGGGAACAGAGAT

CTTGCGCTGTGTCACTTGGGCTGGAGTGCGAGGAGTTGACACCACCATGGCTCTATGACACTCAACTCTGGCCT
CAGGCAATCACCTCCCATCTCAGCTCCCAAAACTGGGATTACAGGCATGAGCCTAAGACTTCTGTTTCACTTAT
TGTCTTGCCAGTTTGCCAGTGGTATTTCACAGGAGCTTCATGAGGATAATGAGATGAATGCAGAGATGAAAAAG
ACCAGCTATAAAATAGATTAAAGATTGTGGAATATGAAAACAAATCCTTTCATCAATTTTTGAGGTTTGGAATG
GTTATTTATGTTAACACATAATGAGTGTTCATTTTTAAAATAAAATTAATAAAAATTGATTTGGAGTTAGATCA
GGAAGAACAAGAAGTGCAATTAATCCATTTGTTGCTTTACAGAGGTGGTTTTGATTCATAGCAAAACAATGATG
AGTTAGTCTCAAGTGGGGAAAAAAAAAATAATCCTAAAGGGAATTCTGACATATTATTGTTCCAATGATGATAAT
AACTCTTTAAATCTCTAGCATCTGTTTTCTGGAGTCAGATTATTATATGGAGAGTAAATAAAAATTCTAATGAA
TGGTTAATGATCACTATTTGCTACAATAACTTTTTTAAGAAATCAAAACATATTTTATAAAACTACTGAAATGA
CCACATTTCATAAAGCATTATATCCACTAACAATAGTACACACGAACATGATGATAAAGATCTTTAAGATGCAG
GTTTCTCAATTGGGTCTTAGGAAAAACAAATATAGAAAATGAAAATTGGTATAAATTAATCAAAAATCAAAATG
ATGTTTTCATGTTCTCAAAATCAATTTAAGTAATTAATTGGCATTTGATCTTCATGTCTCTATGTCTCTCTTTT
TTTTTGGCCTCTCATTGGCGGCAATAAATATTACTATTTTGGAAATATCTATGAGGTGGGCATTACAGACACTA
AGGATGGGGTCTCACTATGTTTAAACTCTGGCCTTGACAATCTCGGACTATGAATAAGCTGGGATTATAGATGT
AATCCAACCGGCAAACGCATGTCTTTAGTTTCTGTTTGGTTATGATGTGCTGGAGGCTTTGGTGGAGGACGTTT
TAAGGGATAAAGAGTTGGATCGTGGGGACAGGTCTTTTTATGATGCTATAGGTGATGGTGGATAGGTCTCATGG
GGATCAGGGTTCTCAGTTCTTTGCTCAGATGTATTCTGTCACTTACTCCCCACTCACAATACCAATCTCTATGG
TTTGAGGTTGAGGGGTGAGGTAATATAACCCCTCTAAGGCCACACAAGACTAAGAGGGAGGCTGGTATACAAAG
GATCTGGGGTGTCACTGAACTGTCACACTTTCAGAAAACTGGCTGGAATCTGGAATAGCTAATTCTATTTCGGT
TGCGAACAAGTGATTATCAACCAGATAAATGAGGTTAAAACTAGCTCCCAGATTCAAATTATATCGCTTTTGAC
CTTATCATGCTTCTTAACTACATGGCTTTCAGGTTATAAAGTGAATGTTTATTTTTCATAATGTCAATGTTATT
CTGTTGTCTTAACTCCTCCAACTGTCATATTCTGATGAGCTGCTCATCGGGTAAAACATAGGTACTGGAATATG
AAGATGAAAAATAACCGGGTGGTGGCAGCTAATTAAAGTGGTTTTATTGCTGCCAGCCTGAACATTTCACAGTT
TACTGGGGTTCTCTTTTCTTCAATGAACTATCTGTTTCAGAGTTCTTTGTCCGGTTACTAATCAAGGTCTCAAC
AGTAAGAAAACTAAAAAATCAGCTTTTGTGCTGGGTTAGTTAAAAAATGGTTTGAAAAAAATCGGCAAAGATCT
GGTTTTCCACTCTGAAAGAAGGACAAATTTAAATGGAGATCATTCAAAAGTACTGGAGATTTAAGAGAGATGGT
GGTGGTTGCACAACAAGGTGTGTTAGTACTAATATGCCAAAATAGTTAAAATAAGAATTAGGATTTTTATTGCA
ATATGCTTTATACAATCAAATTTTTATTAACATGAATGTATTTTGAAGCTCACATATGAAATCCAACACTTTGG
GAGGCTCAGACAGGGATCACATGAGGCAGGGGGTTCAGGATAGGGGACCCATATCTGAAATGGAGGTGTGGATG
TAGCGTAGTCCAAGGGTGTGGGATGGGGATCGGTGGGATGGACAAATGGGGATGTGGATTGATTAAATGGTCAG
TGCAATGACAAGTGTCCTTCTTGAGGTGGGGTGTCCTGAGAAGCCAATGAACACAATTCATCACACTGCGTTTT
CCTGCTTGCCTCAGTAGGTGGGCATGCAGTGAGGGATGGGCTTCAAACTGAATGCAGTGACTGCAGTTAATAAA
AATAAAGTTCTGGTGGGCAGACTGTGAGATCAGGAGTTTGAGACCAGCCAACAATGGTGAAACACCATCTCTAC
TAAAAATTACGAGGCATTGGTGGTACCAATGGGAAAATCGCTTGAACCTGGGAGGTGGAGGTTGCAGACAGCCC
TGGGCAACCAGCCAGACAGACTCTATCTCAAGAAACAAAACAAAACAAAAAACACTCCTGAGCTTAAGTGATCC
TCCTGCCTCCCAGCCTCCCAGCTGGGACTACAATTTAGCTAAATTTACTCTTTATTTTTGTGAGGCAGGATCTT
GATCTGGTCTCAAACTCTGGGCTCAAACAGTCCTCTGGGTGATGGCCTCCCCAGAGCTACGCCAGTATTATAGG
TGGCCGAAGTGATTAACACGATGCTTTTAGCTCAGGAGTTTAAGGTGGTTGTGACTAAACGCTGATCTTAGCAG
CTTAGCAAAATACGACTATTACAAAAGATAGCCTGTTTTGTATGCCTGGTTCAATCACCAACAACAAGGCCAAG
ACTACAAAGTGGGGAGTCTACAAGGGCTCGGCAAGTCGCTACTAAAAGGTGGAGTTCTCTGGCAGATTAGGCTC
CCCATCAGATTAGGTAATTATGGATCTTGCTCAGGGCCAAAGTAAAATCAATGCATCTTGCTTATGGACATAAA
CAATATGGCAAAACTCTTCCTGGTTTTCAAATTTCTATTTAATCATAGCCAATTTGTTTGCTGTATTTGAATAC
CTAGTAATGGATACCTTTTAAGATTCCTTGGCTCAATGGTGGTTTTTGATAAGTCCTTAGTAATTTCAAATTCT
TGGGAGATGGGAAGAAAGAAACCACCACAGGAGTCAGCCCAAAGTTTTTTCTAGCCAACGGAGTGCAGTTCAAT
AATTTATCCTAAGTTACCTGAGTATGGGGAAAAAAACTCTAAGTTTTTGGGGTACATGTTATAACTCTTAGCTG
ATATCCTATCTTGAAGTTCTTTGATGCTTAAGACTTCAAAATCTCTGGTGTTAAATCGATGATAATTTAATATA
CAAATTATTTTATTACTAACAGACTTTAGAGTCTTAGACTTGCAGAAAATGAAGAGGTGCAAGGTTCCCAACAA
TACCCAACACACAACATCTTATTAGGTTGTTACACTTTTTGTTACAACCAATGTTGATGCATTGTCATTAACTC
AAGTCCATGGTTTATTCTGATTTCCTTGGTCTTTACCTAACGATCCTTTTCTGGGATCTTACATATGCTCATGG
GCTCCTCTGGGCTTGGTCTCTCAGATTTTCCTTGTTTCTGACGACCTTCAACAGATTTTAAGGTACTGATCCGG
AGTTACACTGTGGAATGTCTGTTTATGGAATTGCATGATTCATGAATGAACTGGTTTGGGAAGGAAAAATCATG
GTGGCATCTTATGTCATATCATATCATATCAAGGGATATGCCATCAACATGATTTGTAACTTTGTTGGAGGCAG
ACTGAGGGTTGCGTGATCAGCTCAGTATACCACTGGAGGCTATAAGGTGAACAACAAACTGTTCTCATAAACAT
GGAAGAATGTTGGCAAACTGACAAACTATATACCAGAAGGAATGCTGAAGGCAGTCAGCTAGCTCAGTTCTTGT

GATTGGGTGCATCTGAAGCCTGTTAATATGGAGCGATGTGATCAATTAAGCAATGACAGTCATTATCACCTCCC
CAATAAGTTCTAAGGGCTGTGGAAGCTAGGCTGCTGCCTTTGCTCTAGAGCAGGAGGCCCTCTTTCTTCCTTTT
TCTATTCCTTTAAGAAGTTTCTTTTGTCTTAATTTTCATTTCTACGATTTGTCCCATGACGGTCTCAAGTAGTA
ACAGCAGTAACTGTCATAGTGACAGTCTCAAGTAATAATAAATGACATGACTGTTAATGTTGACCTTGTTCATC
TGGCTGGAGTAAACAAAGGAATGTTTAATAATGTGATTATCAATAAATTTTACAATTGGTAGCTACAGTTATGA
TTGAACAATTTTGAAAATATGTTAAAAATATTATAAAATGACTTTCTATATGGCAAAATGGGAGGCGGCTATAT
AGATTTATTCACTATATGGAATGTGGTCCTTAAAATAACAAATACAAGGCTGGGCGCAGTGGCTCAAATATATG
GATCACTTGAGTCAGAGTTCGAAACCAACCTGGCCAGCATGGCGAAACCCCATCTCTACTAAAATGCAAAACTG
ACCAGGTGTTGTGGCTTTGCGCTGAGTGCAAGAATCATTTCAACCCAGGGCGGAGGTTGCAGTGAACCGAGGTG
GCGTGACTGCATTCAGCCTGAACAGACCAAAAACAAAAGTATATGACTTTATTTGCTAATATGGGGAAAGATAT
AACATTTTTTAAATGACCACACTCATGGCTTTTCCAGCTGGGTGGCTTACCATAGCTAAAGAACCCAGCCACTG
TGGTGGTCAGGCAAGGAACATGGGTAAATTTTTCTTCCTTTCAGATCCCAAGTAGATAGTGTTTAATTGCTGCT
TTACAGGCAATGTTTGCTCAAATTTACAAATATTTCCTTTTGGTGGCGTATACCCATAAACAAACTATGTAGGT
TTGGGGAATTAGGCCACTTGCAAAACACAGTTTAAAATTCAATTCTCGGCCAGGTGCAGTGGCTCACGCCTGTA
ATCCCAGGAGGAGGCTGAGGTGGGCGGATCTGAGGTCGGCAGTTCATGACCAACCTGAAGGCCAACATGGAGAA
ACCCCACCTCTACTAAAAATAAAATTATTAAGGTGTGGTGGCATATGCCTCCCAGCTACTCGGGAGGCTGAGGC
AGGGAGAATCGCTTGAACCGGGAGGCGGAGGTTGCGGTGAGCCAAGATGGTACCATTGTACTCAGCCTGGGCAA
CAAGAGCGAAAACTCTGTCTCCAAAATAATAATAATAATAGTAATTCCAATTCTCTATGTACCCCCAAAGTTTA
TATTTTAGCTCACTGGTGCTATAGTTTACTAAATTAAAATTCACCCTGTACTGTTGCTCCTGGAAGCCTGAAAA
CTGCGCTTTCTGGCCAACTGGGCAGGGAATCAGTAAGGGATCAGGGAAGCAGGGAGGCTGGGTGCAGTGAGGCT
CACACCTGTAATCTCAGCACTTTGGGAGGCTGAGGTGGGTAGATCAACTGAGGTCAGGAGTTCAAGACCAGCAG
CCAACATGAGTGAAACCCGTCTCTAAAATTAGCCGGGCGTGGTGGCATGTAATCCCAGCTACTCAGGAGGCTGA
GGCAGGAGAATCGCTTGAACCTGGGTGGCAGAGGTTGCAGTCAGCCAAGATGCTTTAAATGAAACGGAAGACAA
GCGAAACCATTTTCTGCTTGTTTCTAAAATATCAGCAGTTGCAGCTAATGGCAGGTACTGCAGACTCACTGCTA
ACAGATAAAGTCATGGTGGAGATTAATGGCTGGCCGGGCAACATCGAAGCTTCAGCTGGGCAATGAATGATTCC
AACAATTTCAGCAGTAATGAGAGCTAGTTGTCCATGTGTCCCCTTCTCAACTCAATTCTCACTTTCGGTCCCTG
TTCAGGGATGCTAAACTTTTCTTTTCTTTTTGGCTAGGGGGCAGTTAAACTTTGCCAGTAGAGGTGCTGGAGGG
GATATCTAAGAAGAAAGGGTGTTTTCTAGCTCTGCCACTATCAGTAGTTTGTTTAATGAACACCCAACTATGAT
GAGGATTACACTCTTGGGACTTGCACAGGTACACACAGTGATATCCCAACTCCTCATAACTGCCCATGGCAATG
GCTCCACCTGTACCGGAAGGTTGCTTTTCTGCTCCCAGAAGAAGTGGTGTACCACTTTACACTCCCACCAACAG
TATATGAAAATTCAACTTTCAACTCTACAAACACTTGAGTGCGGTCGGTCATTCTATTACCATTTTAGCCACAC
TAATGGGTAATAATGATATCTCACAGTGATTTTTAATTAACAATTTTATTCGGATATATGTCATAAAGTTCACC
AATTTAAGACTTCAATTGTTTTTATGTTACAGTTACTTGTAACCATCACCATAATCTTTTTAGAACATTATAAG
AAATAAATAAAAACCCTGTAACTATTAGTGGATACTTCTACTCCATCTGGTTCAGACACTAATTTCTCTATATA
TATAGATTTGCCTATTCTGGACATTTCACACAAATAGGATCATAGAAAATATGTTCTTTTGTAGAGCTAATTTC
TATCTAATGTTTATGAGATTTATCCAGAAGTTGTACCAAATAGTGCTTAATTCCTTTGAACTGCTGGGTAATAT
TCTGTTTACAAATAGATCTCATCAGTTGATGGACACTGGGTTGTTTCTATTTGGCTATTATAAATAATACTGCC
AACTCATATACAGGTTTTGTGTGAATATATTTTTGAATTCTCTTGGTAAACATATGAAAATTTCTGGGTCATAT
GGTTACTCAGCTTATCTTTTTGAGGTATGGTCACTATCTTACACATTCCTAATAACAAATGAAGGTTCAATTTT
CTACAATGCTACAACATACCTGTTCTTTCTGATTATATGAAACTAACGAATTGTGAAGTGAATTATTATGGTTT
GACTAATGATGCTTAGCATGTTGCTTAATAGCCATAATACATGTATTTTTTGAAGTGTCTATATACAAATCTTT
TGGACCATTTTAAAAGCAAGTTATTGTTTTTTCTTATTACTGAGTTCTGAGAATTTTTTATATACTCAAGCCTT
ATAAGATACATCTTCTGTATCCTGTCTTCATTCTGTTAAGTGTCTTTCTAATTTTGAAGAAATTCTGAATTTTG
ATGCCATCTGATTTATCCACTATTTTTTATGGATCATGCTTTTTGAATCATACCTGAGATCTTTAATAATTCAA
AGATTCAGTTCATAGTTTTAGGTTTTCATTGAGTCTAATTTGGTATATGGTACAGAAAGCTATGGCTCAAAGTT
CTTTCCTACAGCTATTCAACTATTCAGTGCCATTTGTTGAAACGATATCCTTTATATTTTATGCAATTGCTTTA
CTATGATAAAATGTGGATCTGTTACTGGACTCTGTGCTACTACTGAACAAATGGATTTTTAATTTAACAGTTAA
TTTAATTTTTAAGGTGAGCTGCCATGTTTTTCAGATCCGTTAAGAAACTACTTGTCAAGTTTTGACATAGTATC
AAAGGAACATACAAACATCTGAAAATATTCATCCCTTTTCAAGCTATAAATCTCTGTTATTGATGCCGGATTTT
TTATATACTAATAAAACATAACAAAGTGACTGCAAGGAGATGAGGAATCTTATAATAGTCAGATAGTTATTTTT
GTTAACATATAGGTTGTTATTTGCTTTTTTAAAGTATTCTAAATAGAACAACATTCATTCTTCATTTCTATTAT
GGTAAATATCTTAATATAAAACCCACATAAGGTCCTTTGGATGTGGGGGTCTTGGCTCTTTCAGACTGTTCCAT
TCAAATGAGGAAATGCCTCTTTTTCAAGGACTTGGAACATTATGGCTTTGTTACTTTCAGGTCTGGATGCAGTG
GGCATGCAATGTTGGTAAATGGGAAGTGATCAAGACCCATGGAATCTCAATATTCTATTTCAAAGGGTGTTATA

ACAGAACTGCACCTAAAACAGATGAATTTAAATTAATAGCACACAGTTCCATCTTTTTGGAATGAATTGACCAA
GAGGGAAGGAGGGAGATAGGAGAGTGAGGTGTGACAGTGGCTCACACCTGTAATCCCAGCACTTTGGGAGGCAG
GGGTGGGCCGGATCACTGAGGGTCAGGAGGTTCAAGACCAGCCTGACCAACATGGAGAAGCCCCATCTCTGCTA
AAAATTAGCCAGGTGTGGTGAGGGTGCGTACCTGTAATCCCAGCTTCTGGGACAGGAGAATCCCTTGAATCAGG
AGGTTGCCGAGGCCGGATGGGCCAAATTGCACTGCAGCCCTGGGCAACAGAGCTAAACTCCGTCTCAAAAAAGA
TATTATATAATGAGTTAAGTTATAAAAATAAAGAGGAATGGCAGTCTTCCAAGCCTTCATGGGCTGGTGCACTG
CTTACTCCCCGTAATTCACGTGTTCAGGCCGTATGCCTGATGTGACTGTGTTTGGACAGGATCTTCAAGGGTGA
GTAAGTTAAAATGAGGTCATGAGAGTGAGCCTAATCCAATATGATAAGTCTTTAAAGAGATTGGGACAGAGGAC
AACTACATGAAGACATGAAGACACGAAGACAGTCATCTACAAGCCAAGGAGACCTCCAGGAAACCAGGCCTGCA
ACACCTTCATCTCAGGCCTCCAGGTTGAGAAAACAAGTTCTCTTAACTTTTGATCCTGTGCTGTTACAAAAACA
ATGAACTATATAATATAAATCAGTCATTTTTAACTCACTCACTCCTTATGTTACCTCTTCAGATTATGTTCTAT
TAATTCTATTTCTTCTTTTCCATTCTTTCATTCCTCTGCACCCAGCAATGTAACTCCTGCTGTCTCATCCCAGC
CTGCAACGTTCTAAATATAGTCTGGTGTTAAGTTTTCCAGGTTCTTGCTATTCACTTTTTCAGACTCTTTCTTA
TACTGAAATAAGAGTGATCTTCATGGTCTTTTGGGACAGAGTAAAACCCACTAAAAGTAAGCCACCAGCCTGGA
AGGAGATGGGCATCTGGGAAGAGGGATGACCCAGCAATAATCACTCACAGGACTTCAGTCTAAAAGTGATGAAT
TGAGCAATATAGCAGATCTCTTTTGCACTGTCTTACCAGCTCAGAATGGACACAACCAGTCATGATTCTTATTC
TTCCATTACCTTTTCCACACAGCTACCTGGATCAAAGGATAAGCAACTGACCCAAGATGAGCCAAGTTGTAGCT
TCAGGGAATTTGAATTTAAGACAAACAAAAACTTGTCTTAACCAGGAAGATAGACTCAACCACAGAGTTGGTTT
GAAGTCCTGAGTGCTTAAAATCCAAGTCCTTATGTAAAACAGCATGGTATTTCCAAGTAACCTATGCACATCGC
TATAGGGTCTGAACAAGAGTTAGGGCACCGGCCTCCTGCACAATCAAAATCACATGTAGCCGGGCGCTCACGCC
CATAATCCCAGCACTTTGGGAGGCTGAGGCAGGCAGGATCACAGGTCGGGAGTTCAAAGTAATGGTGAAACCCC
ATCTCTCTGAAAATACAAAAATTAGCCAGGCATGGTGGTGCATGCCTGTAATCTACTCGGGAGGCTGAGGCAGG
AGAATCGCTTGAACAGGAACTGAGATGGGTGCCACTGTGTGCTCTAGCCTGGGCAAAGCAAAACTCTGTCTCAA
AAAAAAACACACAACTTTTGACTCAGCGCTACTAATGAAACTACTAACAGCCTGGTGACCAGAAGCCTTCAACA
TAATACAACATAAACATTAGATTAATACATATTGTACTACTTTTTGAGACAGTCTTGCTCTGTTAGCAGGCTGG
AGTGCAGTGGCGCGATCTTTCGGCTGACCTGCAAGCTCCTCCACGTTCACGCCATTCTCCTGTCCTCAGCCTCC
CAAGGAGCTGGGGTTACAGGCACCATACCACATCCAGCTAATTTTTCTATTTTTAGTAGAGAGACAGGGTTTGC
CATGTTGTCAGGCTGGTCTCGAACTCCTGACCTCAGGTGATCTGCCACCGCCTCAAAGTGCTGGGATTACAGGC
GTGAGCCACCAACCTTTGTGATAAATAGGTATTATGAGTACTCAGGGGATTATTTAAAGTACAGATCTGTGAAC
ATCACAGTTTGAACTATGGGTCACTTATGGCAGTTCTTTCTTCCAGCCAAATATGTTGGAAGAATTTTTGGGAG
GAGATTCACAACAATTTAAGATGAACTGTGTAGAAGGAACAAAAAATAAAATTATGCCATGAATGCATGGCTTG
TGATTATGTAATGCGTACTGTATTATATGCATTCTTACAATATATTGTTATTAATATATACTAATATTCTATAT
TATATGCTTTGTTAATGAAACTGAAAAAATTAAGAAAATCGAAAGGAAATTACATTTTGTACTGTACCATATTT
GACTGATACCTGTCTGTTTACAAGATAAATCATTCTGAAATGACCCAACAACCACAACTACAATTCTCAATCTG
TGGCACATATGGAGAGCAATTACGACTTTTTCTTCATGATAATCATGCCTTTTTCAGCTTCATGAACTTCAGCA
TCATAGTGGCACCTCATATGGATCCCATCATGTTATTCAAAAGTTGGTGCTGCACTAAATGCAATGAAAAATGA
GAATCGAAGATACTTGTAATTTACTGGAGAGATGACTCAACTTTCTTTTCAACAACTTGAGCTCACAACAACAG
CAACGGAAGGTGGCCACAAAAATTATTACAGTAGCACAGTATGCTGCCCAGTTAATTTCATGCAGTTATGATTT
AATACTGTATCTTTAAGTTTTATTTTACATCTCTCAACTGTGAATGGTGCAGTCTCAAGTACATGTTTGATATT
CGTAGTTTTGATAGAATGCAACATTTCATGACAGATTTGTATGTTTATGATGGTGAGTAAAAGACTAGTATCTA
CATATTTTATGCATTATTGACATAACTTTTTAACTGTTCTAGACTACACAGTTCATCTGCAGGTTTTTCAGAAT
TTTAAATCTCAAAATTTTTCAATATGTTGTTGAAGAAAAATCATGAGATGAACCATGTAGTTCCAGAAAACCCA
TGTTGTTCAAGGTCAAAATTGTCTCTTGGGAATAATTATAATAATGGTCATCGTACTGTGTGTTGTTTGAGATA
ATGACAAGAAAAAAAAGGCTGTACATATTTCAATTACAGAGGTAATCATCAGCCTTTTTCCAAGTATTTTCCAA
TGACATTGGATGAATCACAGATCTGAAACCCACAAATACAGAAAGCTGACTGCATATCAAGACAATTTATGTCT
CCGTCAGCTCAAAACCCTCATAGCTCCCTATCTCATTTATAAAGTCAATGAAGTCTAAAAGCTCTCTCCCCATT
GGCTCCATATCTTTCTGACCTCATATTATGCTTGCTCTTCTCAACTCTTTACAGCTACACTGCTCTCCTGGTGA
TTTATTGAACACTCTAGGCATGCTCCCATCTTAGACACTTCACATGGAGGCTATGTTCCTCTGCCTGGAATGCT
CTGCCAGAGTATCTGCATGAAAGCTGCTCCATACTCCTTTGCTCCTTCTCAGTGGGGCCTTTACTGCTATATTT
AAAACTGCGACCTAGTGAGCAGATTGTGCTCACTCAGCCTGGGTGACAGAGCAGAACTCGTCTCAAAAAAAAACA
ACCATGCCCTCATATCTTCTTATTCCCCTCCCAATTCACTGTTCTCCACAGCGTTTATAACCTTCTAGTTATGC
CATATAATTTGTGTTTATTTGTCTTAGCTAATAACTATAATGTAAGCTCTATGAAAGAAAGGATTTTCTCTATA
TTGTTCATGCCTAGCTTAAATGCATAGAATAGTGCCACCAGACTCATAACAGGTCACTCAGTAAACATTTAACG
AATAAGAAATGCATGTACACTTGGTCACAAGCATGCCAAAACAGTGAAAGGTAATGGCATTGAGGCACAGACAT

CTCTGAAACAAAGACATGACAGGTCTAGAAAGAGAGGAACCTTTGTCTGATTCTGACAACTTTCTATCCTTTTA
ACTCATTTCTTCAAATTTAAGTTACCTCAGAATTTTGCCATGGTGATATAGGAACATGATCAACCATAATGCTC
TTTGGCTTCACACTTTTTGAATGAATATGTTCAAGTAAAGCATAAAGAACAGCTGCTTTGGTATTCTGTAATAA
ATTATAAGACTTTGTATTATGTTGTTAACTAATACAAGCAGTAATTTAGCAAAATGGAAAGAAAAAAAAAAAAA
CCTCAGCTTTGTCACTGAGGCAAAATGTGGGCATTCTGTGACAAAATGTTTAATATTATCTTTGAGCTGCACAT
CAACACTTTAACATCTATTCCAATGTTGTGCTGCTTTTCGCTAAAAAAAAAAAGTATGTCCTTGACTGAAGGAT
GAACTTTCTCTGAAACCTGAAATCTCAATTACTGCATTGCAGGATCGGACTCTAACCCATAAAATGAGGAGGAA
CAGAAATGTCTAAGGTCTCTATAGCTCTGAGTTTAAAATAGGATGGATAACAGTTGACATAACACATCTGCACT
CATACAAATAACATATACACATAGTAAGAATGATTTATCATAATAGTATGAGGAATGGATTATAAACCTGTTGA
TTTAAAAAATTAATTTAATACTGAAACTTTGAGATCTTCAAAATCTAACCAAACAGGTATTATGGAAACAGCAT
GTAAGTATTACAAATGCATAACATTCTGAGCATCAGACTATCATATATGGTACCATCAGAGGCAAACAATGTGC
TATTTTAAAAAGGAAGGAAAGACTTACTATATGCACGGTTGATGTGTTGTAACATTCTTAAATCTCTTTGCTCA
AGAATATTAAATATATTTTTAAGGACAGAGAACCGGCAAACGGTAGCTCATAAAATAATCAACATTTGGGAGGT
TGAGGCGGACAGATCTGCCTGAGGTCAGGAGTTTGAGACAGTCTGGCCAGCATGGCGAAACCCATCTCTAAAAC
ACAAATTAGCAGGTGGCGCTCCTGTAATCAGCTACTCAGGGCTGAGACAGGAAATCGCTTGAACACAGGTGGGA
GATTTGCAGTGGCAGATCATGCCACTGCACTTCGCCTGGAGCAACAGGGCAAGACTCCATCTCGGAGAAAATAA
AATAAGAGTGAAATGAAATGAAATGAAATGAAATGAGAGATGAAATGAAATGAAATGAAATGAAATGAAATGAA
AATGAAATGAAAATGAAGGAAAATAGGAAATAGAAATGAGAAATGAAATGAAATGAAATAAGAAATGAAATAAA
AATGAAATAAGAAATGAAATAGAGGAAAATGAAATGAGATGAAATGAAATAGAAATAGAAATGAAATAAGAAAT
GAAATAATGAAAATAAATGAAATGAAATGAAATGAAATGAAATAGAAATGAAAATAGAAATAAGGAAATAGAAA
TAGAAATGAAATAAGGAAAATAAGAAATAGAGAAATAGAAATAGAGAAATGAAATGAAATGAAATGAGAAATAA
GAAATAATGAAATAGAAATAAAAGAAATGAAATGAAATGAGAAATGAAATAGAAAATAGAAATAAGAAATAAAA
TAAGAAAGAAATAGAAATGAAATAAAAATAGGAAATGAAATAAGAAATGAAATAGAAATGAAATAAATGAAAAT
GAAATAAGAGGAAATAGAAATGAAATAGAAATAGAAATAGAAATAGAGAAATAAAGAAATAAGAAATAAGAAAT
AAGAAATAGAAATGAAAATAAAATAAGGAAATAGAAATAAGGAAATAAGAAATGAAAATAAGAAATGAAATGAA
AATGAGAAATGAAATGAAATGAAATATGAAAATGAAAATAAATGAAAATAGAAATAAATGAAATGAAATGAAAT
AAGAAATGAAATAGAAATGAAATAGAGAGAAATGAAATAAGAAATAAAAATGAAATAAATGAAATGAAAAAATG
AAGAAATGAAATAAATGAAATAAATAGAAATAGAAATGAAATGAAATAAGAAATAAATGAAATGAGAAATAAGA
AATGAAATGAGAAATAGAAATAGAAATAAGAAATGAAATGAATAGAAATGAAATGAAATGAAAATGAAAATAAA
ATGAAATGAAATAAGAAATAAGAAATGAAATGAAATAGAAATAAGAAATAAATAAATGAAAATAAGAAATGAAA
TGAAATGAAATGAAATAGAAATGAAATAGAAATAGAAATAGAAAATGAAAATGAAAATGAAATGAAATGAAATA
AATAAATGAAAATAAAATAGAGAAATGAAATGAAATAAGAAATGAAATGAAATGAAATAGAAATGAGAAATGAA
ATGAAATAGAAATAGAAATAGAAAATGAGGAAATAATGAAATGAGAAAATAAAACAAAATAAATAAATAAATAG
AAATAAGAAATAAAATGAAAATGAAATGAAATAAGAAATGAAAATAAAAATAAATAGAAATGAAAATAGAAATA
GAAAATAAATAGAAATAAGAAATGAAATAGGAAAATGAAATAGAAATAAGAAATGAAATGAAATGAAATGAAAT
AAGAAATAAGAAATAAATGAAATGAAATAGAAATGAAATGAAATAAGAAATGAAATGAAATAAAATAAAATAAA
TAAATGAAAATGAATAGAAATAGAAATAAAATAAAATGAAATGAGAAATGAAAATAAATAAATGAAATGAAATA
GAAATGAAATGAGGAAATGAAATGAAATGAAATGAAATAGAAATAAATAGAAATGAAATAAGAAATAGAAATGA
AAATGAAATGAGAAATAAATGAAATAAGGAAATGAGGAAATAAATAAATGAAATGAAAATATGAAATAGAAATG
AAATGAAAATGAAATGAAATGAAAATGAAAATAGAAATGAAAATGAAATGAAATGAAATGAAAATAAAATAGAG
AAATAAAATAAAACCAGAAATGAAATGAAATGAGAGAAAATAGAAAATAGAAATGAAATAAGAAATGAAATAAG
AAATGAAATGAAATGAGAAATAAGAAATAGAAATAAGAAAATGAAAATGAGAAATGAAATAGAAATGAAATGAA
ATAGAAATGAAAATGAAATAGAAATGAAATAAGAAATGAAATAGAAATAAGAAATAAATGAAATAGAGAAATGA
AATGAAATAGAAATAAGAAAATGAAATGAAATGAAATGAAATAAATAAGAAATGAAATAAGAAATGAAATGAAA
TAAGAAATGAGGAAATAGAAATGAGAGAAATAGAGAAATGAAATAAGGAAATGAAAATGAGAAATGAAATGAAA
TGAAATGAAATGAAATGAAATGAAATAAAAGAAATAAAAAAATAAATGAAATAAGAAATGAAATGAAATGAAAA
TGAAATGAAATAGAGGAAATGAAATAGAAATGAGGAAATGAAATGAAATGAAATGAAAATAAATGAAATGAAAT
GAAATGAAATGAAATGAAATAGAAATGAGAAATGAAAATGAAATGAAATGAAATAATGAAATGAAATGAGAGGA
AAACAGAAATAAATAGAAATAAGAAATGAAATGAAATGAAATGAAATGAAATGAAATGAAATGAAATGAAATGA
AATGAGAAATGAGGAAATGAAATGAAAATGAAATAATGAAGGAAATAAATGAAATGAAATAGAAATGAAATAAA
TGAAATAAGAAATGAAATGAAATGAGAAATAGAAATGAAATGAAATGAAATAAATGAAATGAAATGAGAAATGA
AATGAGGAAATGAAATGAAATAGAAATGAAAACTGAAATGAAATAGAAATGAAATGAAATGAGGAAATGAAAAT
GAAATGAAAATGAAATAAATAAAAATAATGAAATGAAATGAAATAAGAAATGAAATGAAATGAAATGAAATGAA
ATGAGAAATAGAAATGAAATAATGAGAAATGAAAATGAAAATGAAATGAGAAATGAGAAATGAAATAATGAAAT

GAAAATGAAATAAATGAGAAATAAATGAAATAAAATGAAAATAGAAATGAAAATGAAAATGAAAATGAAAATAA
ATGAAATAAATAAATAAATAAATAGAAATATAAGAAATGAACTTTCACTCCTCCTCCTCCTCACTCATATACTC
ACACTCACTCACTCACTCACTCACTCACTCACTCCCCTCACTCACTCACTCACTCACTCACTCACTCACTCCTC
ACTCACTCACTCCTCACTCACTCACTCACTCACTCACTCACTCACTCACTCACTCACTCTTACTCACTCACTCA
CTCACTCACTCCTCCCTCCTCACTCACTCACTCACTCACTCACTCACTCACTCACTCACTCACTCACTCTCCTC
ACTCACTCACTCTCCTCACTCACTCACTCACTCACTCACTCACTCACTCACTCACTCTCACTCACTCACTCACT
CACTCACTCACTCACTCTCACTCACTCACTCACTCACTCACTCACTCACTCACTCACTCACTCCTCTCTCTTTT
CCACTCACTCACTCACTCACTCACTCTACTCTCACTCACTCTACTCACTCCTCTCACTCACTCTCCTCACTCAC
TCACACTCACTCACTCACTCACTCACTCACTCTCACTCACTCACTCCTCACTCACTCACTCACTCACTCACTCA
CTCACTCACTCACTCCTCTCTACTCCTCCCTTTCTCACTCACTCCTCACTTTCCTCCTCACTCCTCACTCCTCA
CTACTCACTCACTCACTCACTCACTCACTCACTCCTCTACTCCTCACTCACTCACTCCCTCACTCACTCACTCT
ACTCACTCACACTCACTCACTCACTCACTCACTCACTCACTCACTCACTCACACTCACTCACTCACTCACTCAC
TCACTCACTCACACCTCTCTCCTCACTCACTCACTCACTCACTCCTCACTCACTCACTCACTACTCACTCCTCA
CTCACTCACTCACTCACTCACTCACTCACTCACTCACTCACTCACTCACTCCACTCACTCACTCACTCACTCTC
ACTCCTCACTCACTCACTCACTCCACTCCACTCACTCACTCACTCACTCACTCACTCACTCACTCTCCTCTACT
CACTTTCCACTCACTCACTCACTCACTCACTCACTCCACTCACTCACTCACTCACTCTCACTCACTCACTCACT
CACTCACTCACTCACTCACTCACTCACTCACTCTACTCACTCTACTCACTCCACTCACTCACTCACTCACTCAC
TCACTCACTCACTCACTCACTCACTCACTCACTCACTCACTCACTCACTCACTCACTCATAAAACAAAAATAAA
AATAAAAGCAAAAGCAAAATAAGCAAAAGCAAAAGCAAAATAAAATAAAATAAATAAATAAATAAATAAATAAT
GAAATAAAATAGAAGATAAAATGAAATGAAATGAAATGAAATAAAATAAATGAAATAAAATGAAATAAATAAAT
AAATGAAATGAAAATGAAATAAAATAAATAAATAAGAAATAAATAAAATGAAATAAATAAATGAGGAAATAAAT
AAATAAATGAAATGAAATGAAATGAAAATAAGAAATAAGAAAATGAAAATGAAATGAAGTAAATAAATAAATAA
AATAAATGAAAATAAATAGAAATGAAATGAAATAAATAAATGAAATAAATGAAAATGAAAATGAAAATAAATAA
ATGAAATAAAAGAAATGAAAATAAAATAAAAACAAAAACTGAAATAAATGAAAATGAAATAGAAATGAAAATAA
GAAATGAAAATGAAAATGAAATAAATGAAATGAAATGAAATGAAATGAAATGAAATGAAAATGAAAATGAAAAA
TGAAATAAGGAAATGAAATGAAATGAAAATGAAATAAATGAAATAAAATGAAAATAATGAAATGAAATAAATGA
AATGAAATAAATAAATAAATAAATAAGAAATGAAATAAAATAAGAAATGAAATAAATGAAACTAAATGAAAATG
AAATAAATGAAAATGAAATGAAATATAAATGAAATGAAATGAGAGAAATGAAAATGAAATGAAATGAAATGAAA
TGAAATGAAATGAGAAATGAAATGAAAATGAAAATAAGAAATGAAATAAATAAGAAATGAAATAAATGAAATAA
ATAAATAAAATAATGAAATAATAAATGAAATAATGAAATGAAATAAAATGAAATAAAATAAATGAAATGAAATA
AATATGAAATAATGAAATGAGGAAATGAAATGAGAATGAAATGAAATGAAATGAAATGAAATAAATGAAAGAAA
TAAATAAATAAATGAAATGAAAATGAAAATAAATGAAATAAAATGAAATGAAATAGTAAATGAAATGAAATGAA
AATGAAAATGAAATGAAATGAAAGAACCTCTCTGACTTGGAACCTCT

The expansion is highlighted in yellow, with the TTTTA part in black letters and the TTTCA part in red letters. Sequences corresponding to the expected configuration of the expansion (**a-i**) have been trimmed and are shown on the + strand (sequences indicated with a (-) sign have been reverse complemented). **a-d**) Reads sequenced for individual 1-IV-6; **e**) Read sequenced for individual 1-IV-8; **f)** Read sequenced for individual 1-IV-9; **g-h**) Reads sequenced for individual 1-IV-11; **i**) Read sequenced for individual 2-IV-16. **j-m**) Reads obtained for individual 2-IV-9 shown without any modification. The parts with other repeated motifs contained within these sequences likely correspond to basecalling errors. The part highlighted in blue maps to chromosome Xp22.3.
