# Supplementary material for: Unstable TTTTA/TTTCA expansions in MARCH6 are associated with Familial Adult Myoclonic Epilepsy type 3
Source: Nat Commun. 2019 Oct 29;10:4919. doi: 10.1038/s41467-019-12763-9 (PMC6820781; doi:10.1038/s41467-019-12763-9)
Supplement: Supplementary file 5 — Reporting Summary [file 41467_2019_12763_MOESM5_ESM.pdf]

natureresearch

Corresponding author(s): Depienne Christel

Last updated by author(s): 09/04/2019

Reporting Summary

Nature Research wishes to improve the reproducibility of the work that we publish. This form provides structure for consistency and transparency in reporting. For further information on Nature Research policies, see Authors & Referees and the Editorial Policy Checklist.

Statistics

For all statistical analyses, confirm that the following items are present in the figure legend, table legend, main text, or Methods section.

n/a	Confirmed	
☐	☒	The exact sample size (n) for each experimental group/condition, given as a discrete number and unit of measurement
☐	☒	A statement on whether measurements were taken from distinct samples or whether the same sample was measured repeatedly
☐	☒	The statistical test(s) used AND whether they are one- or two-sided<br>*Only common tests should be described solely by name; describe more complex techniques in the Methods section.*
☐	☒	A description of all covariates tested
☐	☒	A description of any assumptions or corrections, such as tests of normality and adjustment for multiple comparisons
☐	☒	A full description of the statistical parameters including central tendency (e.g. means) or other basic estimates (e.g. regression coefficient) AND variation (e.g. standard deviation) or associated estimates of uncertainty (e.g. confidence intervals)
☐	☒	For null hypothesis testing, the test statistic (e.g. F, t, r) with confidence intervals, effect sizes, degrees of freedom and P value noted<br>*Give P values as exact values whenever suitable.*
☒	☐	For Bayesian analysis, information on the choice of priors and Markov chain Monte Carlo settings
☒	☐	For hierarchical and complex designs, identification of the appropriate level for tests and full reporting of outcomes
☐	☒	Estimates of effect sizes (e.g. Cohen's d, Pearson's r), indicating how they were calculated

Our web collection on statistics for biologists contains articles on many of the points above.

Software and code

Policy information about availability of computer code

Data collection	This article did not make use of computer code that is not already available.
Data analysis	The list of already available bioinformatic tools and softwares used in this study are fully described in details in the Material and methods section of the manuscript.

For manuscripts utilizing custom algorithms or software that are central to the research but not yet described in published literature, software must be made available to editors/reviewers. We strongly encourage code deposition in a community repository (e.g. GitHub). See the Nature Research guidelines for submitting code & software for further information.

Data

Policy information about availability of data

All manuscripts must include a data availability statement. This statement should provide the following information, where applicable:

- Accession codes, unique identifiers, or web links for publicly available datasets
- A list of figures that have associated raw data
- A description of any restrictions on data availability

Families included in this study have not consented to have their genome data publicly released. The source data underlying Figs 1, 2b, 3c, 4f, 5a-d and 6b-e, as well as Supplementary Figs 4a, 7, 8 and 12a-b are provided as a Source Data file. Raw images (molecular combing experiments) and raw nanopore data (corresponding to reads included in this study) are available from the corresponding author, upon request. The MARCH6 expansion has been deposited in ClinVar under the accession SCV000924549. RNA-seq and small RNA-seq data have been deposited in the ArrayExpress database at EMBL-EBI (www.ebi.ac.uk/arrayexpress) under accession numbers E-MATB-8300 [https://www.ebi.ac.uk/arrayexpress/experiments/E-MATB-8300] and E-MTAB-8301 [https://www.ebi.ac.uk/arrayexpress/experiments/E-MATB-8301].

Field-specific reporting

Please select the one below that is the best fit for your research. If you are not sure, read the appropriate sections before making your selection.

☒ Life sciences ☐ Behavioural & social sciences ☐ Ecological, evolutionary & environmental sciences

For a reference copy of the document with all sections, see nature.com/documents/nr-reporting-summary-flat.pdf

Life sciences study design

All studies must disclose on these points even when the disclosure is negative.

Sample size	No sample size calculation was made, as this is not applicable to our study. We included all available families with FAME, which is a very rare disorder (about 50 families published to date) in the study.
Data exclusions	Inclusion criteria was families with clinical evidence of FAME. We did not use any exclusion criteria.
Replication	Replication of the initial finding (identification of a TTTTA/TTTCA repeat expansion in MARCH6) was replicated in three additional families with FAME. The observation of the somatic instability was replicated in several independent samples.
Randomization	Not applicable to this study.
Blinding	Genetic testing have been performed using anonymized samples by experimenters blinded to the clinical data.

Reporting for specific materials, systems and methods

We require information from authors about some types of materials, experimental systems and methods used in many studies. Here, indicate whether each material, system or method listed is relevant to your study. If you are not sure if a list item applies to your research, read the appropriate section before selecting a response.

Materials & experimental systems

n/a	Involved in the study	
☐	☒	Antibodies
☒	☐	Eukaryotic cell lines
☒	☐	Palaeontology
☒	☐	Animals and other organisms
☐	☒	Human research participants
☒	☐	Clinical data

Methods

n/a	Involved in the study	
☒	☐	ChIP-seq
☒	☐	Flow cytometry
☒	☐	MRI-based neuroimaging

Antibodies

Antibodies used	Biotinylated Anti-Streptavidin Antibody (BA-0500, VECTOR Laboratories)<br>Anti-MARCH6 antibody (bs-9340R, Bioss antibodies)<br>Anti-beta-Tubulin (#2146, Cell Signaling Technology)
Validation	Anti-Streptavidin antibody used for molecular combing experiments was validated in control individuals without MARCH6 expansions (no red signal was detected).<br>Anti-MARCH6 and anti-beta-tubulin antibodies revealed single bands at the expected size. Anti-beta-Tubulin (#2146) was validated by the provider (Cell Signaling Technology) and is widely used as loading reference.

Human research participants

Policy information about studies involving human research participants

Population characteristics	This article describes familial cases with familial Adult Mycolonic epilepsy (FAME) or Familial Cortical Myoclonic Tremor and Epilepsy (FCMTE)
Recruitment	These families were recruited through specialized Neurology departments in France, Netherlands and Germany.
Ethics oversight	This study has been approved locally by the center that initially identified the family and the overall study was further approved by the ethics committee of the university hospital Essen in April 2018

Note that full information on the approval of the study protocol must also be provided in the manuscript.
