# Supplementary material for: Unstable TTTTA/TTTCA expansions in MARCH6 are associated with Familial Adult Myoclonic Epilepsy type 3
Source: Nat Commun. 2019 Oct 29;10:4919. doi: 10.1038/s41467-019-12763-9 (PMC6820781; doi:10.1038/s41467-019-12763-9)
Supplement: Supplementary file 6 — Description of Additional Supplementary Files [file 41467_2019_12763_MOESM6_ESM.pdf]

Title: Supplementary Data 1
Description: Analysis of RNAseq data in lymphoblastic cells from FAME patients versus control individuals. The table displays raw read counts and normalized gene expression for three affected individuals of Family 5 (FAME2) , three affected individuals and one healthy spouse of Family 1 (FAME3) and three healthy controls. Data are sorted on adjusted pvalue for deregulated genes in FAME3 versus controls.

Title: Supplementary Data 2
Description: Clinical features of Families 1-4 with FAME3 expansions.
Abbreviations: M: Male; F: female; CT: cortical tremor; GTCS: Generalized Tonic-clonic seizures; G: generalized; F: focal; M: myoclonic seizures; AEDs (antiepileptic drugs): BZD: Benzodiazepine; CLB, clobazam; CBZ, carbamazepine; CLN, clonazepam; LEV, levetiracetam; LTG, lamotrigine; OXC, oxcarbamazepine; PB, phenobarbital; PHT, phenytoin; VPA, sodium valproate.

Title: Supplementary Data 3
Description: Sequences obtained from extracted nanopore reads using guppy as Basecaller in six expansion carriers from Families 1 and 2. The expansion is highlighted in yellow, with the TTTTA part in black letters and the TTTCA part in red letters. Sequences corresponding to the expected configuration of the expansion (a-i) have been trimmed and are shown on the + strand (sequences indicated with a (-) sign have been reverse complemented). a-d) Reads sequenced for individual 1-IV-6; e) Read sequenced for individual 1-IV-8; f) Read sequenced for individual 1-IV-9; g-h) Reads sequenced for individual 1-IV-11; i) Read sequenced for individual 2-IV-16. j-m) Reads obtained for individual 2-IV-9 shown without any modification. The parts with other repeated motifs contained within these sequences likely correspond to basecalling errors. The part highlighted in blue maps to chromosome Xp22.3.
